# Supplementary material for: Frameworks, measures, and interventions for HIV-related internalised stigma and stigma in healthcare and laws and policies: systematic review protocol
Source: BMJ Open. 2021 Dec 8;11(12):e053608. doi: 10.1136/bmjopen-2021-053608 (PMC8663079; doi:10.1136/bmjopen-2021-053608)
Supplement: Supplementary data [file bmjopen-2021-053608supp001.pdf]

Appendix
