# Supplementary material for: Frameworks, measures, and interventions for HIV-related internalised stigma and stigma in healthcare and laws and policies: systematic review protocol
Source: BMJ Open. 2021 Dec 8;11(12):e053608. doi: 10.1136/bmjopen-2021-053608 (PMC8663079; doi:10.1136/bmjopen-2021-053608)
Supplement: Author's manuscript [file bmjopen-2021-053608.draft_revisions.pdf]

BMJ Open is committed to open peer review. As part of this commitment we make the peer review history of every article we publish publicly available.

When an article is published we post the peer reviewers' comments and the authors' responses online. We also post the versions of the paper that were used during peer review. These are the versions that the peer review comments apply to.

The versions of the paper that follow are the versions that were submitted during the peer review process. They are not the versions of record or the final published versions. They should not be cited or distributed as the published version of this manuscript.

BMJ Open is an open access journal and the full, final, typeset and author-corrected version of record of the manuscript is available on our site with no access controls, subscription charges or pay-per-view fees (http://bmjopen.bmj.com).

If you have any questions on BMJ Open's open peer review process please email info.bmjopen@bmj.com

BMJ Open

Getting to the Heart of Stigma: Systematic Review Protocol

Journal:	*BMJ Open*
Manuscript ID	bmjopen-2021-053608
Article Type:	Protocol
Date Submitted by the Author:	24-May-2021

Complete List of Authors:	Hempel, Susanne; University of Southern California, Southern California Evidence Review Center<br>Ferguson, Laura; University of Southern California, Institute on Inequalities in Global Health<br>Bolshakova, Maria; University of Southern California, Southern California Evidence Review Center<br>Yagyu, Sachi; University of Southern California, Southern California Evidence Review Center<br>Fu, Ning; Shanghai University of Finance and Economics<br>Motala, Aneesa; University of Southern California, Southern California Evidence Review Center<br>Gruskin, Sofia; University of Southern California, Institute on Inequalities in Global Health

Keywords:	HIV & AIDS < INFECTIOUS DISEASES, HEALTH SERVICES ADMINISTRATION & MANAGEMENT, ETHICS (see Medical Ethics)

SCHOLARONE™
Manuscripts

I, the Submitting Author has the right to grant and does grant on behalf of all authors of the Work (as defined in the below author licence), an exclusive licence and/or a non-exclusive licence for contributions from authors who are: i) UK Crown employees; ii) where BMJ has agreed a CC-BY licence shall apply, and/or iii) in accordance with the terms applicable for US Federal Government officers or employees acting as part of their official duties; on a worldwide, perpetual, irrevocable, royalty-free basis to BMJ Publishing Group Ltd ("BMJ") its licensees and where the relevant Journal is co-owned by BMJ to the co-owners of the Journal, to publish the Work in this journal and any other BMJ products and to exploit all rights, as set out in our licence.

The Submitting Author accepts and understands that any supply made under these terms is made by BMJ to the Submitting Author unless you are acting as an employee on behalf of your employer or a postgraduate student of an affiliated institution which is paying any applicable article publishing charge ("APC") for Open Access articles. Where the Submitting Author wishes to make the Work available on an Open Access basis (and intends to pay the relevant APC), the terms of reuse of such Open Access shall be governed by a Creative Commons licence – details of these licences and which Creative Commons licence will apply to this Work are set out in our licence referred to above.

Other than as permitted in any relevant BMJ Author's Self Archiving Policies, I confirm this Work has not been accepted for publication elsewhere, is not being considered for publication elsewhere and does not duplicate material already published. I confirm all authors consent to publication of this Work and authorise the granting of this licence.

For peer review only

Getting to the Heart of Stigma: Systematic Review Protocol

Susanne Hempel, Southern California Evidence Review Center, Preventive Medicine, University of Southern California, Los Angeles, USA; ORCID ID 0000-0003-1597-5110; Susanne.Hempel@med.usc.edu (corresponding author)

Laura Ferguson, Institute on Inequalities in Global Health, University of Southern California, Los Angeles, USA

Maria Bolshakova, Southern California Evidence Review Center, Preventive Medicine, University of Southern California, Los Angeles, USA

Sachi Yagyu, Southern California Evidence Review Center, Preventive Medicine, University of Southern California, Los Angeles, USA

Ning Fu, School of Economics, Shanghai University of Finance and Economics, Shanghai, China

Aneesa Motala, Southern California Evidence Review Center, Preventive Medicine, University of Southern California, Los Angeles, USA

Sofia Gruskin, Institute on Inequalities in Global Health, University of Southern California, Los Angeles, USA

Word count: 3,880 (4,000 max)

For peer review only

ABSTRACT

Introduction: There is strong global commitment to eliminate HIV-related stigma and work in this area continues to evolve. Wide variation exists in frameworks and measures, and many strategies to prevent, reduce, or mitigate stigma have been proposed but critical factors determining success or failure remain elusive.

Methods and analysis: Building on existing knowledge syntheses, we designed a systematic review to identify frameworks, measures, and intervention evaluations aiming to address internalized stigma, stigma and discrimination in healthcare, and stigma and discrimination at the legal or policy level. The review addresses four key questions (KQ): KQ1: Which conceptual frameworks have been proposed to assess internal stigma, stigma and discrimination experienced in healthcare settings, and stigma and discrimination entrenched in national laws and policies? KQ2: Which measures (e.g. assessment scales) of these different types of stigma and discrimination have been proposed and what are their descriptive properties? KQ3: Which interventions have been evaluated that aimed to reduce these types of stigma and discrimination or mitigate their adverse effects and what are the effectiveness and unintended consequences? KQ4: What common 'critical factors for success or failure' can be identified across the interventions that have been evaluated that might inform future interventions? Critical appraisal figures and tables, detailed evidence tables and focused summary of findings tables will summarize the existing evidence and a gap analysis will provide targeted recommendations for future research. We will establish a compendium of frameworks, a comprehensive catalogue of available measures, and a synthesis of intervention characteristics to advance the science of HIV-related stigma.

Ethics and dissemination: The is registered in PROSPERO (CRD42021249348), the protocol incorporates stakeholder input, the results will be published in a journal manuscript, and the data will be made available in the Systematic Review Data Repository.

290 (max 300)

ARTICLE SUMMARY

Strengths and limitations of this study

- Building on existing knowledge syntheses, we designed a systematic review to identify frameworks, measures, and intervention evaluations addressing HIV-related stigma
- Four key questions guide this review to addresses internalized stigma, stigma and discrimination in healthcare, and stigma and discrimination at the legal or policy level
- We will establish a compendium of frameworks, a comprehensive catalogue of measures, and a synthesis of intervention characteristics to advance the science of HIV-related stigma

KEY WORDS

AIDS, HIV, Systematic Review, Protocol

For peer review only

INTRODUCTION

Stigma is derived from a Greek word meaning a mark or stain. Stigma can be described as a dynamic process of devaluation that significantly discredits an individual in the eyes of others, such as when certain attributes are seized upon within particular cultures or settings and defined as discreditable or unworthy. The term stigma is often used in the literature to encompass both stigma and discrimination even as these are conceptually distinct. While stigma refers to an attitude or belief, discrimination is the behavior or action that results from those attitudes or beliefs. Hence, when stigma is acted upon, the result can be discrimination. Discrimination may refer to any form of arbitrary distinction, exclusion or restriction affecting a person, usually (but not only) because of an inherent personal characteristic or perceived membership of a particular group.

HIV-related stigma has been defined by the Joint United Nations Programme on HIV/AIDS (UNAIDS) as negative beliefs, feelings and attitudes towards people living with HIV, groups associated with people living with HIV (e.g. families of people living with HIV), and other key populations at higher risk of HIV infection, such as people who inject drugs, sex workers, men who have sex with men, and transgender people.[1] Although there is no universal consensus as to their categorization, and many perceive these to be along a continuum, different domains have been identified within HIV-related stigma, including internalized, anticipated, perceived, enacted, externalized, and structural stigma. Different types of stigmas can be experienced and assessed both alone and in combination, each of which is experienced differently and therefore must be addressed differently. Discrimination, as defined under international human rights law, is any distinction, exclusion or restriction based indirectly or directly on grounds prohibited under international law, which has the effect or intent of nullifying the recognition, enjoyment or exercise on an equal basis of others of all human rights and fundamental freedoms, in the political, economic, social, cultural, civil or any other field. In the case of HIV, this can be discrimination based on a person's real or perceived HIV-positive status, irrespective of whether or not there is any justification for these measures.[2] It can rise to the level of a human rights violation. HIV-related discrimination is therefore any distinction, exclusion or restriction (sometimes referred to as acts or omissions) based indirectly or directly on a person's real or perceived HIV status.[3]

There is strong global commitment to eliminate HIV-related stigma, starting with global political commitments and reflected in global and national strategies as well as the multitude of organizations and collaborations working to address stigma. Yet, learning across interventions designed to mitigate against the experience and harmful impacts of stigma is limited by the multitude of evaluation frameworks and measures in use to assess the different dimensions of stigma. For example, a recent review of interventions to address self-stigma did not include a formal meta-analysis due to the heterogeneity of measures used, with eight different scales used across 20 studies.[2] A 2015 UNAIDS report documented over 60 tools to assess and/or address stigma and discrimination within healthcare settings.[4] Assessment of stigma and discrimination entrenched in laws and policies also takes multiple forms including the People Living with HIV Stigma Index, Module 6 of the Demographic and Health Survey, the Integrated Bio-Behavioural Survey, legal environment assessments, the Global Fund baseline assessment methodologies, and the National Commitments and Policy Instrument of the Global AIDS Monitoring process.

Understanding the state of the research in relation to measurement of self-stigma, in accessing health services, and in laws and policies, is needed to help inform future efforts, at all levels, to better address stigma and support people's health and well-being. This review will systematically identify and assess frameworks, measures and interventions of HIV-related individual internalized stigma; both stigma and discrimination within healthcare settings; and stigma and discrimination at the legal and policy level. The intersectionality of HIV-related stigma with other forms of stigma such as stigma relating to 'key' and 'vulnerable' populations that are disproportionately affected by HIV is critical. The review will focus on HIV-related stigma itself, acknowledging as possible where intersectionality is addressed and, specifically, if there appear to be particular gaps in attention to stigma with respect to specific population groups.

Review questions

The systematic review will be guided by four Key Questions:

- Key Question 1: Which conceptual frameworks have been proposed to assess internal stigma, stigma and discrimination experienced in healthcare settings, and stigma and discrimination entrenched in national laws and policies?

For peer review only - http://bmjopen.bmj.com/site/about/guidelines.xhtml

- Key Question 2: Which measures (e.g. assessment scales) of these different types of stigma and discrimination have been proposed and what are their descriptive properties?
- Key Question 3: Which interventions have been evaluated that aimed to reduce these types of stigma and discrimination or mitigate their adverse effects and what are the effectiveness and unintended consequences of the interventions?
- Key Question 4: What common ‘critical factors for success or failure’ can be identified across the interventions that have been evaluated that might inform future interventions?

METHODS AND ANALYSIS

The systematic review will follow this protocol, the review is registered in PROSPERO (CRD42021249348), and the reporting of the protocol and the review will follow the PRISMA guidelines. The systematic review is part of a larger project undertaken by the International AIDS Society (IAS). It is accompanied by four national initiatives to explore stigma and discrimination in the local contexts of Kenya, Malawi, South Africa, and Zambia through key informant interviews and grey literature searches.

The systematic review will follow a transparent and rigorous procedure to minimize review selection and reporting bias. We will search multiple disciplinary and interdisciplinary sources to ensure all relevant studies are captured. Citations and full text publications will be screened by independent literature reviewers to reduce reviewer errors and bias. Eligibility decisions, including reasons for exclusions, will be tracked in citation management software. Data abstraction and critical appraisal will be conducted in online software designed for systematic reviews using detailed, pilot-tested forms with clear reviewer instructions to avoid ambiguity and ensure replicability of coding decisions. The collected data will be accessible in a review data repository.[5]

Context

Our systematic review will be embedded in the context of existing research syntheses. To date, a substantial number of systematic reviews has been published that address different aspects of HIV-related stigma and discrimination. These include associations of stigma (covariates, causes or effects),[6-33] access to care,[34-59] HIV testing,[60-84] country-specific explorations of stigma or discrimination,[85-102] the role of stigma and discrimination in treatment adherence,[103-120] HIV experiences of people living with HIV or their care givers,[121-134] stigma/discrimination reduction in the community,[135-146] HIV disclosure considerations,[147-155] stigma/discrimination in healthcare,[156-163] rights and regulations,[164-170] intersectionality,[171-176] measuring stigma,[177-182] HIV prevention,[183-186] stigma reduction in low income countries,[187-190] self-stigma,[2 191 192] and other, unique topics.[193 194] We build on these reviews which outline existing research and point to persistent knowledge gaps. The reviews will be instrumental for reference-mining to ensure that all relevant material has been considered.

We will identify and categorize existing systematic reviews to explore the research field further. Systematic reviews will be identified through PubMed (biomedical literature) using the systematic review filter, through PsycINFO (psychosocial literature) and Web of science (general science literature including legal and policy analysis), as well as through the Cochrane Database of Systematic Reviews (focus on health) and the Campbell Collaboration (focus on social sciences). Furthermore, we will search the review registries PROSPERO and Open Science Framework to ensure that all relevant registered systematic reviews have been identified.

Analytic framework

Given that there is no agreed nomenclature in this interdisciplinary field, we established working definitions of the concepts ‘stigma,’ ‘internalized stigma’ and ‘discrimination’ for the purpose of this systematic review:

- Stigma refers to beliefs and/or attitudes about HIV.
- Discrimination refers to the behaviors that result from attitudes or beliefs about HIV.
- Internalized stigma (self-stigma) refers to a person living with HIV internalizing negative attitudes associated with HIV and accepting these as applicable to themselves.
- Stigma and discrimination in healthcare refers to negative beliefs and behaviors based on perceived or actual HIV status experienced in healthcare delivery settings.

For peer review only - http://bmjopen.bmj.com/site/about/guidelines.xhtml

- Stigma in laws and policies refers to distinctions, exclusion or restriction based on perceived HIV status or membership of a group that is vulnerable to HIV.

The analytic framework depicted in Figure 1 maps the review's four Key Questions and outlines the review's content, i.e., frameworks, measures, and intervention evaluations, targeted in this systematic review.

The figure includes existing conceptual models for HIV-related stigma, including frameworks such as the global HIV stigma reduction framework,[195 196] and logic models addressing stigma and discrimination, potential causes or effects (Key question 1). The frameworks comprise of the underlying construct of stigma and/or discrimination and their components. The analytic framework shows also the measures, indicators, or operationalizations of the constructs stigma and discrimination that are used to measure and quantify stigma and discrimination (Key question 2). The arrow represents interventions aiming to prevent, reduce, or mitigate stigma and discrimination (Key question 3), as well as the 'critical success factors' of tested interventions (Key question 4).

The systematic review is focused on people with living or perceived to be living with HIV and people who are disproportionately affected by HIV. For the purposes of this review and based on the UNAIDS definition, the latter group includes gay men and other men who have sex with men, sex workers, transgender people, people who inject drugs and prisoners and other incarcerated people.[197]

Search strategy

To identify primary research studies, we will search the health-specific research database PubMed, in particular to identify research on stigma experienced in healthcare settings. We will search PsycINFO to identify psychological and social research on stigma and use the general scientific research database Web of Science, in particular to identify legal and policy analyses on stigma and discrimination. We will identify government and non-governmental organization reports indexed in the Universal Human Rights Index, HeinOnline, PAIS, and HIV Legal Network.

Additional grey literature searches will target organizations relevant to key populations affected by HIV and funders of stigma research. Specifically, we will search the websites of the IAS, UNAIDS, United Nations Development Programme (UNDP), STRIVE, Health Policy Plus, and Sage (resource-sharing community for Canadian HIV and hepatitis C service providers).

Targeted search strategies for each Key Question will combine free text search terms with controlled vocabulary of the individual databases. Draft search strategies are shown in the appendix. The searches will be designed, executed, and documented by an Evidence-based Practice Center librarian. The searches outside of research databases will be instrumental in identifying conceptual models to assess internal stigma and stigma entrenched in national laws and policies. The multiple sources and interdisciplinary approach aim to reduce selection bias being introduced in the literature review.

Eligibility criteria

We use a PICOTS (participant, independent variable, comparator or study design, outcome/measure, timing, and setting) framework to structure the eligibility criteria:

- Participants: People living with or perceived to be living with HIV and people from groups who are disproportionately affected by HIV infection. We will exclude studies of mixed populations and other participant targets unless the study provides HIV-relevant subgroup analyses.
- Independent variable: Frameworks will include models outlining and relating to multiple components of stigma and/or discrimination, including conceptual frameworks, logic models, taxonomies, and analytic models for assessment, prevention, reduction, or mitigation of stigma. Measures will include self and peer report measures used for formal assessment of stigma and discrimination. Studies using a published measure will be included if the psychometric properties of the measure are a central focus of the study (e.g., evaluating the predictive validity of an existing measure). Eligible intervention evaluations may test strategies and policies aimed at preventing, reducing, or mitigating HIV-related stigma and discrimination. Intervention evaluations aimed at self-stigma, stigma and discrimination in healthcare, and stigma and discrimination at the legal or policy level are eligible. We will exclude publications exclusively addressing community and cultural stigma.
- Comparator / study design: Publications introducing frameworks will be included regardless of the comparator or study design. Measure research needs to describe the tool in sufficient detail to be included but needs no comparator. Experimental evaluations of interventions have

For peer review only - http://bmjopen.bmj.com/site/about/guidelines.xhtml

to report on a concurrent comparator (e.g., randomized controlled trial, controlled clinical trial, comparative studies, quasi-experimental studies, natural experiments) to be eligible. Observational evaluations with interventions or policies not under the control of the investigator and influenced by secular trends, have to report on a historic or concurrent comparator (e.g., pre-post, cohort study comparing two cohorts) and be sufficiently large or encompassing to be eligible (either demonstrating statistical power in a power analysis, reporting on large-scale evaluations of 200 participants or more, or ensuring that all eligible units have been targeted such as all healthcare providers in a hospital received the intervention). For studies evaluating the impacts of laws, no comparator is required if the study author can demonstrate an alternative analysis of determining the effect of the law not compared to a prior period or different legislative environment. Systematic reviews will be retained for an umbrella review providing context and for reference-mining.

- Outcome: Framework publications will be included regardless of any reported outcomes. Measure research needs to report a detailed description of the measure, the development process, or the evaluation or validation of the measure. Intervention research needs to provide a structured evaluation of an indicator of stigma or operationalization of discrimination to be eligible.
- Timing: Frameworks will not be restricted by publication year. For measures and interventions, only those published from 2008 on will be included, building on the first People Living with HIV Stigma Index published in 2008, which transformed thinking around HIV-related stigma measurement.[198]
- Setting: The review is not restricted regarding setting but we will restrict to English language publications.

Data abstraction

For the frameworks, we will abstract the author group and publisher; publication year, scope, aim or purpose of the framework; subtype and definition of the constructs stigma and/or discrimination; addressed targets (e.g., people living with HIV and their families); framework components; and a broad summary of the framework. The Draft Evidence Table Key Question 1 will summarize the identified frameworks (see appendix).

For measures, we will document the author group; publication year; name of the tool; the stigma or discrimination subtype being assessed, the underlying framework (where applicable) and definitions of stigma and discrimination; the targeted population; the surveyed population used to develop or assess the measure; the scale structure of the tool, number of items, and answer mode; the documented reliability; and evidence of validity. Draft Evidence Table Key Question 2 outlines the evidence table documenting this information for each included publication (see appendix).

For intervention evaluations (Key Question 3), we will document the study identification details, year of publication, country, study design, sample size, participant details, context or setting; intervention type, intervention description, and intervention components, comparator type and comparator description, definition and measures of stigma and discrimination, and findings for the outcome measures. In order to document the effect of the intervention concisely, we will abstract data for the main effectiveness signal and adverse events or unintended consequences. In addition, we will record any information provided by the study authors on the appropriateness of the utilized measures, intervention or comparator. The draft evidence table is shown in the appendix (Draft Evidence Table Key Question 3).

Critical appraisal and analysis plan

For frameworks, we will assess the source (e.g., published by an individual author group or endorsement by a professional organization) and processes used to develop the framework, including stakeholder involvement and formal consensus finding methods.

For measures, we will evaluate the demonstrated reliability (internal consistency, test-retest stability, rater agreement) as well as evidence of the validity (e.g., content, criterion, or construct validity) applying COSMIN (COnsensus-based Standards for the selection of health Measurement Instruments) criteria.[199]

Intervention evaluations will be evaluated for potential selection, detection, performance, attrition, reporting, and study-specific sources of bias, adapting RoB 2 and ROBINS-I criteria.[200 201]

For peer review only - http://bmjopen.bmj.com/site/about/guidelines.xhtml

To explore which interventions are successful and to determine what characterizes these interventions, we will first broadly categorize the type of intervention. The categorization will be undertaken based on the abstracted intervention description and will be blind to the findings of the study. The categories will be used to identify subgroups of more homogeneous intervention types. In a second step, we will derive a set of common intervention components drawn from the identified literature and informed by existing intervention frameworks and taxonomies. The presence or absence of components will be documented for each successful and each unsuccessful intervention, together with context information such as the country of evaluation. The draft component tables for the successful and unsuccessful interventions are shown in the appendix (Draft Component Table Key Question 4a, Draft Component Table Key Question 4b).

Categorizing the evaluations as successful or unsuccessful will follow a transparent algorithm and will be determined by two independent literature reviewers to reduce errors and bias. For all studies, the reduction of stigma will be determined. In the absence of reporting on stigma, the primary outcome according to the study authors will be used to determine success or failure of the intervention in the research study. Effect estimates will be based on the results in the intervention group relative to a control group. In the absence of a concurrent control group, the change compared to the pre-intervention status will be used to determine intervention success. We will compute measure-independent effect estimates for all included studies, i.e., standardized mean differences (SMD) for continuous outcomes and relative risks (RR) for categorical outcomes, to facilitate comparisons across studies. Interpretation of the effect sizes will take the statistical significance of the difference compared to the control or pre-intervention status into account as well as the statistical power of the study to detect an effect. Discrepancies in the effect classification between reviewer ratings will be resolved through discussion in the review team.

Determining characteristics of successful interventions and success factors in often complex and multi-component interventions, as well as reasons for failure, will require a careful analysis of the components or ‘active ingredients’ of the interventions. We will apply principles of qualitative comparative analysis (QCA) and review the established component matrix across the successful interventions in seeking to determine which individual and figurations of factors appear to be associated with success.[202] We will explore differences in the component structure between interventions that were determined to be successful and those that were determined to be unsuccessful to identify components likely be associated with success. We will conduct meta-regressions across studies to confirm effects of components. Meta-regressions will add the component of interest to the meta-analytic model to determine whether its presence or absence affects the size of the intervention effect.

Summary of findings and body of evidence assessment

We will document transparent criteria to evaluate the certainty in the evidence across the included research. We will adapt the eight GRADE (Grading of Recommendations, Assessment, Development, and Evaluation) criteria *study limitation, inconsistency, imprecision, indirectness, reporting bias to upgrade and the criteria large effect, dose-response relationship, and confounding would mask an effect*, to downgrade the quality of evidence.[203] While the criterion *study limitation* is applicable to all key questions, *inconsistency* (e.g., of reliability estimates across studies) will be used to interpret the quality of evidence for Key Question 2, 3, and 4, and the criterion *imprecision* (e.g., range of reported reliability estimates) will be applied to Key Question 2 and 3. All other criteria will primarily apply to Key Question 3 and the evidence statements that can be formulated to answer this key question.

The evaluation of the body of evidence will be used to arrive at internationally accepted certainty categories that communicate our confidence in the findings using the categories *high*, *moderate, low*, and *very low*. *High* indicates confidence that the true effect is similar to the estimated effect, *moderate* indicates the true effect is probably close to the estimated effect, *low* suggests the true effect might be markedly different from the estimated effect, and *very low* signals that the true effect is probably markedly different from the estimated effect. We will review the appropriateness of the starting point of low quality of evidence for non-randomized studies before upgrading or downgrading of the evidence to avoid floor effects and ensure meaningful differentiation.

Synthesis

The identified evidence will be documented in comprehensive tables and figures. The literature flow will be documented in a literature flow diagram and account for all identified research. Critical appraisal of frameworks and measures will be integrated into the evidence tables. The risk of bias

For peer review only - http://bmjopen.bmj.com/site/about/guidelines.xhtml

across intervention studies will be documented in a risk of bias figure showing the distribution for each criterion. All included research will be documented in concise evidence tables providing details for each included framework, measure, and intervention evaluation (see drafts in the appendix).

With this review, we intend to establish a compendium of existing frameworks for HIV-related stigma and discrimination. Frameworks published under Creative Commons licenses, and where copyright agreements allow these for other frameworks, will be shown in full to allow a meaningful overview. Where copyright assertions cannot be obtained, the framework evidence table will include the link to the framework if available in the public domain.

The evidence table for measures aims to provide a resource for future HIV stigma and discrimination research. It will provide a comprehensive overview of the available measures to help select tools for future studies.

The evidence table and component tables for the identified interventions aim to document the existing knowledge base for interventions to address self-stigma, stigma and discrimination in healthcare, and stigma and discrimination in laws and policies. A detailed exploration of intervention components aims to support efforts in prevention, reducing and mitigating HIV-related stigma and discrimination.

In addition to a narrative synthesis, we will document the evidence across research in four summary of findings tables with one table for each key question. The table summarizing Key Question 1 will document the number and type of the identified stigma and discrimination frameworks. The summary of findings table summarizing Key Question 2 will document the number and type of identified stigma and discrimination measures, listing the measure details and reliability (evidence that the tool is measuring accurately and consistently) and validity (evidence that the tool is measuring what it is supposed to measure). The third table will document the evidence for the interventions (Key Question 3), organized by context and modality (e.g., healthcare facility intervention, law or policy change), and within by outcome category (effectiveness, unintended consequences), together with the quality of evidence assessment. The fourth summary of findings table will document commonalities of successful interventions and failed interventions (Key Question 4).

Gap analysis

Part of the systematic review will be a formal gap analysis. We will use a structured approach to document research gaps. Gaps will be documented in a Study design, Participants, Interventions, Framework, Outcomes, Context / Country (SPIFOC) framework, with attention to other relevant categories that may emerge during the evidence review. The gap analysis will document research needs and provide concrete recommendations for future research.

Patient and public involvement statement

The draft protocol was peer-reviewed by international content experts and a representative of the community of persons living with HIV to ensure that the review asks the right questions. Stakeholders will be asked to review the draft review to ensure that all relevant frameworks, measures, and intervention evaluations have been captured and that the review contributes meaningfully to the knowledge base and to ensure that the evidence review is as impactful as possible.

ETHICS AND DISSEMINATION

The systematic review conduct will be transparent and is designed to advance research to support people living with or suspected to be living with HIV and people disproportionally affected by HIV.

The review will be registered in PROSPERO, the results will be published in a journal manuscript and presented at research conferences. The data will be made available in the Systematic Review Data Repository.[5]

REFERENCES

1. Joint United Nations Programme on HIV/AIDS. Reduction of HIV-related stigma and discrimination: Guidance note. Geneva, Switzerland: UNAIDS,, 2014.

For peer review only

2. Pantelic M, Steinert JI, Park J, et al. 'Management of a spoiled identity': systematic review of interventions to address self-stigma among people living with and affected by HIV. *BMJ Glob Health* 2019;4(2):e001285. doi: 10.1136/bmjgh-2018-001285 [published Online First: 2019/04/19]
3. UNAIDS. Evidence for eliminating HIV-related stigma and discrimination: Joint United Nations Programme on HIV/AIDS (UNAIDS), 2020.
4. UNAIDS. Mapping of tools on HIV-related stigma and discrimination in health care settings: Final Report, 2015.
5. Agency for Healthcare Research and Quality. SRDR Systematic Review Data Reposity [cited Protocol ref. Available from: https://srdr.ahrq.gov/ accessed May 11 2021.
6. Amare T, Getinet W, Shumet S, et al. Prevalence and Associated Factors of Depression among PLHIV in Ethiopia: Systematic Review and Meta-Analysis, 2017. *AIDS Res Treat* 2018;2018:5462959. doi: 10.1155/2018/5462959 [published Online First: 2018/04/24]
7. Ayano G, Solomon M, Abraha M. A systematic review and meta-analysis of epidemiology of depression in people living with HIV in east Africa. *BMC Psychiatry* 2018;18(1):254. doi: 10.1186/s12888-018-1835-3 [published Online First: 2018/08/17]
8. Bayat AH, Mohammadi R, Moradi-Joo M, et al. HIV and drug related stigma and risk-taking behaviors among people who inject drugs: a systematic review and meta-analysis. *J Addict Dis* 2020;38(1):71-83. doi: 10.1080/10550887.2020.1718264 [published Online First: 2020/03/19]
9. Belayneh Z, Mekuriaw B, Mehare T, et al. Magnitude and predictors of common mental disorder among people with HIV/AIDS in Ethiopia: a systematic review and meta-analysis. *BMC Public Health* 2020;20(1):689. doi: 10.1186/s12889-020-08800-8 [published Online First: 2020/05/16]
10. Dessauvagie AS, Jörns-Presentati A, Napp AK, et al. The prevalence of mental health problems in sub-Saharan adolescents living with HIV: a systematic review. *Glob Ment Health (Camb)* 2020;7:e29. doi: 10.1017/gmh.2020.18 [published Online First: 2021/01/26]
11. Evangeli M, Ferris K, Kenney NM, et al. A systematic review of psychological correlates of HIV testing intention. *Aids Care-Psychol Socio-Med Asp Aids-Hiv* 2018;30(1):18-26. doi: 10.1080/09540121.2017.1344351
12. Gesesew HA, Tesfay Gebremedhin A, Demissie TD, et al. Significant association between perceived HIV related stigma and late presentation for HIV/AIDS care in low and middle-income countries: A systematic review and meta-analysis. *PLoS One* 2017;12(3):e0173928. doi: 10.1371/journal.pone.0173928 [published Online First: 2017/03/31]
13. Ghiasvand H, Higgs P, Noroozi M, et al. Social and demographical determinants of quality of life in people who live with HIV/AIDS infection: evidence from a meta-analysis. *Biodemography Soc Biol* 2020;65(1):57-72. doi: 10.1080/19485565.2019.1587287 [published Online First: 2019/03/19]
14. Govindasamy D, Seeley J, Olaru ID, et al. Informing the measurement of wellbeing among young people living with HIV in sub-Saharan Africa for policy evaluations: a mixed-methods systematic review. *Health Qual Life Outcomes* 2020;18(1):120. doi: 10.1186/s12955-020-01352-w [published Online First: 2020/05/07]
15. Griswold MK, Pagano-Therrien J. Women Living With HIV in High Income Countries and the Deeper Meaning of Breastfeeding Avoidance: A Metasynthesis. *J Hum Lact* 2020;36(1):44-52. doi: 10.1177/0890334419886565 [published Online First: 2020/01/03]
16. Haines C, Loades ME, Coetzee BJ, et al. Which HIV-infected youth are at risk of developing depression and what treatments help? A systematic review focusing on Southern Africa. *Int J Adolesc Med Health* 2019 doi: 10.1515/ijamh-2019-0037 [published Online First: 2019/08/09]
17. Ishola F. EFFECT OF HIV RELATED STIGMA ON UTILISATION OF SKILLED BIRTH ATTENDANTS BY HIV POSITIVE WOMEN IN NIGERIA; A SYSTEMATIC REVIEW. *Sex Transm Infect* 2017;93:A207-A07. doi: 10.1136/sextrans-2017-053264.538
18. Li H, Li X, Zhang L, et al. Effects of multiple types of stigma on the probability of HIV disclosure to sex partners: a systematic review. *Sex Health* 2016;13(6):516-29. doi: 10.1071/sh16089 [published Online First: 2016/08/06]
19. MacCarthy S, Poteat T, Xia Z, et al. Current research gaps: a global systematic review of HIV and sexually transmissible infections among transgender populations. *Sex Health* 2017;14(5):456-68. doi: 10.1071/sh17096 [published Online First: 2017/12/09]
20. MacLean JR, Wetherall K. The Association between HIV-Stigma and Depressive Symptoms among People Living with HIV/AIDS: A Systematic Review of Studies Conducted in South Africa. *J Affect Disord* 2021;287:125-37. doi: 10.1016/j.jad.2021.03.027 [published Online First: 2021/03/30]

For peer review only - http://bmjopen.bmj.com/site/about/guidelines.xhtml

21. Magno L, Silva L, Veras MA, et al. Stigma and discrimination related to gender identity and vulnerability to HIV/AIDS among transgender women: a systematic review. *Cad Saude Publica* 2019;35(4):e00112718. doi: 10.1590/0102-311x00112718 [published Online First: 2019/04/18]
22. Narasimhan M, Payne C, Caldas S, et al. Ageing and healthy sexuality among women living with HIV. *Reprod Health Matters* 2016;24(48):43-51. doi: 10.1016/j.rhm.2016.11.001 [published Online First: 2016/12/28]
23. Necho M, Belete A, Tsehay M. Depressive symptoms and their determinants in patients who are on antiretroviral therapy in the case of a low-income country, Ethiopia: a systematic review and meta-analysis. *Int J Ment Health Syst* 2021;15(1):3. doi: 10.1186/s13033-020-00430-2 [published Online First: 2021/01/08]
24. Necho M, Tsehay M, Zenebe Y. Suicidal ideation, attempt, and its associated factors among HIV/AIDS patients in Africa: a systematic review and meta-analysis study. *Int J Ment Health Syst* 2021;15(1):13. doi: 10.1186/s13033-021-00437-3 [published Online First: 2021/01/25]
25. Pantelic M, Shenderovich Y, Cluver L, et al. Predictors of internalised HIV-related stigma: a systematic review of studies in sub-Saharan Africa. *Health Psychol Rev* 2015;9(4):469-90. doi: 10.1080/17437199.2014.996243 [published Online First: 2015/01/07]
26. Rueda S, Mitra S, Chen S, et al. Examining the associations between HIV-related stigma and health outcomes in people living with HIV/AIDS: a series of meta-analyses. *BMJ Open* 2016;6(7):e011453. doi: 10.1136/bmjopen-2016-011453 [published Online First: 2016/07/15]
27. Rzeszutek M, Gruszczyńska E. Posttraumatic growth among people living with HIV: A systematic review. *J Psychosom Res* 2018;114:81-91. doi: 10.1016/j.jpsychores.2018.09.006 [published Online First: 2018/10/14]
28. Scofield D, Moseholm E. HIV-related stigma and health-related quality of life in women living with HIV in developed countries: a systematic review. *Aids Care-Psychol Socio-Med Asp Aids-Hiv* 2021:9. doi: 10.1080/09540121.2021.1891193
29. Scott W, Arkuter C, Kioskli K, et al. Psychosocial factors associated with persistent pain in people with HIV: a systematic review with meta-analysis. *Pain* 2018;159(12):2461-76. doi: 10.1097/j.pain.0000000000001369 [published Online First: 2018/08/22]
30. Yassin Z, Erasmus C, Frantz J. HIV-related stigma and the psychosocial well-being of children orphaned by AIDS: A systematic review. *Vulnerable Children and Youth Studies* 2018;13(3):247-58. doi: http://dx.doi.org/10.1080/17450128.2018.1457193
31. Yassin Z, Erasmus CJ. The impact of HIV-related stigma on the psychological well-being of children who have been orphaned by AIDS. *Vulnerable Children and Youth Studies* 2016;11(4):297-323. doi: http://dx.doi.org/10.1080/17450128.2016.1214769
32. Zgambo M, Kalembo FW, Mbakaya BC. Risky behaviours and their correlates among adolescents living with HIV in sub-Saharan Africa: a systematic review. *Reprod Health* 2018;15(1):180. doi: 10.1186/s12978-018-0614-4 [published Online First: 2018/10/26]
33. Zhao Y, Luo T, Tucker JD, et al. Risk Factors of HIV and Other Sexually Transmitted Infections in China: A Systematic Review of Reviews. *PLoS One* 2015;10(10):e0140426. doi: 10.1371/journal.pone.0140426 [published Online First: 2015/10/16]
34. Aggarwal NK, Consavage KE, Dhanuka I, et al. Health and Health Care Access Barriers Among Transgender Women Engaged in Sex Work: A Synthesis of US-Based Studies Published 2005-2019. *Lgbt Health* 2021;8(1):11-25. doi: 10.1089/lgbt.2019.0243
35. Ahmed S, Autrey J, Katz IT, et al. Why do people living with HIV not initiate treatment? A systematic review of qualitative evidence from low- and middle-income countries. *Soc Sci Med* 2018;213:72-84. doi: 10.1016/j.socscimed.2018.05.048 [published Online First: 2018/07/31]
36. Bass SB, D'Avanzo P, Alhajji M, et al. Exploring the Engagement of Racial and Ethnic Minorities in HIV Treatment and Vaccine Clinical Trials: A Scoping Review of Literature and Implications for Future Research. *Aids Patient Care and Stds* 2020;34(9):399-416. doi: 10.1089/apc.2020.0008
37. Brookfield S, Dean J, Forrest C, et al. Barriers to Accessing Sexual Health Services for Transgender and Male Sex Workers: A Systematic Qualitative Meta-summary. *AIDS Behav* 2020;24(3):682-96. doi: 10.1007/s10461-019-02453-4 [published Online First: 2019/03/15]
38. Ferguson L, Grant AD, Watson-Jones D, et al. Linking women who test HIV-positive in pregnancy-related services to long-term HIV care and treatment services: a systematic review. *Trop Med Int Health* 2012;17(5):564-80. doi: 10.1111/j.1365-3156.2012.02958.x [published Online First: 2012/03/08]

For peer review only - http://bmjopen.bmj.com/site/about/guidelines.xhtml

39. Ganle JK, Baatiema L, Quansah R, et al. Barriers facing persons with disability in accessing sexual and reproductive health services in sub-Saharan Africa: A systematic review. *Plos One* 2020;15(10):19. doi: 10.1371/journal.pone.0238585
40. Gari S, Doig-Acuña C, Smail T, et al. Access to HIV/AIDS care: a systematic review of socio-cultural determinants in low and high income countries. *BMC Health Serv Res* 2013;13:198. doi: 10.1186/1472-6963-13-198 [published Online First: 2013/05/30]
41. Gonçalves TR, Costa AHC, Sales MS, et al. [Combined HIV prevention? Systematic review of interventions with women from low- and middle-income countries]. *Cien Saude Colet* 2020;25(5):1897-912. doi: 10.1590/1413-81232020255.15832018 [published Online First: 2020/05/14]
42. Gourlay A, Birdthistle I, Mburu G, et al. Barriers and facilitating factors to the uptake of antiretroviral drugs for prevention of mother-to-child transmission of HIV in sub-Saharan Africa: a systematic review. *J Int AIDS Soc* 2013;16(1):18588. doi: 10.7448/ias.16.1.18588
18588 [published Online First: 2013/07/23]
43. Govindasamy D, Ford N, Kranzer K. Risk factors, barriers and facilitators for linkage to antiretroviral therapy care: a systematic review. *Aids* 2012;26(16):2059-67. doi: 10.1097/QAD.0b013e3283578b9b [published Online First: 2012/07/12]
44. Hodgson I, Plummer ML, Konopka SN, et al. A systematic review of individual and contextual factors affecting ART initiation, adherence, and retention for HIV-infected pregnant and postpartum women. *PLoS One* 2014;9(11):e111421. doi: 10.1371/journal.pone.0111421 [published Online First: 2014/11/06]
45. Jones C, Ritchwood TD, Taggart T. Barriers and Facilitators to the Successful Transition of Adolescents Living with HIV from Pediatric to Adult Care in Low and Middle-Income Countries: A Systematic Review and Policy Analysis. *AIDS Behav* 2019;23(9):2498-513. doi: 10.1007/s10461-019-02621-6 [published Online First: 2019/08/05]
46. Kendrick HM. Are religion and spirituality barriers or facilitators to treatment for HIV: a systematic review of the literature. *Aids Care-Psychol Socio-Med Asp Aids-Hiv* 2017;29(1):1-13. doi: 10.1080/09540121.2016.1201196
47. Lancaster KE, Cernigliaro D, Zulliger R, et al. HIV care and treatment experiences among female sex workers living with HIV in sub-Saharan Africa: A systematic review. *Afr J AIDS Res* 2016;15(4):377-86. doi: 10.2989/16085906.2016.1255652 [published Online First: 2016/12/16]
48. Matacotta JJ, Rosales-Perez FJ, Carrillo CM. HIV Preexposure Prophylaxis and Treatment as Prevention - Beliefs and Access Barriers in Men Who Have Sex With Men (MSM) and Transgender Women: A Systematic Review. *J Patient Cent Res Rev* 2020;7(3):265-74. doi: 10.17294/2330-0698.1737 [published Online First: 2020/08/08]
49. Mathias A, Alves Dos Santos L, Grangeiro A, et al. Thematic synthesis HIV prevention qualitative studies in men who have sex with men (MSM). *Colomb Med (Cali)* 2019;50(3):201-14. doi: 10.25100/cm.v50i3.4078 [published Online First: 2020/04/15]
50. Mwaturura C, Traeger M, Lemoh C, et al. Barriers and facilitators to pre-exposure prophylaxis among African migrants in high income countries: a systematic review. *Sexual Health* 2021:10. doi: 10.1071/sh20175
51. O'Brien N, Hong QN, Law S, et al. Health System Features That Enhance Access to Comprehensive Primary Care for Women Living with HIV in High-Income Settings: A Systematic Mixed Studies Review. *AIDS Patient Care STDS* 2018;32(4):129-48. doi: 10.1089/apc.2017.0305 [published Online First: 2018/04/10]
52. Pellowski JA. Barriers to care for rural people living with HIV: a review of domestic research and health care models. *J Assoc Nurses AIDS Care* 2013;24(5):422-37. doi: 10.1016/j.jana.2012.08.007 [published Online First: 2013/01/29]
53. Peng P, Su S, Fairley CK, et al. A Global Estimate of the Acceptability of Pre-exposure Prophylaxis for HIV Among Men Who have Sex with Men: A Systematic Review and Meta-analysis. *AIDS Behav* 2018;22(4):1063-74. doi: 10.1007/s10461-017-1675-z [published Online First: 2017/02/09]
54. Rade DA, Crawford G, Lobo R, et al. Sexual Health Help-Seeking Behavior among Migrants from Sub-Saharan Africa and South East Asia living in High Income Countries: A Systematic Review. *Int J Environ Res Public Health* 2018;15(7) doi: 10.3390/ijerph15071311 [published Online First: 2018/06/23]

For peer review only - http://bmjopen.bmj.com/site/about/guidelines.xhtml

55. Russ S, Zhang C, Liu Y. Pre-Exposure Prophylaxis Care Continuum, Barriers, and Facilitators among Black Men Who Have Sex with Men in the United States: A Systematic Review and Meta-Analysis. *Aids and Behavior* 2020:11. doi: 10.1007/s10461-020-03156-x
56. Scholl E. Improving outpatient implementation of preexposure prophylaxis in men who have sex with men. *Journal of the American Association of Nurse Practitioners* 2016;28(8):446-52. doi: http://dx.doi.org/10.1002/2327-6924.12344
57. Wang Z, Yuan T, Fan S, et al. HIV Nonoccupational Postexposure Prophylaxis Among Men Who Have Sex with Men: A Systematic Review and Meta-Analysis of Global Data. *AIDS Patient Care STDS* 2020;34(5):193-204. doi: 10.1089/apc.2019.0313 [published Online First: 2020/05/13]
58. Wao H, Aluoch M, Odondi GO, et al. MSM's Versus Health Care Providers' Perceptions of Barriers to Uptake of HIV/AIDS-Related Interventions: Systematic Review and Meta-Synthesis of Qualitative and Quantitative Evidence. *Int J Sex Health* 2016;28(2):151-62. doi: 10.1080/19317611.2016.1153560
59. Young T, Busgeeth K. Home-based care for reducing morbidity and mortality in people infected with HIV/AIDS. *Cochrane Database Syst Rev* 2010(1):Cd005417. doi: 10.1002/14651858.CD005417.pub2 [published Online First: 2010/01/22]
60. Alvarez-del Arco D, Monge S, Azcoaga A, et al. HIV testing and counselling for migrant populations living in high-income countries: a systematic review. *Eur J Public Health* 2013;23(6):1039-45. doi: 10.1093/eurpub/cks130 [published Online First: 2012/09/25]
61. Blackstone SR, Nwaozuru U, Iwelunmor J. Antenatal HIV Testing in Sub-Saharan Africa During the Implementation of the Millennium Development Goals: A Systematic Review Using the PEN-3 Cultural Model. *Int Q Community Health Educ* 2018;38(2):115-28. doi: 10.1177/0272684x17749576 [published Online First: 2017/12/23]
62. Bolsewicz K, Vallely A, Debattista J, et al. Factors impacting HIV testing: A review—Perspectives from Australia, Canada, and the UK. *AIDS Care* 2015;27(5):570-80. doi: http://dx.doi.org/10.1080/09540121.2014.986050
63. Campbell CK, Lippman SA, Moss N, et al. Strategies to Increase HIV Testing Among MSM: A Synthesis of the Literature. *AIDS Behav* 2018;22(8):2387-412. doi: 10.1007/s10461-018-2083-8 [published Online First: 2018/03/20]
64. Chamla D, Luo C, Adjorlolo-Johnson G, et al. Integration of HIV infant testing into immunization programmes: a systematic review. *Paediatr Int Child Health* 2015;35(4):298-304. doi: 10.1080/20469047.2015.1109233 [published Online First: 2016/01/09]
65. Duffy M, Sharer M, Berhan A, et al. HIV risk screening for high-yield community testing services for orphans and vulnerable children: A literature review. *Vulnerable Children and Youth Studies* 2018;13(2):95-115. doi: http://dx.doi.org/10.1080/17450128.2017.1332399
66. Evangeli M, Pady K, Wroe AL. Which Psychological Factors are Related to HIV Testing? A Quantitative Systematic Review of Global Studies. *AIDS Behav* 2016;20(4):880-918. doi: 10.1007/s10461-015-1246-0 [published Online First: 2015/11/15]
67. Feyissa GT, Lockwood C, Munn Z. The effectiveness of home-based HIV counseling and testing on reducing stigma and risky sexual behavior among adults and adolescents: A systematic review and meta-analyses. *JBI Database System Rev Implement Rep* 2015;13(6):318-72. doi: 10.11124/jbisrir-2015-2235 [published Online First: 2015/10/13]
68. Hamilton A, Shin S, Taggart T, et al. HIV testing barriers and intervention strategies among men, transgender women, female sex workers and incarcerated persons in the Caribbean: a systematic review. *Sex Transm Infect* 2020;96(3):189-96. doi: 10.1136/sextrans-2018-053932 [published Online First: 2019/09/12]
69. Hamilton A, Thompson N, Choko AT, et al. HIV Self-Testing Uptake and Intervention Strategies Among Men in Sub-Saharan Africa: A Systematic Review. *Front Public Health* 2021;9:594298. doi: 10.3389/fpubh.2021.594298 [published Online First: 2021/03/09]
70. Krause J, Subklew-Sehume F, Kenyon C, et al. Acceptability of HIV self-testing: a systematic literature review. *BMC Public Health* 2013;13:735. doi: 10.1186/1471-2458-13-735 [published Online First: 2013/08/09]
71. Laprise C, Bolster-Foucault C. Understanding barriers and facilitators to HIV testing in Canada from 2009-2019: A systematic mixed studies review. *Can Commun Dis Rep* 2021;47(2):105-25. doi: 10.14745/ccdr.v47i02a03 [published Online First: 2021/03/23]
72. Leidel S, Wilson S, McConigley R, et al. Health-care providers' experiences with opt-out HIV testing: a systematic review. *AIDS Care* 2015;27(12):1455-67. doi: 10.1080/09540121.2015.1058895 [published Online First: 2015/08/15]

For peer review only - http://bmjopen.bmj.com/site/about/guidelines.xhtml

73. Lorenc T, Marrero-Guillamón I, Llewellyn A, et al. HIV testing among men who have sex with men (MSM): systematic review of qualitative evidence. *Health Educ Res* 2011;26(5):834-46. doi: 10.1093/her/cyr064 [published Online First: 2011/08/30]
74. Musheke M, Ntalasha H, Gari S, et al. A systematic review of qualitative findings on factors enabling and deterring uptake of HIV testing in Sub-Saharan Africa. *BMC Public Health* 2013;13:220. doi: 10.1186/1471-2458-13-220 [published Online First: 2013/03/19]
75. Nnko S, Kuringe E, Nyato D, et al. Determinants of access to HIV testing and counselling services among female sex workers in sub-Saharan Africa: a systematic review. *BMC Public Health* 2019;19(1):15. doi: 10.1186/s12889-018-6362-0 [published Online First: 2019/01/07]
76. Pant Pai N, Sharma J, Shivkumar S, et al. Supervised and unsupervised self-testing for HIV in high- and low-risk populations: a systematic review. *PLoS Med* 2013;10(4):e1001414. doi: 10.1371/journal.pmed.1001414 [published Online First: 2013/04/09]
77. Pottie K, Medu O, Welch V, et al. Effect of rapid HIV testing on HIV incidence and services in populations at high risk for HIV exposure: an equity-focused systematic review. *BMJ Open* 2014;4(12):e006859. doi: 10.1136/bmjopen-2014-006859 [published Online First: 2014/12/17]
78. Qiao S, Zhou GY, Li XM. Disclosure of Same-Sex Behaviors to Health-care Providers and Uptake of HIV Testing for Men Who Have Sex With Men: A Systematic Review. *Am J Mens Health* 2018;12(5):1197-214. doi: 10.1177/1557988318784149
79. Qin YL, Han L, Babbitt A, et al. Experiences using and organizing HIV self-testing. *Aids* 2018;32(3):371-81. doi: 10.1097/qad.0000000000001705
80. Stannah J, Dale E, Elmes J, et al. HIV testing and engagement with the HIV treatment cascade among men who have sex with men in Africa: a systematic review and meta-analysis. *Lancet HIV* 2019;6(11):e769-e87. doi: 10.1016/s2352-3018(19)30239-5 [published Online First: 2019/10/12]
81. Stojanovski K, Naja-Riese G, King EJ, et al. A Systematic Review of the Social Network Strategy to Optimize HIV Testing in Key Populations to End the Epidemic in the United States. *AIDS Behav* 2021:1-19. doi: 10.1007/s10461-021-03259-z [published Online First: 2021/04/20]
82. Tan K, Black BP. A Systematic Review of Health Care Provider-Perceived Barriers and Facilitators to Routine HIV Testing in Primary Care Settings in the Southeastern United States. *J Assoc Nurses Aids Care* 2018;29(3):357-70. doi: 10.1016/j.jana.2017.12.006
83. Tokar A, Broerse JEW, Blanchard J, et al. HIV Testing and Counseling Among Female Sex Workers: A Systematic Literature Review. *AIDS Behav* 2018;22(8):2435-57. doi: 10.1007/s10461-018-2043-3 [published Online First: 2018/02/22]
84. Witzel TC, Lora W, Lees S, et al. Uptake contexts and perceived impacts of HIV testing and counselling among adults in East and Southern Africa: A meta-ethnographic review. *PLoS One* 2017;12(2):e0170588. doi: 10.1371/journal.pone.0170588 [published Online First: 2017/02/17]
85. Adeoye-Agboola DI, Evans H, Hewson D, et al. Factors influencing HIV disclosure among people living with HIV/AIDS in Nigeria: a systematic review using narrative synthesis and meta-analysis. *Public Health* 2016;136:13-28. doi: 10.1016/j.puhe.2016.02.021 [published Online First: 2016/04/10]
86. Azétsop J, Diop BA. Access to antiretroviral treatment, issues of well-being and public health governance in Chad: What justifies the limited success of the universal access policy? *Philosophy, Ethics, and Humanities in Medicine* 2013;8:14. doi: http://dx.doi.org/10.1186/1747-5341-8-8
87. Belay GM, Endalamaw A, Ayele AD. Late presentation of HIV positive adults and its predictors to HIV/AIDS care in Ethiopia: a systematic review and meta-analysis. *BMC Infect Dis* 2019;19(1):534. doi: 10.1186/s12879-019-4156-3 [published Online First: 2019/06/19]
88. Bharat S. A systematic review of HIV/AIDS-related stigma and discrimination in India: current understanding and future needs. *Sahara j* 2011;8(3):138-49. doi: 10.1080/17290376.2011.9724996 [published Online First: 2011/09/01]
89. Bradley E, Forsberg K, Betts JE, et al. Factors Affecting Pre-Exposure Prophylaxis Implementation for Women in the United States: A Systematic Review. *J Womens Health (Larchmt)* 2019;28(9):1272-85. doi: 10.1089/jwh.2018.7353 [published Online First: 2019/06/11]
90. Darlington CK, Hutson SP. Understanding HIV-related stigma among women in the Southern United States: A literature review. *AIDS and Behavior* 2017;21(1):12-26. doi: http://dx.doi.org/10.1007/s10461-016-1504-9
91. Das S, Leibowitz GS. Mental health needs of people living with HIV/AIDS in India: a literature review. *AIDS Care* 2011;23(4):417-25. doi: 10.1080/09540121.2010.507752 [published Online First: 2010/12/15]
92. Kerr ZY, Miller KR, Galos D, et al. Challenges, coping strategies, and recommendations related to the HIV services field in the HAART era: a systematic literature review of qualitative studies from the

For peer review only - http://bmjopen.bmj.com/site/about/guidelines.xhtml

United States and Canada. *AIDS Patient Care STDS* 2013;27(2):85-95. doi: 10.1089/apc.2012.0356 [published Online First: 2013/01/23]
93. Loutfy M, Tharao W, Logie C, et al. Systematic review of stigma reducing interventions for African/Black diasporic women. *J Int AIDS Soc* 2015;18(1):19835. doi: 10.7448/ias.18.1.19835 19835 [published Online First: 2015/04/12]
94. Mey A, Plummer D, Dukie S, et al. Motivations and Barriers to Treatment Uptake and Adherence Among People Living with HIV in Australia: A Mixed-Methods Systematic Review. *AIDS Behav* 2017;21(2):352-85. doi: 10.1007/s10461-016-1598-0 [published Online First: 2016/11/09]
95. Monteiro SS, Brigeiro M, Vilella WV, et al. Challenges facing HIV treatment as prevention in Brazil: an analysis drawing on literature on testing. *Cienc Saude Coletiva* 2019;24(5):1792-806. doi: 10.1590/1413-81232018245.16512017
96. Sangaramoorthy T, Jamison A, Dyer T. Older African Americans and the HIV Care Continuum: A Systematic Review of the Literature, 2003-2018. *AIDS Behav* 2019;23(4):973-83. doi: 10.1007/s10461-018-2354-4 [published Online First: 2018/12/07]
97. Vaitses Fontanari AM, Zanella GI, Feijó M, et al. HIV-related care for transgender people: A systematic review of studies from around the world. *Soc Sci Med* 2019;230:280-94. doi: 10.1016/j.socscimed.2019.03.016 [published Online First: 2019/04/30]
98. Weihs M, Meyer-Weitz A. Barriers to workplace HIV testing in South Africa: a systematic review of the literature. *AIDS Care* 2016;28(4):495-9. doi: 10.1080/09540121.2015.1109586 [published Online First: 2015/11/13]
99. Weldesenbet AB, Kebede SA, Tusa BS. The Effect of Poor Social Support on Depression among HIV/AIDS Patients in Ethiopia: A Systematic Review and Meta-Analysis. *Depress Res Treat* 2020;2020:6633686. doi: 10.1155/2020/6633686 [published Online First: 2021/01/26]
100. Whembolua GL, Conserve DF, Thomas K, et al. HIV serostatus disclosure in the Democratic Republic of the Congo: a systematic review. *AIDS Care* 2019;31(4):489-93. doi: 10.1080/09540121.2018.1510103 [published Online First: 2018/08/17]
101. Wirtz AL, Kirey A, Peryskina A, et al. Uncovering the epidemic of HIV among men who have sex with men in Central Asia. *Drug and Alcohol Dependence* 2013;132(Suppl 1):S17-S24. doi: http://dx.doi.org/10.1016/j.drugalcdep.2013.06.031
102. Young C. Understanding HIV-related posttraumatic stress disorder in South Africa: A review and conceptual framework. *African Journal of AIDS Research* 2011;10(2):139-48. doi: http://dx.doi.org/10.2989/16085906.2011.593376
103. Ammon N, Mason S, Corkery JM. Factors impacting antiretroviral therapy adherence among human immunodeficiency virus-positive adolescents in Sub-Saharan Africa: a systematic review. *Public Health* 2018;157:20-31. doi: 10.1016/j.puhe.2017.12.010 [published Online First: 2018/03/05]
104. Campbell L, Masquillier C, Thunnissen E, et al. Social and Structural Determinants of Household Support for ART Adherence in Low- and Middle-Income Countries: A Systematic Review *Int J Environ Res Public Health* 2020;17(11) doi: 10.3390/ijerph17113808 [published Online First: 2020/05/31]
105. Edeza A, Karina Santamaria E, Valente PK, et al. Experienced barriers to adherence to pre-exposure prophylaxis for HIV prevention among MSM: a systematic review and meta-ethnography of qualitative studies. *AIDS Care* 2020:1-9. doi: 10.1080/09540121.2020.1778628 [published Online First: 2020/06/13]
106. Geter A, Sutton MY, Hubbard McCree D. Social and structural determinants of HIV treatment and care among black women living with HIV infection: a systematic review: 2005-2016. *AIDS Care* 2018;30(4):409-16. doi: 10.1080/09540121.2018.1426827 [published Online First: 2018/01/30]
107. Heestermans T, Browne JL, Aitken SC, et al. Determinants of adherence to antiretroviral therapy among HIV-positive adults in sub-Saharan Africa: a systematic review. *BMJ Glob Health* 2016;1(4):e000125. doi: 10.1136/bmjgh-2016-000125 [published Online First: 2017/06/08]
108. Katz IT, Ryu AE, Onuegbu AG, et al. Impact of HIV-related stigma on treatment adherence: systematic review and meta-synthesis. *J Int AIDS Soc* 2013;16(3 Suppl 2):18640. doi: 10.7448/ias.16.3.18640
18640 [published Online First: 2013/11/28]
109. Knettel BA, Cichowitz C, Ngocho JS, et al. Retention in HIV Care During Pregnancy and the Postpartum Period in the Option B+ Era: Systematic Review and Meta-Analysis of Studies in Africa. *J Acquir Immune Defic Syndr* 2018;77(5):427-38. doi: 10.1097/qai.0000000000001616 [published Online First: 2017/12/30]

For peer review only

110. Lambert CC, Mugavero MJ, Najjar YS, et al. The State of Adherence to HIV Care in Black Women. *J Assoc Nurses AIDS Care* 2018;29(4):487-503. doi: 10.1016/j.jana.2018.02.008 [published Online First: 2018/05/17]
111. Makanjuola T, Taddese HB, Booth A. Factors associated with adherence to treatment with isoniazid for the prevention of tuberculosis amongst people living with HIV/AIDS: a systematic review of qualitative data. *PLoS One* 2014;9(2):e87166. doi: 10.1371/journal.pone.0087166 [published Online First: 2014/02/06]
112. Maxwell S, Gafos M, Shahmanesh M. Pre-exposure Prophylaxis Use and Medication Adherence Among Men Who Have Sex With Men: A Systematic Review of the Literature. *J Assoc Nurses AIDS Care* 2019;30(4):e38-e61. doi: 10.1097/jnc.0000000000000105 [published Online First: 2019/06/27]
113. Omonaiye O, Kusljic S, Nicholson P, et al. Medication adherence in pregnant women with human immunodeficiency virus receiving antiretroviral therapy in sub-Saharan Africa: a systematic review. *BMC Public Health* 2018;18(1):805. doi: 10.1186/s12889-018-5651-y [published Online First: 2018/06/28]
114. Paramesha AE, Chacko LK. Predictors of adherence to antiretroviral therapy among PLHIV. *Indian J Public Health* 2019;63(4):367-+. doi: 10.4103/ijph.IJPH_376_18
115. Shubber Z, Ford N. Adherence to HIV post-exposure prophylaxis for children/adolescents who have been sexually assaulted: A systematic review of barriers, enablers, and interventions. *Child Abuse Negl* 2019:104143. doi: 10.1016/j.chiabu.2019.104143 [published Online First: 2019/09/17]
116. Shubber Z, Mills EJ, Nachega JB, et al. Patient-Reported Barriers to Adherence to Antiretroviral Therapy: A Systematic Review and Meta-Analysis. *PLoS Med* 2016;13(11):e1002183. doi: 10.1371/journal.pmed.1002183 [published Online First: 2016/11/30]
117. Sidebottom D, Ekström AM, Strömdahl S. A systematic review of adherence to oral pre-exposure prophylaxis for HIV - how can we improve uptake and adherence? *BMC Infect Dis* 2018;18(1):581. doi: 10.1186/s12879-018-3463-4 [published Online First: 2018/11/18]
118. Sweeney SM, Vanable PA. The Association of HIV-Related Stigma to HIV Medication Adherence: A Systematic Review and Synthesis of the Literature. *AIDS Behav* 2016;20(1):29-50. doi: 10.1007/s10461-015-1164-1 [published Online First: 2015/08/26]
119. Wasti SP, van Teijlingen E, Simkhada P, et al. Factors influencing adherence to antiretroviral treatment in Asian developing countries: a systematic review. *Trop Med Int Health* 2012;17(1):71-81. doi: 10.1111/j.1365-3156.2011.02888.x [published Online First: 2011/10/05]
120. Zuniga JA, Wright C, Fordyce J, et al. Self-Management of HIV and Diabetes in African American Women: A Systematic Review of Qualitative Literature. *Diabetes Educ* 2018;44(5):419-34. doi: 10.1177/0145721718794879 [published Online First: 2018/08/28]
121. Arias-Colmenero T, Pérez-Morente M, Ramos-Morcillo AJ, et al. Experiences and Attitudes of People with HIV/AIDS: A Systematic Review of Qualitative Studies. *Int J Environ Res Public Health* 2020;17(2) doi: 10.3390/ijerph17020639 [published Online First: 2020/01/23]
122. Brömdal A, Mullens AB, Phillips TM, et al. Experiences of transgender prisoners and their knowledge, attitudes, and practices regarding sexual behaviors and HIV/STIs: A systematic review. *Int J Transgend* 2019;20(1):4-20. doi: 10.1080/15532739.2018.1538838 [published Online First: 2018/11/22]
123. Chi PL, Li XM. Impact of Parental HIV/AIDS on Children's Psychological Well-Being: A Systematic Review of Global Literature. *Aids and Behavior* 2013;17(7):2554-74. doi: 10.1007/s10461-012-0290-2
124. Eshun-Wilson I, Rohwer A, Hendricks L, et al. Being HIV positive and staying on antiretroviral therapy in Africa: A qualitative systematic review and theoretical model. *PLoS One* 2019;14(1):e0210408. doi: 10.1371/journal.pone.0210408 [published Online First: 2019/01/11]
125. Gannon BN, Stacciarini J-MR. Review of the literature: A rural–urban comparison of social networks of older adults living with HIV. *JANAC: Journal of the Association of Nurses in AIDS Care* 2016;27(4):419-29. doi: http://dx.doi.org/10.1016/j.jana.2016.02.004
126. Goldberg RE, Short SE. What do we know about children living with HIV-infected or AIDS-ill adults in Sub-Saharan Africa? A systematic review of the literature. *AIDS Care* 2016;28 Suppl 2(sup2):130-41. doi: 10.1080/09540121.2016.1176684 [published Online First: 2016/07/09]
127. Hainsworth EG, Shahmanesh M, Stevenson F. Exploring the views and experiences of HIV positive patients treated for cancer: a systematic review of the literature. *AIDS Care* 2018;30(5):535-43. doi: 10.1080/09540121.2017.1381332 [published Online First: 2017/10/05]

For peer review only - http://bmjopen.bmj.com/site/about/guidelines.xhtml

128. Kimera E, Vindevogel S, De Maeyer J, et al. Challenges and support for quality of life of youths living with HIV/AIDS in schools and larger community in East Africa: a systematic review. *Syst Rev* 2019;8(1):64. doi: 10.1186/s13643-019-0980-1 [published Online First: 2019/02/28]
129. Lowther K, Selman L, Harding R, et al. Experience of persistent psychological symptoms and perceived stigma among people with HIV on antiretroviral therapy (ART): a systematic review. *Int J Nurs Stud* 2014;51(8):1171-89. doi: 10.1016/j.ijnurstu.2014.01.015 [published Online First: 2014/03/08]
130. McInerney P, Brysiewicz P. A systematic review of the experiences of caregivers in providing home-based care to persons with HIV/AIDS in Africa. *JBI Libr Syst Rev* 2009;7(4):130-53. doi: 10.11124/01938924-200907040-00001 [published Online First: 2009/01/01]
131. Nattabi B, Li J, Thompson SC, et al. A systematic review of factors influencing fertility desires and intentions among people living with HIV/AIDS: implications for policy and service delivery. *AIDS Behav* 2009;13(5):949-68. doi: 10.1007/s10461-009-9537-y [published Online First: 2009/03/31]
132. Roger KS, Mignone J, Kirkland S. Social aspects of HIV/AIDS and aging: a thematic review. *Can J Aging* 2013;32(3):298-306. doi: 10.1017/s0714980813000330 [published Online First: 2013/08/15]
133. Rubtsova AA, Kempf MC, Taylor TN, et al. Healthy Aging in Older Women Living with HIV Infection: a Systematic Review of Psychosocial Factors. *Curr HIV/AIDS Rep* 2017;14(1):17-30. doi: 10.1007/s11904-017-0347-y [published Online First: 2017/02/15]
134. Zeglin RJ, Hergenrather KC, Garcia JH. A Systematic Review of the Common Experiences of Caregivers of HIV plus Men Who Have Sex with Men: Implications for Counselors. *J Rehabil* 2018;84(1):22-30.
135. Baral S, Holland CE, Shannon K, et al. Enhancing benefits or increasing harms: community responses for HIV among men who have sex with men, transgender women, female sex workers, and people who inject drugs. *J Acquir Immune Defic Syndr* 2014;66 Suppl 3:S319-28. doi: 10.1097/qai.0000000000000233 [published Online First: 2014/07/10]
136. Beres LK, Narasimhan M, Robinson J, et al. Non-specialist psychosocial support interventions for women living with HIV: A systematic review. *AIDS Care* 2017;29(9):1079-87. doi: 10.1080/09540121.2017.1317324 [published Online First: 2017/04/26]
137. Campbell C, Skovdal M, Gibbs A. Creating social spaces to tackle AIDS-related stigma: reviewing the role of church groups in Sub-Saharan Africa. *AIDS Behav* 2011;15(6):1204-19. doi: 10.1007/s10461-010-9766-0 [published Online First: 2010/07/30]
138. Han HR, Kim K, Murphy J, et al. Community health worker interventions to promote psychosocial outcomes among people living with HIV-A systematic review. *PLoS One* 2018;13(4):e0194928. doi: 10.1371/journal.pone.0194928 [published Online First: 2018/04/25]
139. Kaufman ZA, Spencer TS, Ross DA. Effectiveness of sport-based HIV prevention interventions: a systematic review of the evidence. *AIDS Behav* 2013;17(3):987-1001. doi: 10.1007/s10461-012-0348-1 [published Online First: 2012/10/26]
140. Kerrigan D, Kennedy CE, Morgan-Thomas R, et al. A community empowerment approach to the HIV response among sex workers: effectiveness, challenges, and considerations for implementation and scale-up. *Lancet* 2015;385(9963):172-85. doi: 10.1016/s0140-6736(14)60973-9 [published Online First: 2014/07/26]
141. Mak WWS, Mo PKH, Ma GYK, et al. Meta-analysis and systematic review of studies on the effectiveness of HIV stigma reduction programs. *Soc Sci Med* 2017;188:30-40. doi: 10.1016/j.socscimed.2017.06.045 [published Online First: 2017/07/14]
142. Rao D, Elshafei A, Nguyen M, et al. A systematic review of multi-level stigma interventions: state of the science and future directions. *BMC Med* 2019;17(1):41. doi: 10.1186/s12916-018-1244-y [published Online First: 2019/02/17]
143. Sengupta S, Banks B, Jonas D, et al. HIV interventions to reduce HIV/AIDS stigma: a systematic review. *AIDS Behav* 2011;15(6):1075-87. doi: 10.1007/s10461-010-9847-0 [published Online First: 2010/11/23]
144. Skevington SM, Sovetkina EC, Gillison FB. A systematic review to quantitatively evaluate 'Stepping Stones': a participatory community-based HIV/AIDS prevention intervention. *AIDS Behav* 2013;17(3):1025-39. doi: 10.1007/s10461-012-0327-6 [published Online First: 2012/11/07]
145. Stangl AL, Lloyd JK, Brady LM, et al. A systematic review of interventions to reduce HIV-related stigma and discrimination from 2002 to 2013: how far have we come? *J Int AIDS Soc* 2013;16(3 Suppl 2):18734. doi: 10.7448/ias.16.3.18734
18734 [published Online First: 2013/11/28]

For peer review only - http://bmjopen.bmj.com/site/about/guidelines.xhtml

146. Williams MV, Palar K, Derose KP. Congregation-based programs to address HIV/AIDS: elements of successful implementation. *J Urban Health* 2011;88(3):517-32. doi: 10.1007/s11524-010-9526-5 [published Online First: 2011/02/19]
147. Aderomilehin O, Hanciles-Amu A, Ozoya OO. Perspectives and Practice of HIV Disclosure to Children and Adolescents by Health-Care Providers and Caregivers in sub-Saharan Africa: A Systematic Review. *Front Public Health* 2016;4:166. doi: 10.3389/fpubh.2016.00166 [published Online First: 2016/08/30]
148. Cao WN, Wong HM, Chang C, et al. Behavioral interventions promoting HIV serostatus disclosure to sex partners among HIV-positive men who have sex with men: a systematic review. *Int J Public Health* 2019;64(7):985-98. doi: 10.1007/s00038-019-01275-4
149. Kennedy CE, Haberlen S, Amin A, et al. Safer disclosure of HIV serostatus for women living with HIV who experience or fear violence: a systematic review. *J Int AIDS Soc* 2015;18(Suppl 5):20292. doi: 10.7448/ias.18.6.20292
20292 [published Online First: 2015/12/09]
150. Krauss BJ, Letteney S, Okoro CN. Why Tell Children: A Synthesis of the Global Literature on Reasons for Disclosing or Not Disclosing an HIV Diagnosis to Children 12 and under. *Front Public Health* 2016;4:13. doi: 10.3389/fpubh.2016.00181
151. Qiao S, Li XM, Stanton B. Disclosure of Parental HIV Infection to Children: A Systematic Review of Global Literature. *Aids and Behavior* 2013;17(1):369-89. doi: 10.1007/s10461-011-0069-x
152. Tam M, Amzel A, Phelps BR. Disclosure of HIV serostatus among pregnant and postpartum women in sub-Saharan Africa: a systematic review. *AIDS Care* 2015;27(4):436-50. doi: 10.1080/09540121.2014.997662 [published Online First: 2015/01/31]
153. Vreeman RC, Gramelspacher AM, Gisore PO, et al. Disclosure of HIV status to children in resource-limited settings: a systematic review. *J Int AIDS Soc* 2013;16(1):18466. doi: 10.7448/ias.16.1.18466
18466 [published Online First: 2013/05/30]
154. Whembolua GL, Conserve DF, Thomas K, et al. A Systematic Review of HIV Serostatus Disclosure Among African Immigrants in Europe. *J Immigr Minor Health* 2017;19(4):947-58. doi: 10.1007/s10903-016-0456-5 [published Online First: 2016/07/09]
155. Wilkinson B, Arora KS. A Systematic Review on Confidentiality, Disclosure, and Stigma in the United States: Lessons for HIV Care in Pregnancy From Reproductive Genetics. *New Bioeth* 2015;21(2):142-54. doi: 10.1179/2050287715z.00000000067 [published Online First: 2015/01/01]
156. Cheema E, Abbas A, Al-Hamid A. Healthcare-related factors affecting the management of HIV infected patients: a systematic review of qualitative evidence. *Int J STD AIDS* 2019;30(14):1350-61. doi: 10.1177/0956462419875357 [published Online First: 2019/11/20]
157. Feyissa GT, Lockwood C, Woldie M, et al. Reducing HIV-related stigma and discrimination in healthcare settings: a systematic review of guidelines, tools, standards of practice, best practices, consensus statements and systematic reviews. *J Multidiscip Healthc* 2018;11:405-16. doi: 10.2147/jmdh.S170720 [published Online First: 2018/09/15]
158. Feyissa GT, Lockwood C, Woldie M, et al. Reducing HIV-related stigma and discrimination in healthcare settings: A systematic review of quantitative evidence. *PLoS One* 2019;14(1):e0211298. doi: 10.1371/journal.pone.0211298 [published Online First: 2019/01/27]
159. Geter A, Herron AR, Sutton MY. HIV-Related Stigma by Healthcare Providers in the United States: A Systematic Review. *AIDS Patient Care STDS* 2018;32(10):418-24. doi: 10.1089/apc.2018.0114 [published Online First: 2018/10/03]
160. Kaladharan S, Daken K, Mullens AB, et al. Tools to measure HIV knowledge, attitudes & practices (KAPs) in healthcare providers: a systematic review. *AIDS Care* 2020:1-7. doi: 10.1080/09540121.2020.1822502 [published Online First: 2020/09/24]
161. Nyblade L, Stockton MA, Giger K, et al. Stigma in health facilities: why it matters and how we can change it. *BMC Med* 2019;17(1):25. doi: 10.1186/s12916-019-1256-2 [published Online First: 2019/02/16]
162. Phillips JC, Caine V, Dewart G, et al. Teaching HIV-specific content for pre-licensure nursing and health professions students: a review and synthesis. *Aids Care-Psychol Socio-Med Asp Aids-Hiv* 2018;30(12):1614-21. doi: 10.1080/09540121.2018.1510108
163. Pleuhs B, Quinn KG, Walsh JL, et al. Health Care Provider Barriers to HIV Pre-Exposure Prophylaxis in the United States: A Systematic Review. *AIDS Patient Care STDS* 2020;34(3):111-23. doi: 10.1089/apc.2019.0189 [published Online First: 2020/02/29]

For peer review only

164. Anderson JE, Kanters S. Lack of Sexual Minorities' Rights as a Barrier to HIV Prevention Among Men Who Have Sex with Men and Transgender Women in Asia: A Systematic Review. *Lgbt Health* 2015;2(1):16-26. doi: 10.1089/lgbt.2014.0024
165. Juliastuti D, Dean J, Fitzgerald L. Sexual and reproductive health of women living with HIV in Muslim-majority countries: a systematic mixed studies review. *BMC Int Health Hum Rights* 2020;20(1):5. doi: 10.1186/s12914-020-00225-z [published Online First: 2020/03/13]
166. Jürgens R, Csete J, Amon JJ, et al. People who use drugs, HIV, and human rights. *The Lancet* 2010;376(9739):475-85. doi: http://dx.doi.org/10.1016/S0140-6736(10)60830-6
167. Malta M, Ralil da Costa M, Bastos FI. The paradigm of universal access to HIV-treatment and human rights violation: how do we treat HIV-positive people who use drugs? *Curr HIV/AIDS Rep* 2014;11(1):52-62. doi: 10.1007/s11904-013-0196-2 [published Online First: 2013/12/27]
168. Oldenburg CE, Perez-Brumer AG, Reisner SL, et al. Human rights protections and HIV prevalence among MSM who sell sex: Cross-country comparisons from a systematic review and meta-analysis. *Global Public Health* 2018;13(4):414-25. doi: 10.1080/17441692.2016.1149598
169. Stangl AL, Singh D, Windle M, et al. A systematic review of selected human rights programs to improve HIV-related outcomes from 2003 to 2015: what do we know? *BMC Infect Dis* 2019;19(1):209. doi: 10.1186/s12879-019-3692-1 [published Online First: 2019/03/06]
170. Wagener MN, Roelofs PDDM, Miedema HS, et al. The development of a multidisciplinary, evidence-based guideline for “HIV and employment”. *AIDS Care* 2015;27(2):133-41. doi: http://dx.doi.org/10.1080/09540121.2014.952612
171. Chambers LA, Wilson MG, Rueda S, et al. Evidence informing the intersection of HIV, aging and health: A scoping review. *AIDS and Behavior* 2014;18(4):661-75. doi: http://dx.doi.org/10.1007/s10461-013-0627-5
172. Ingram L, Stafford C, Deming ME, et al. A Systematic Mixed Studies Review of the Intersections of Social-Ecological Factors and HIV Stigma in People Living With HIV in the U.S. South. *J Assoc Nurses AIDS Care* 2019;30(3):330-43. doi: 10.1097/jnc.0000000000000076 [published Online First: 2019/04/26]
173. Jackson-Best F, Edwards N. Stigma and intersectionality: a systematic review of systematic reviews across HIV/AIDS, mental illness, and physical disability. *BMC Public Health* 2018;18(1):919. doi: 10.1186/s12889-018-5861-3 [published Online First: 2018/07/28]
174. Monteiro SS, Villela WV, Soares PS. The interaction between axes of inequality in studies on discrimination, stigma and HIV/AIDS: contributions to the recent international literature. *Glob Public Health* 2013;8(5):519-33. doi: 10.1080/17441692.2013.779738 [published Online First: 2013/04/05]
175. Smith SC. The intersection of race, military status, and disability: A systematic review of marginalization and self-esteem being a mediator against negative life outcomes for HIV positive African American male veterans [Psy.D.]. Azusa Pacific University, 2019.
176. Wilson PA, Valera P, Martos AJ, et al. Contributions of Qualitative Research in Informing HIV/AIDS Interventions Targeting Black MSM in the United States. *J Sex Res* 2016;53(6):642-54. doi: 10.1080/00224499.2015.1016139 [published Online First: 2015/08/05]
177. Alexandra Marshall S, Brewington KM, Kathryn Allison M, et al. Measuring HIV-related stigma among healthcare providers: a systematic review. *AIDS Care* 2017;29(11):1337-45. doi: 10.1080/09540121.2017.1338654 [published Online First: 2017/06/11]
178. Earnshaw VA, Chaudoir SR. From conceptualizing to measuring HIV stigma: a review of HIV stigma mechanism measures. *AIDS Behav* 2009;13(6):1160-77. doi: 10.1007/s10461-009-9593-3 [published Online First: 2009/07/29]
179. Fitzgerald-Husek A, Van Wert MJ, Ewing WF, et al. Measuring stigma affecting sex workers (SW) and men who have sex with men (MSM): A systematic review. *PLoS One* 2017;12(11):e0188393. doi: 10.1371/journal.pone.0188393 [published Online First: 2017/12/01]
180. Kipp AM, Haley C, Mukolo A, et al. GAPS IN MEASURING AND UNDERSTANDING THE EFFECT OF HIV STIGMA ON HEALTH OUTCOMES: INSIGHT FROM A SYSTEMATIC REVIEW OF HIV STIGMA AND ART ADHERENCE. *Ann Behav Med* 2012;43:S59-S59.
181. Mahajan AP, Sayles JN, Patel VA, et al. Stigma in the HIV/AIDS epidemic: A review of the literature and recommendations for the way forward. *AIDS* 2008;22(Suppl2):S67-S79. doi: http://dx.doi.org/10.1097/01.aids.0000327438.13291.62
182. McAteer CI, Truong NT, Aluoch J, et al. A systematic review of measures of HIV/AIDS stigma in paediatric HIV-infected and HIV-affected populations. *J Int AIDS Soc* 2016;19(1):21204. doi: 10.7448/ias.19.1.21204
21204 [published Online First: 2016/10/09]

For peer review only - http://bmjopen.bmj.com/site/about/guidelines.xhtml

183. Dunbar W, Labat A, Raccurt C, et al. A realist systematic review of stigma reduction interventions for HIV prevention and care continuum outcomes among men who have sex with men. *Int J STD AIDS* 2020;31(8):712-23. doi: 10.1177/0956462420924984 [published Online First: 2020/07/08]
184. Glick JL, Russo R, Jivapong B, et al. The PrEP Care Continuum Among Cisgender Women Who Sell Sex and/or Use Drugs Globally: A Systematic Review. *AIDS Behav* 2020;24(5):1312-33. doi: 10.1007/s10461-019-02733-z [published Online First: 2019/12/07]
185. Liu X, Erasmus V, Wu Q, et al. Behavioral and psychosocial interventions for HIV prevention in floating populations in China over the past decade: a systematic literature review and meta-analysis. *PLoS One* 2014;9(6):e101006. doi: 10.1371/journal.pone.0101006 [published Online First: 2014/06/26]
186. Poteat T, Wirtz AL, Radix A, et al. HIV risk and preventive interventions in transgender women sex workers. *The Lancet* 2015;385(9964):274-86. doi: http://dx.doi.org/10.1016/S0140-6736(14)60833-3
187. Chen D, Luo G, Meng X, et al. Efficacy of HIV interventions among factory workers in low- and middle-income countries: a systematic review. *BMC Public Health* 2020;20(1):1310. doi: 10.1186/s12889-020-09333-w [published Online First: 2020/08/30]
188. Hartog K, Hubbard CD, Krouwer AF, et al. Stigma reduction interventions for children and adolescents in low- and middle-income countries: Systematic review of intervention strategies. *Soc Sci Med* 2020;246:112749. doi: 10.1016/j.socscimed.2019.112749 [published Online First: 2020/01/25]
189. Kemp CG, Jarrett BA, Kwon CS, et al. Implementation science and stigma reduction interventions in low- and middle-income countries: a systematic review. *BMC Med* 2019;17(1):6. doi: 10.1186/s12916-018-1237-x [published Online First: 2019/02/16]
190. Lofgren SM, Nakasujja N, Boulware DR. Systematic Review of Interventions for Depression for People Living with HIV in Africa. *Aids and Behavior* 2018;22(1):1-8. doi: 10.1007/s10461-017-1906-3
191. Ma PHX, Chan ZCY, Loke AY. Self-Stigma Reduction Interventions for People Living with HIV/AIDS and Their Families: A Systematic Review. *AIDS Behav* 2019;23(3):707-41. doi: 10.1007/s10461-018-2304-1 [published Online First: 2018/10/10]
192. Pantelic M, Sprague L, Stangl AL. It's not "all in your head": critical knowledge gaps on internalized HIV stigma and a call for integrating social and structural conceptualizations. *BMC Infect Dis* 2019;19(1):210. doi: 10.1186/s12879-019-3704-1 [published Online First: 2019/03/06]
193. Joseph Davey D, West S, Umutoni V, et al. A Systematic Review of the Current Status of Safer Conception Strategies for HIV Affected Heterosexual Couples in Sub-Saharan Africa. *AIDS Behav* 2018;22(9):2916-46. doi: 10.1007/s10461-018-2170-x [published Online First: 2018/06/06]
194. Taggart T, Grewe ME, Conserve DF, et al. Social Media and HIV: A Systematic Review of Uses of Social Media in HIV Communication. *J Med Internet Res* 2015;17(11):e248. doi: 10.2196/jmir.4387 [published Online First: 2015/11/04]
195. International Center for Research on Women (ICRW), STRIVE. A global HIV stigma reduction framework adapted and implemented in five settings in India: Summary report, 2013.
196. Stangl A, Brady L, Fritz K. STRIVE Technical Brief: Measuring HIV stigma and discrimination. Washington, D.C.: International Center for Research on Women, 2012 (Updated in 2018):1-7.
197. UNAIDS. Key Populations [cited Protocol refs. Available from: https://www.unaids.org/en/topic/key-populations accessed January 12, 2021.
198. Global Network Of People Living with HIV (GNP+). The People Living with HIV Stigma Index [Available from: https://www.stigmaindex.org/ accessed February 22, 2021.
199. Mokkink LB, Terwee CB, Patrick DL, et al. The COSMIN checklist for assessing the methodological quality of studies on measurement properties of health status measurement instruments: an international Delphi study. *Qual Life Res* 2010;19(4):539-49. doi: 10.1007/s11136-010-9606-8 [published Online First: 2010/02/20]
200. Sterne JA, Hernan MA, Reeves BC, et al. ROBINS-I: a tool for assessing risk of bias in non-randomised studies of interventions. *BMJ* 2016;355:i4919. doi: 10.1136/bmj.i4919 [published Online First: 2016/10/14]
201. Cochrane Methods Bias. RoB 2: A revised Cochrane risk-of-bias tool for randomized trials [cited Protocol refs. Available from: https://methods.cochrane.org/bias/resources/rob-2-revised-cochrane-risk-bias-tool-randomized-trials accessed January 12, 2020.

For peer review only - http://bmjopen.bmj.com/site/about/guidelines.xhtml

202. Thomas J, O'Mara-Eves A, Brunton G. Using qualitative comparative analysis (QCA) in systematic reviews of complex interventions: a worked example. *Syst Rev* 2014;3:67. doi: 10.1186/2046-4053-3-67 [published Online First: 2014/06/22]
203. GRADE working group. GRADE [cited Protocol refs. Available from: https://www.gradeworkinggroup.org/ accessed January 12, 2020.

FUNDING STATEMENT
This work (Global Systematic Evidence Review: Getting to the Heart of Stigma) is supported by the International AIDS Society (IAS). The funder had no role in the development of the protocol or decision to submit this manuscript.

COMPETING INTERESTS STATEMENT
None of the authors have any conflicts of interest.

CONTRIBUTOR AND GUARANTOR INFORMATION
SH, LF, and SG developed the systematic review, all authors contributed to this protocol. SH serves as guarantor and accepts full responsibility for the work and the conduct of the study, had access to data, and controlled the decision to publish. The corresponding author attests that all listed authors meet authorship criteria and that no others meeting the criteria have been omitted.

COPYRIGHT/LICENSE FOR PUBLICATIION
The Corresponding Author has the right to grant on behalf of all authors and does grant on behalf of all authors, a worldwide licence to the Publishers and its licensees in perpetuity, in all forms, formats and media (whether known now or created in the future), to i) publish, reproduce, distribute, display and store the Contribution, ii) translate the Contribution into other languages, create adaptations, reprints, include within collections and create summaries, extracts and/or, abstracts of the Contribution, iii) create any other derivative work(s) based on the Contribution, iv) to exploit all subsidiary rights in the Contribution, v) the inclusion of electronic links from the Contribution to third party material where-ever it may be located; and, vi) licence any third party to do any or all of the above."

For peer review only

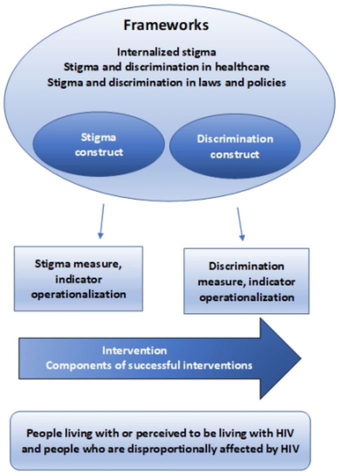

Analytic framework

Appendix

For peer review only

Draft search strategies

PubMed
(frameworks)
Date: 1/11/2021
Yield: 112
Terms
("framework"[All Fields] OR "framework s"[All Fields] OR "frameworks"[All Fields] OR "logic model"[All Fields] OR "analytic model"[All Fields]) AND ("stigma"[Title] OR "discrimination"[Title]) AND ("hiv"[MeSH Terms] OR "hiv"[All Fields] OR ("acquired immunodeficiency syndrome"[MeSH Terms] OR ("acquired"[All Fields] AND "immunodeficiency"[All Fields] AND "syndrome"[All Fields]) OR "acquired immunodeficiency syndrome"[All Fields] OR "aids"[All Fields]))
OR
(measures)
Date: 1/11/2021
Yield: 112
Filter: 2008-date
Terms
("measure*"[Title] OR "test"[Title] OR ("scale s"[All Fields] OR "scaled"[All Fields] OR "scaling"[All Fields] OR "scalings"[All Fields] OR "weights and measures"[MeSH Terms] OR ("weights"[All Fields] AND "measures"[All Fields]) OR "weights and measures"[All Fields] OR "scale"[All Fields] OR "scales"[All Fields]) OR "tool"[All Fields] OR "assess*"[Title] OR ("psychometrical"[All Fields] OR "psychometrically"[All Fields] OR "psychometrics"[MeSH Terms] OR "psychometrics"[All Fields] OR "psychometric"[All Fields])) AND ("stigma"[Title] OR "discrimination"[Title]) AND ("hiv"[MeSH Terms] OR "hiv"[All Fields] OR ("acquired immunodeficiency syndrome"[MeSH Terms] OR ("acquired"[All Fields] AND "immunodeficiency"[All Fields] AND "syndrome"[All Fields]) OR "acquired immunodeficiency syndrome"[All Fields] OR "aids"[All Fields]))
OR
(interventions SR)
Date: 1/11/2021
Filter: Systematic reviews
Yield: 225
Terms
("social stigma"[MeSH Terms] OR ("social"[All Fields] AND "stigma"[All Fields]) OR "social stigma"[All Fields] OR "stigma"[All Fields] OR "stigmas"[All Fields] OR "stigma s"[All Fields] OR ("discriminabilities"[All Fields] OR "discriminability"[All Fields] OR "discriminable"[All Fields] OR "discriminably"[All Fields] OR "discriminance"[All Fields] OR "discriminant"[All Fields] OR "discriminants"[All Fields] OR "discriminate"[All Fields] OR "discriminated"[All Fields] OR "discriminates"[All Fields] OR "discriminating"[All Fields] OR "discrimination, psychological"[MeSH Terms] OR ("discrimination"[All Fields] AND "psychological"[All Fields]) OR "psychological discrimination"[All Fields] OR "discrimination"[All Fields] OR "discriminations"[All Fields] OR "discriminative"[All Fields] OR "discriminatively"[All Fields] OR "discriminator"[All Fields] OR "discriminators"[All Fields])) AND ("hiv"[MeSH Terms] OR "hiv"[All Fields] OR ("acquired immunodeficiency syndrome"[MeSH Terms] OR

For peer review only - http://bmjopen.bmj.com/site/about/guidelines.xhtml

("acquired"[All Fields] AND "immunodeficiency"[All Fields] AND "syndrome"[All Fields]) OR "acquired immunodeficiency syndrome"[All Fields] OR "aids"[All Fields]))
OR
(interventions RCTs)
Date: 2/5/2021
Yield: 199 results
Filters: Randomized Controlled Trial, English language, 2008 - 2021
Terms
("social stigma"[MeSH Terms] OR ("social"[All Fields] AND "stigma"[All Fields]) OR "social stigma"[All Fields] OR "stigma"[All Fields] OR "stigmas"[All Fields] OR "stigma s"[All Fields] OR ("discriminabilities"[All Fields] OR "discriminability"[All Fields] OR "discriminable"[All Fields] OR "discriminably"[All Fields] OR "discriminance"[All Fields] OR "discriminant"[All Fields] OR "discriminants"[All Fields] OR "discriminate"[All Fields] OR "discriminated"[All Fields] OR "discriminates"[All Fields] OR "discriminating"[All Fields] OR "discrimination, psychological"[MeSH Terms] OR ("discrimination"[All Fields] AND "psychological"[All Fields]) OR "psychological discrimination"[All Fields] OR "discrimination"[All Fields] OR "discriminations"[All Fields] OR "discriminative"[All Fields] OR "discriminatively"[All Fields] OR "discriminator"[All Fields] OR "discriminators"[All Fields]))
AND
("hiv"[MeSH Terms] OR "hiv"[All Fields] OR ("acquired immunodeficiency syndrome"[MeSH Terms] OR ("acquired"[All Fields] AND "immunodeficiency"[All Fields] AND "syndrome"[All Fields]) OR "acquired immunodeficiency syndrome"[All Fields] OR "aids"[All Fields]))
OR
(systematic reviews)
Date: 5/6/2021
Yield: 235
Publication Date Range: 2008-05/06/2021
Filter: Systematic Review
Terms
Social stigma[MESH terms] OR stigma OR discrimination OR discrimination, psychological[MESH terms]
AND
HIV[Mesh terms] OR hiv OR acquired immunodeficiency syndrome[MESH terms] OR "acquired immunodeficiency syndrome" OR "AIDS"[Title/Abstract]

PsycINFO
(Frameworks; limited 'framework' terms to title field, 2008-2021 publication date range)
Date: 3/26/2021
Yield: 41
Terms
(ti(stigma OR discrimin*) OR ab(stigma OR discrimin*) OR su(stigma OR discrimin*)) AND (ti(HIV OR "acquired immunodeficiency syndrome") OR ab(HIV OR "acquired immunodeficiency syndrome") OR su(HIV OR "acquired immunodeficiency syndrome")) AND ti(framework OR frameworks OR "logic model" OR "analytic model") AND la.exact("ENG")
OR
(Measures; limited 'measures' terms to title field)

For peer review only - http://bmjopen.bmj.com/site/about/guidelines.xhtml

Date: 3/26/2021
Yield: 116
Terms
(ti(stigma OR discrimin*) OR ab(stigma OR discrimin*) OR su(stigma OR discrimin*)) AND (ti(HIV OR "acquired immunodeficiency syndrome") OR ab(HIV OR "acquired immunodeficiency syndrome") OR su(HIV OR "acquired immunodeficiency syndrome")) AND ti(measure OR measures OR scale OR scales OR tool OR tools) AND (la.exact("ENG") AND pd(20080101-20211231))
OR
Date: 3/19/2021
Yield: 58
Terms
((ti(stigma OR discrimin*) OR ab(stigma OR discrimin*) OR su(stigma OR discrimin*)) AND (ti(HIV OR "acquired immunodeficiency syndrome") OR ab(HIV OR "acquired immunodeficiency syndrome") OR su(HIV OR "acquired immunodeficiency syndrome")))
AND
(la.exact("ENG") AND me.exact("Prospective Study" OR "Clinical Trial")
AND
pd(20080101-20211231))
OR
Date: 5/6/2021
Yield: 100
Publication Date Range: After January 1, 2008
Methodology: Systematic Review
Terms
noft(stigma OR discriminat*) AND noft("hiv" OR "acquired immunodeficiency syndrome" OR "AIDS")

Web of Science
(Framework; omitted conference proceedings, added Research Areas, limited ‘framework’ terms to title field, 2008-2021 publication date range)
Yield: 14
Terms
(TS=(stigma OR discrim*) AND TS=(HIV OR "acquired immunodeficiency syndrome") AND TI=(framework OR frameworks OR "logic model" OR "logic models" OR "analytic model" OR "analytic models")) AND LANGUAGE: (English)
Refined by: RESEARCH AREAS: (HEALTH CARE SCIENCES SERVICES OR PSYCHOLOGY)
Indexes=SCI-EXPANDED, SSCI, BKCI-S, BKCI-SSH, ESCI Timespan=1900-2021
OR
(Measures; omitted conference proceedings, added Research Areas, limited ‘measures’ terms to title field)
Yield: 75
TS=(stigma OR discrim*) AND TS=(HIV OR "acquired immunodeficiency syndrome") AND TI=(measure OR measures OR scale OR scales OR tool OR tools)) AND LANGUAGE: (English)

For peer review only - http://bmjopen.bmj.com/site/about/guidelines.xhtml

Refined by: RESEARCH AREAS: (PSYCHOLOGY OR HEALTH CARE SCIENCES SERVICES)
Indexes=SCI-EXPANDED, SSCI, BKCI-S, BKCI-SSH, ESCI Timespan=2008-2021
OR
Interventions (omitted conference proceedings, added Research Areas, limited ‘RCT’ terms to topic field)
Yield: 76
(TS=(stigma OR discrim*) AND TS=(HIV OR "acquired immunodeficiency syndrome") AND TS=("randomized controlled trial" OR "randomized controlled trials" OR RCT OR RCTs OR "intervention study" OR "intervention studies")) AND LANGUAGE: (English)
Refined by: RESEARCH AREAS: (PSYCHOLOGY OR HEALTH CARE SCIENCES SERVICES)
Indexes=SCI-EXPANDED, SSCI, BKCI-S, BKCI-SSH, ESCI Timespan=2008-2021
OR
Date: 5/6/2021
Yield: 263
Publication Date Range: 2008-2021
Terms
Indexes=SCI-EXPANDED, SSCI, A&HCI, CPCI-S, CPCI-SSH, BKCI-S, BKCI-SSH, ESCI, CCR-EXPANDED
Search Strategy
TOPIC: (stigma OR discriminat*) AND TOPIC: ("hiv" OR "acquired immunodeficiency syndrome" OR "AIDS") AND TOPIC: ("systematic review")

Campbell Collaboration
Date: 1/19/2021
Yield: 81
Campbell Systematic Reviews
Terms
stigma* OR discriminat*" and "HIV OR AIDS OR "acquired immunodeficiency"
Research evidence by topic
(stigma OR discrimination)
(HIV OR AIDS)

Cochrane Database of Systematic Reviews
Date: 1/29/2021
Yield: 6
Terms
(stigma OR discrimination):ti,ab,kw (Word variations have been searched)
AND
MeSH descriptor: [HIV] explode all trees OR MeSH descriptor: [Acquired Immunodeficiency Syndrome] 5 tree(s) exploded OR HIV OR "acquired immunodeficiency syndrome" OR "acquired immunodeficiency syndrome"):ti,ab,kw (Word variations have been searched)

PROSPERO
Date: 1/19/2021

For peer review only - http://bmjopen.bmj.com/site/about/guidelines.xhtml

Yield: 173
Terms
(stigma or discrimin*)
AND
MeSH DESCRIPTOR HIV EXPLODE ALL TREES OR MeSH DESCRIPTOR Acquired Immunodeficiency Syndrome EXPLODE ALL OR "acquired immunodeficiency syndrome" OR "acquired immunodeficiency" OR HIV

Open Science Framework
Date: 1/27/2021
Yield: 4 relevant projects
Terms: (stigma OR discrimination) AND (HIV OR AIDS)

Universal Human Rights Index
Date: 1/27/2021
Platform: https://www.ohchr.org/EN/Issues/HIV/Pages/Documents.aspx
Yield: 2 relevant documents
Terms: “Persons living with HIV/AIDS” AND (stigma OR discrimination)

PAIS
Date: 4/9/2021
Yield: 9
Terms: NOFT(hive and stigma and law and intervention)

International AIDS Society (IAS)
Date: 1/27/2021
Yield: 5 relevant documents
Terms: (stigma OR discrimination) AND (HIV OR AIDS)

Joint United Nations Programme on HIV/AIDS (UNAIDS)
Date: 1/27/2021
Yield: 15 relevant documents
Terms: (stigma OR discrimination) AND (HIV OR AIDS)

United Nations Development Programme (UNDP)
Date: 4/13/2021
Yield: 6 relevant documents
Terms: (stigma OR discrimination) AND (HIV OR AIDS)

STRIVE:
Date: 1/27/2021
Yield: 2 relevant documents
Terms: (stigma OR discrimination) AND (HIV OR AIDS)

Health Policy Plus
Date: 1/27/2021

For peer review only - http://bmjopen.bmj.com/site/about/guidelines.xhtml

For peer review only

Yield: 5 relevant documents
Terms: (stigma OR discrimination) AND (HIV OR AIDS)

The Center for HIV Law and Policy
https://www.hivlawandpolicy.org/
Date: 4/19/2021
Yield: 15 relevant documents
Terms: topic: “stigma”

The Global Fund
https://www.theglobalfund.org/en/funding-model/throughout-the-cycle/community-rights-gender/
Date: 4/13/2021
Yield: 20 relevant documents
Terms: “Human rights”

HIV Legal Network
http://www.hivlegalnetwork.ca/site/?lang=en
Date: 4/16/2021
Publications/Find Publications/ keyword search:
Yield: 11 relevant documents
Terms: stigma

Sage: A resource-sharing community for Canadian HIV and hepatitis C service providers
http://sagecollection.ca/en
Limited publication type: Reports
Date: 4/16/2021
Yield: 3 potentially relevant documents
Terms: STIGMA AND (LAW OR LEGAL)

HeinOnline
Date: 3/19/2021
Yield: 272
Terms
(stigma OR discrmin*) AND (HIV OR "acquired immunodeficiency syndrome") AND (framework OR frameworks OR "logic model" OR "logic models" OR "analytic model" OR "analytic models") Topic: AIDS AND Timespan: 2008-2021
OR (stigma OR discrmin*) AND (HIV OR "acquired immunodeficiency syndrome") AND (measure OR measures OR scale OR scales OR asses* OR tool OR tools) AND Topic: AIDS AND Timespan: 2008-2021
OR
(stigma OR discrmin*) AND (HIV OR "acquired immunodeficiency syndrome") AND ("randomized controlled trial" OR "randomized controlled trials" OR "RCT" OR "RCTs") Topic: AIDS AND Timespan: 2008-2021
OR

For peer review only - http://bmjopen.bmj.com/site/about/guidelines.xhtml

(stigma OR discrmin*) AND (HIV OR "acquired immunodeficiency syndrome") AND ("systematic review" OR "systematic reviews") Topic: AIDS AND Timespan: 2008-2021

LegalTrac
Date: 5/12/2021
Yield: 78
Terms
keyword search - ("acquired immunodeficiency syndrome" OR HIV) AND (stigma OR discriminat*)
AND
Publication Date Range: 2008 - present
AND
Document Type: Article

For peer review only

For peer review only - http://bmjopen.bmj.com/site/about/guidelines.xhtml

Draft Evidence Table KQ1 Stigma and Discrimination Framework Descriptions

Framework title<br>Author group / publisher	Scope and Aim/Purpose<br>Target	Stigma subtype, definition<br>Discrimination subtype, definition	Components	Summary
Author, year<br>**Framework name:**<br>**Framework addressing:**<br>**Subdomain:**<br>**Specific to HIV:**	**Scope:**<br>**Aim/Purpose:**<br>**Target:**<br>**Framework HIV target:**	**Subtype:**<br>**Definitions:**		
Author, year<br>**Framework name:**<br>**Framework addressing:**<br>**Subdomain:**<br>**Specific to HIV:**	**Scope:**<br>**Aim/Purpose:**<br>**Target:**<br>**Framework HIV target:**	**Subtype:**<br>**Definitions:**		

Draft Evidence Table KQ2 Stigma and Discrimination Measure Descriptions

Author group, publication year<br>Measure name	Stigma / discrimination subtype<br>Definition<br>Framework	Target population<br>Surveyed participants	Scale structure<br>Number of items<br>Answer mode	Reliability<br>Validity
Author, year<br>**Author group/publisher:**<br>**Measure name:**<br>**Subdomain:**<br>**Specific to HIV:**	**Subtype**<br>**Definition:**<br>**Framework:**	**Target population:**<br>**Surveyed participants:**	**Scale structure:**<br>**Number of items:**<br>**Answer mode:**	**Reliability:**<br>**Validity**:
Author, year<br>**Author group/publisher:**<br>**Measure name:**<br>**Subdomain:**<br>**Specific to HIV:**	**Subtype**<br>**Definition:**<br>**Framework:**	**Target population:**<br>**Surveyed participants:**	**Scale structure:**<br>**Number of items:**<br>**Answer mode:**	**Reliability:**<br>**Validity**:

For peer review only

Draft Evidence Table KQ3 Stigma and Discrimination Intervention Evaluations

Study ID, PY<br>Country<br>Study design<br>Sample size	Participant details<br>Context / setting	Intervention type<br>Intervention description	Measure and effectiveness summary<br>Measure and unintended consequences
Author, year<br>**Country:**<br>**Study design:**<br>**Sample size:**<br>**Subdomain:**<br>**Specific to HIV:**	**Participant details:**<br>**Context / setting:**	**Intervention type:**<br>**Intervention description:**	**Measure and effectiveness summary:**<br>**Measure and unintended consequences:**

For peer review only

Draft Component Table KQ4a Stigma and Discrimination Intervention 'Critical Factors for Success'

Type of Intervention	Context	Component 1	Component 2	Component 3	Component 4	Component 5	Component 6	Component 7	Component 8
Intervention 1				√			√	√	√
Intervention 2			√				√		
Intervention 3		√				√	√		

Draft Component Table KQ4b Stigma and Discrimination Intervention 'Critical Factors for Failure'

Type of Intervention	Context	Component 1	Component 2	Component 3	Component 4	Component 5	Component 6	Component 7	Component 8
Intervention 1				√			√	√	√
Intervention 2			√				√		
Intervention 3		√				√	√		

For peer review only

PRISMA-P (Preferred Reporting Items for Systematic review and Meta-Analysis Protocols) 2015 checklist: recommended items to address in a systematic review protocol*

Section and topic	Page number	Item No	Checklist item
**ADMINISTRATIVE INFORMATION**			
Title:	1		
Identification	1	1a	Identify the report as a protocol of a systematic review
Update	N/A	1b	If the protocol is for an update of a previous systematic review, identify as such
Registration	2	2	If registered, provide the name of the registry (such as PROSPERO) and registration number
Authors:	1		
Contact	1	3a	Provide name, institutional affiliation, e-mail address of all protocol authors; provide physical mailing address of corresponding author
Contributions	20	3b	Describe contributions of protocol authors and identify the guarantor of the review
Amendments	N/A	4	If the protocol represents an amendment of a previously completed or published protocol, identify as such and list changes; otherwise, state plan for documenting important protocol amendments
Support:	20		
Sources	20	5a	Indicate sources of financial or other support for the review
Sponsor	20	5b	Provide name for the review funder and/or sponsor
Role of sponsor or funder	20	5c	Describe roles of funder(s), sponsor(s), and/or institution(s), if any, in developing the protocol
**INTRODUCTION**			
Rationale	3	6	Describe the rationale for the review in the context of what is already known
Objectives	3	7	Provide an explicit statement of the question(s) the review will address with reference to participants, interventions, comparators, and outcomes (PICO)
**METHODS**			
Eligibility criteria	5	8	Specify the study characteristics (such as PICO, study design, setting, time frame) and report characteristics (such as years considered, language, publication status) to be used as criteria for eligibility for the review
Information sources	5	9	Describe all intended information sources (such as electronic databases, contact with study authors, trial registers or other grey literature sources) with planned dates of coverage
Search strategy	Appendix	10	Present draft of search strategy to be used for at least one electronic database, including planned limits, such that it could be repeated
Study records:			

For peer review only

Data management	5	11a	Describe the mechanism(s) that will be used to manage records and data throughout the review
Selection process	5	11b	State the process that will be used for selecting studies (such as two independent reviewers) through each phase of the review (that is, screening, eligibility and inclusion in meta-analysis)
Data collection process	6	11c	Describe planned method of extracting data from reports (such as piloting forms, done independently, in duplicate), any processes for obtaining and confirming data from investigators
Data items	6	12	List and define all variables for which data will be sought (such as PICO items, funding sources), any pre-planned data assumptions and simplifications
Outcomes and prioritization	6	13	List and define all outcomes for which data will be sought, including prioritization of main and additional outcomes, with rationale
Risk of bias in individual studies	6-7	14	Describe anticipated methods for assessing risk of bias of individual studies, including whether this will be done at the outcome or study level, or both; state how this information will be used in data synthesis
Data synthesis	7-8	15a	Describe criteria under which study data will be quantitatively synthesised
	8	15b	If data are appropriate for quantitative synthesis, describe planned summary measures, methods of handling data and methods of combining data from studies, including any planned exploration of consistency (such as I^2, Kendall's τ)
	8	15c	Describe any proposed additional analyses (such as sensitivity or subgroup analyses, meta-regression)
	8	15d	If quantitative synthesis is not appropriate, describe the type of summary planned
Meta-bias(es)	7	16	Specify any planned assessment of meta-bias(es) (such as publication bias across studies, selective reporting within studies)
Confidence in cumulative evidence	7	17	Describe how the strength of the body of evidence will be assessed (such as GRADE)

*** It is strongly recommended that this checklist be read in conjunction with the PRISMA-P Explanation and Elaboration (cite when available) for important clarification on the items. Amendments to a review protocol should be tracked and dated. The copyright for PRISMA-P (including checklist) is held by the PRISMA-P Group and is distributed under a Creative Commons Attribution Licence 4.0.**

From: Shamseer L, Moher D, Clarke M, Ghersi D, Liberati A, Petticrew M, Shekelle P, Stewart L, PRISMA-P Group. Preferred reporting items for systematic review and meta-analysis protocols (PRISMA-P) 2015: elaboration and explanation. BMJ. 2015 Jan 2;349(jan02 1):g7647.

For peer review only

BMJ Open

Frameworks, measures, and interventions for HIV-related internalized stigma and stigma in healthcare and laws and policies: Systematic Review Protocol

Journal:	*BMJ Open*
Manuscript ID	bmjopen-2021-053608.R1
Article Type:	Protocol
Date Submitted by the Author:	08-Sep-2021
Complete List of Authors:	Hempel, Susanne; University of Southern California, Southern California Evidence Review Center<br>Ferguson, Laura; University of Southern California, Institute on Inequalities in Global Health<br>Bolshakova, Maria; University of Southern California, Southern California Evidence Review Center<br>Yagyu, Sachi; University of Southern California, Southern California Evidence Review Center<br>Fu, Ning; Shanghai University of Finance and Economics<br>Motala, Aneesa; University of Southern California, Southern California Evidence Review Center<br>Gruskin, Sofia; University of Southern California, Institute on Inequalities in Global Health
**Primary Subject Heading**:	HIV/AIDS
Secondary Subject Heading:	Health services research
Keywords:	HIV & AIDS < INFECTIOUS DISEASES, HEALTH SERVICES ADMINISTRATION & MANAGEMENT, ETHICS (see Medical Ethics)

SCHOLARONE™
Manuscripts

I, the Submitting Author has the right to grant and does grant on behalf of all authors of the Work (as defined in the below author licence), an exclusive licence and/or a non-exclusive licence for contributions from authors who are: i) UK Crown employees; ii) where BMJ has agreed a CC-BY licence shall apply, and/or iii) in accordance with the terms applicable for US Federal Government officers or employees acting as part of their official duties; on a worldwide, perpetual, irrevocable, royalty-free basis to BMJ Publishing Group Ltd ("BMJ") its licensees and where the relevant Journal is co-owned by BMJ to the co-owners of the Journal, to publish the Work in this journal and any other BMJ products and to exploit all rights, as set out in our licence.

The Submitting Author accepts and understands that any supply made under these terms is made by BMJ to the Submitting Author unless you are acting as an employee on behalf of your employer or a postgraduate student of an affiliated institution which is paying any applicable article publishing charge ("APC") for Open Access articles. Where the Submitting Author wishes to make the Work available on an Open Access basis (and intends to pay the relevant APC), the terms of reuse of such Open Access shall be governed by a Creative Commons licence – details of these licences and which Creative Commons licence will apply to this Work are set out in our licence referred to above.

Other than as permitted in any relevant BMJ Author's Self Archiving Policies, I confirm this Work has not been accepted for publication elsewhere, is not being considered for publication elsewhere and does not duplicate material already published. I confirm all authors consent to publication of this Work and authorise the granting of this licence.

For peer review only

Frameworks, measures, and interventions for HIV-related internalized stigma and stigma in healthcare and laws and policies: Systematic Review Protocol

Susanne Hempel, Southern California Evidence Review Center, Preventive Medicine, University of Southern California, Los Angeles, USA; ORCID ID 0000-0003-1597-5110; Susanne.Hempel@med.usc.edu (corresponding author)

Laura Ferguson, Institute on Inequalities in Global Health, University of Southern California, Los Angeles, USA

Maria Bolshakova, Southern California Evidence Review Center, Preventive Medicine, University of Southern California, Los Angeles, USA

Sachi Yagyu, Southern California Evidence Review Center, Preventive Medicine, University of Southern California, Los Angeles, USA

Ning Fu, School of Economics, Shanghai University of Finance and Economics, Shanghai, China

Aneesa Motala, Southern California Evidence Review Center, Preventive Medicine, University of Southern California, Los Angeles, USA

Sofia Gruskin, Institute on Inequalities in Global Health, University of Southern California, Los Angeles, USA

Word count: 4,096 (4,000 max)

For peer review only

ABSTRACT

Introduction: There is strong global commitment to eliminate HIV-related stigma. Wide variation exists in frameworks and measures, and many strategies to prevent, reduce, or mitigate stigma have been proposed but critical factors determining success or failure remain elusive.

Methods and analysis: Building on existing knowledge syntheses, we designed a systematic review to identify frameworks, measures, and intervention evaluations aiming to address internalized stigma, stigma and discrimination in healthcare, and stigma and discrimination at the legal or policy level. The review addresses four key questions (KQ): KQ1: Which conceptual frameworks have been proposed to assess internal stigma, stigma and discrimination experienced in healthcare settings, and stigma and discrimination entrenched in national laws and policies? KQ2: Which measures of stigma have been proposed and what are their descriptive properties? KQ3: Which interventions have been evaluated that aimed to reduce these types of stigma and discrimination or mitigate their adverse effects and what are the effectiveness and unintended consequences? KQ4: What common 'critical factors for success or failure' can be identified across interventions that have been evaluated? We will search PubMed, PsycINFO, Web of Science, Universal Human Rights Index, HeinOnline, PAIS, HIV Legal Network, CDSR, Campbell Collaboration, PROSPERO, and Open Science Framework. Critical appraisal will assess the source, processes, and consensus finding for frameworks; COSMIN criteria for measures; and risk of bias for interventions. Quality of evidence grading will apply GRADE. A gap analysis will provide targeted recommendations for future research. We will establish a compendium of frameworks, a comprehensive catalogue of available measures, and a synthesis of intervention characteristics to advance the science of HIV-related stigma.

Ethics and dissemination: The is registered in PROSPERO (CRD42021249348), the protocol incorporates stakeholder input, the results will be published in a journal manuscript, and the data will be made available in the Systematic Review Data Repository.

300 (max 300)

ARTICLE SUMMARY

Strengths and limitations of this study

- Extensive literature searches will summarize HIV stigma evidence.
- This review will establish a comprehensive compendium of frameworks, a user-friendly catalogue of existing measures, and a clear synthesis of the effects of interventions.
- The review is limited to internalized stigma, stigma and discrimination in healthcare, and stigma and discrimination at the legal or policy level.

KEY WORDS

AIDS, HIV, Systematic Review, Protocol

For peer review only

INTRODUCTION

Stigma is derived from a Greek word meaning a mark or stain. Much work around HIV-related stigma uses as its starting point Goffman's 1963 definition as "an attribute that is deeply discrediting".[1] This has been furthered by Hatzenbuehler and colleagues who, bringing in the idea of the power dynamics that allow the distancing or othering so often associated with stigma, define stigma as "the co-occurrence of labeling, stereotyping, separation, status loss and discrimination in a context in which power is exercised".[2] Relf and colleagues define stigma as "a social phenomenon that occurs when a person is evaluated as having an undesirable trait, attribute or behavior and is subsequently deemed imperfect by societal standards".[3] Each of these definitions brings in something slightly different and many of the core concepts are neatly summed up by Parker and Aggleton who note that "stigma functions at the intersection of culture, power and difference".[4] Stigma can be described as a dynamic process of devaluation that significantly discredits an individual in the eyes of others, such as when certain attributes are seized upon within particular cultures or settings and defined as discreditable or unworthy.

The term stigma is often used in the literature to encompass both stigma and discrimination even as these are conceptually distinct. While stigma refers to an attitude or belief, discrimination is the behavior or action that results from those attitudes or beliefs. Hence, when stigma is acted upon, the result can be discrimination. Discrimination may refer to any form of arbitrary distinction, exclusion or restriction affecting a person, usually (but not only) because of an inherent personal characteristic or perceived membership of a particular group.

HIV-related stigma has been defined by the Joint United Nations Programme on HIV/AIDS (UNAIDS) as negative beliefs, feelings and attitudes towards people living with HIV, groups associated with people living with HIV (e.g. families of people living with HIV), and other key populations at higher risk of HIV infection, such as people who inject drugs, sex workers, men who have sex with men, and transgender people.[5] Although there is no universal consensus as to their categorization, and many perceive these to be along a continuum, different domains have been identified within HIV-related stigma, including internalized, anticipated, perceived, enacted, externalized, and structural stigma. Different types of stigmas can be experienced and assessed both alone and in combination, each of which is experienced differently and therefore must be addressed differently. Discrimination, as defined under international human rights law, is any distinction, exclusion or restriction based indirectly or directly on grounds prohibited under international law, which has the effect or intent of nullifying the recognition, enjoyment or exercise on an equal basis of others of all human rights and fundamental freedoms, in the political, economic, social, cultural, civil or any other field.[6] In the case of HIV, this can be discrimination based on a person's real or perceived HIV-positive status, irrespective of whether or not there is any justification for these measures.[7] It can rise to the level of a human rights violation. HIV-related discrimination is therefore any distinction, exclusion or restriction (sometimes referred to as acts or omissions) based indirectly or directly on a person's real or perceived HIV status.[8]

There is strong global commitment to eliminate HIV-related stigma, starting with global political commitments and reflected in global and national strategies as well as the multitude of organizations and collaborations working to address stigma.[9 10] Yet, learning across interventions designed to mitigate against the experience and harmful impacts of stigma is limited by the multitude of evaluation frameworks and measures in use to assess the different dimensions of stigma. For example, a recent review of interventions to address self-stigma did not include a formal meta-analysis due to the heterogeneity of measures used, with eight different scales used across 20 studies.[7] A 2015 UNAIDS report documented over 60 tools to assess and/or address stigma and discrimination within healthcare settings.[11] Assessment of stigma and discrimination entrenched in laws and policies also takes multiple forms including the People Living with HIV Stigma Index, Module 6 of the Demographic and Health Survey, the Integrated Bio-Behavioural Survey, legal environment assessments, the Global Fund baseline assessment methodologies, and the National Commitments and Policy Instrument of the Global AIDS Monitoring process.

Understanding the state of the research in relation to measurement of self-stigma, in accessing health services, and in laws and policies, is needed to help inform future efforts, at all levels, to better address stigma and support people's health and well-being. This review will systematically identify and assess frameworks, measures and interventions of HIV-related individual internalized stigma, both stigma and discrimination within healthcare settings, and stigma and discrimination at the legal and policy level. The intersectionality of HIV-related stigma with other forms

For peer review only - http://bmjopen.bmj.com/site/about/guidelines.xhtml

of stigma such as stigma relating to 'key' and 'vulnerable' populations that are disproportionately affected by HIV is critical. The review will focus on HIV-related stigma itself, acknowledging as possible where intersectionality is addressed and, specifically, if there appear to be particular gaps in attention to stigma with respect to specific population groups.

Review questions

The systematic review will be guided by four Key Questions:

- Key Question 1: Which conceptual frameworks have been proposed to assess internal stigma, stigma and discrimination experienced in healthcare settings, and stigma and discrimination entrenched in national laws and policies?
- Key Question 2: Which measures (e.g. assessment scales) of these different types of stigma and discrimination have been proposed and what are their descriptive properties?
- Key Question 3: Which interventions have been evaluated that aimed to reduce these types of stigma and discrimination or mitigate their adverse effects and what are the effectiveness and unintended consequences of the interventions?
- Key Question 4: What common 'critical factors for success or failure' can be identified across the interventions that have been evaluated that might inform future interventions?

METHODS AND ANALYSIS

The systematic review will follow this protocol, the review is registered in PROSPERO (CRD42021249348), and the reporting of the protocol and the review will follow the PRISMA guidelines. The systematic review is part of a larger project undertaken by the International AIDS Society (IAS). It is accompanied by four national initiatives to explore stigma and discrimination in the local contexts of Kenya, Malawi, South Africa, and Zambia through key informant interviews and grey literature searches. The project started in November 2020 and is planned to be completed by March 2022.

The systematic review will follow a transparent and rigorous procedure to minimize review selection and reporting bias. We will search multiple disciplinary and interdisciplinary sources to ensure all relevant studies are captured. Citations and full text publications will be screened by independent literature reviewers to reduce reviewer errors and bias. Eligibility decisions, including reasons for exclusions, will be tracked in citation management software. Data abstraction and critical appraisal will be conducted in online software designed for systematic reviews using detailed, pilot-tested forms with clear reviewer instructions to avoid ambiguity and ensure replicability of coding decisions. The collected data will be accessible in a review data repository.[12]

Context

Our systematic review will be embedded in the context of existing research syntheses. To date, a substantial number of systematic reviews has been published that address different aspects of HIV-related stigma and discrimination. These include associations of stigma (covariates, causes or effects),[13-40] access to care,[41-66] HIV testing,[67-91] country-specific explorations of stigma or discrimination,[92-109] the role of stigma and discrimination in treatment adherence,[110-127] HIV experiences of people living with HIV or their care givers,[128-141] stigma/discrimination reduction in the community,[142-153] HIV disclosure considerations,[154-162] stigma/discrimination in healthcare,[163-170] rights and regulations,[171-177] intersectionality,[178-183] measuring stigma,[184-189] HIV prevention,[190-193] stigma reduction in low income countries,[194-197] self-stigma,[7 198 199] and other, unique topics.[200 201] We build on these reviews which outline existing research and point to persistent knowledge gaps. The reviews will be instrumental for reference-mining to ensure that all relevant material has been considered.

We will identify and categorize existing systematic reviews to explore the research field further. Systematic reviews will be identified through PubMed (biomedical literature) using the systematic review filter, through PsycINFO (psychosocial literature) and Web of science (general science literature including legal and policy analysis), as well as through the Cochrane Database of Systematic Reviews (focus on health) and the Campbell Collaboration (focus on social sciences). Furthermore, we will search the review registries PROSPERO and Open Science Framework to ensure that all relevant registered systematic reviews have been identified.

For peer review only - http://bmjopen.bmj.com/site/about/guidelines.xhtml

Analytic framework

Given that there is no agreed nomenclature in this interdisciplinary field, we established working definitions of the concepts 'stigma,' 'internalized stigma' and 'discrimination' for the purpose of this systematic review:

- Stigma refers to beliefs and/or attitudes about HIV.
- Discrimination refers to the behaviors that result from attitudes or beliefs about HIV.
- Internalized stigma (self-stigma) refers to a person living with HIV internalizing negative attitudes associated with HIV and accepting these as applicable to themselves.
- Stigma and discrimination in healthcare refers to negative beliefs and behaviors based on perceived or actual HIV status experienced in healthcare delivery settings.
- Stigma in laws and policies refers to distinctions, exclusion or restriction based on perceived HIV status or membership of a group that is vulnerable to HIV.

The analytic framework depicted in Figure 1 maps the review's four Key Questions and outlines the review's content, i.e., frameworks, measures, and intervention evaluations, targeted in this systematic review.

The figure includes existing conceptual models for HIV-related stigma, including frameworks such as the global HIV stigma reduction framework,[202 203] and logic models addressing stigma and discrimination, potential causes or effects (Key question 1). The frameworks comprise of the underlying construct of stigma and/or discrimination and their components. The analytic framework shows also the measures, indicators, or operationalizations of the constructs stigma and discrimination that are used to measure and quantify stigma and discrimination (Key question 2). The arrow represents interventions aiming to prevent, reduce, or mitigate stigma and discrimination (Key question 3), as well as the 'critical success factors' of tested interventions (Key question 4).

The systematic review is focused on people with living or perceived to be living with HIV and people who are disproportionately affected by HIV. For the purposes of this review and based on the UNAIDS definition, the latter group includes gay men and other men who have sex with men, sex workers, transgender people, people who inject drugs and prisoners and other incarcerated people.[204]

Search strategy

To identify primary research studies, we will search the health-specific research database PubMed, in particular to identify research on stigma experienced in healthcare settings. We will search PsycINFO to identify psychological and social research on stigma and use the general scientific research database Web of Science, in particular to identify legal and policy analyses on stigma and discrimination. We will identify government and non-governmental organization reports indexed in the Universal Human Rights Index, HeinOnline, PAIS, and HIV Legal Network.

Additional grey literature searches will target organizations relevant to key populations affected by HIV and funders of stigma research. Specifically, we will search the websites of the IAS, UNAIDS, United Nations Development Programme (UNDP), STRIVE, Health Policy Plus, and Sage (resource-sharing community for Canadian HIV and hepatitis C service providers).

Targeted search strategies for each Key Question will combine free text search terms with controlled vocabulary of the individual databases. Draft search strategies are shown in the appendix. The searches will be designed, executed, and documented by an Evidence-based Practice Center librarian. The searches outside of research databases will be instrumental in identifying conceptual models to assess internal stigma and stigma entrenched in national laws and policies. The multiple sources and interdisciplinary approach aim to reduce selection bias being introduced in the literature review.

Eligibility criteria

We use a PICOTS (participant, independent variable, comparator or study design, outcome/measure, timing, and setting) framework to structure the eligibility criteria:

- Participants: People living with or perceived to be living with HIV and people from groups who are disproportionately affected by HIV infection. We will exclude studies of mixed populations and other participant targets unless the study provides HIV-relevant subgroup analyses.
- Independent variable: Frameworks will include models outlining and relating to multiple components of stigma and/or discrimination, including conceptual frameworks, logic models, taxonomies, and analytic models for assessment, prevention, reduction, or mitigation of stigma. Measures will include self and peer report measures used for formal assessment of

For peer review only

stigma and discrimination. Studies using a published measure will be included if the psychometric properties of the measure are a central focus of the study (e.g., evaluating the predictive validity of an existing measure). Eligible intervention evaluations may test strategies and policies aimed at preventing, reducing, or mitigating HIV-related stigma and discrimination. Intervention evaluations aimed at self-stigma, stigma and discrimination in healthcare, and stigma and discrimination at the legal or policy level are eligible. We will exclude publications exclusively addressing community and cultural stigma.

- Comparator / study design: Publications introducing frameworks will be included regardless of the comparator or study design. Measure research needs to describe the tool in sufficient detail to be included but needs no comparator. Experimental evaluations of interventions have to report on a concurrent comparator (e.g., randomized controlled trial, controlled clinical trial, comparative studies, quasi-experimental studies, natural experiments) to be eligible. Observational evaluations with interventions or policies not under the control of the investigator and influenced by secular trends, have to report on a historic or concurrent comparator (e.g., pre-post, cohort study comparing two cohorts) and be sufficiently large or encompassing to be eligible (either demonstrating statistical power in a power analysis, reporting on large-scale evaluations of 200 participants or more, or ensuring that all eligible units have been targeted such as all healthcare providers in a hospital received the intervention). For studies evaluating the impacts of laws, no comparator is required if the study author can demonstrate an alternative analysis of determining the effect of the law not compared to a prior period or different legislative environment. Systematic reviews will be retained for an umbrella review providing context and for reference-mining.
- Outcome: Framework publications will be included regardless of any reported outcomes. Measure research needs to report a detailed description of the measure, the development process, or the evaluation or validation of the measure. Intervention research needs to provide a structured evaluation of an indicator of stigma or operationalization of discrimination to be eligible.
- Timing: Frameworks will not be restricted by publication year. For measures and interventions, only those published from 2008 on will be included, building on the first People Living with HIV Stigma Index published in 2008, which transformed thinking around HIV-related stigma measurement.[205]
- Setting: The review is not restricted regarding setting but we will restrict to English language publications.

Data abstraction

For the frameworks, we will abstract the author group and publisher; publication year, scope, aim or purpose of the framework; subtype and definition of the constructs stigma and/or discrimination; addressed targets (e.g., people living with HIV and their families); framework components; and a broad summary of the framework. The Draft Evidence Table Key Question 1 will summarize the identified frameworks (see appendix).

For measures, we will document the author group; publication year; name of the tool; the stigma or discrimination subtype being assessed, the underlying framework (where applicable) and definitions of stigma and discrimination; the targeted population; the surveyed population used to develop or assess the measure; the scale structure of the tool, number of items, and answer mode; the documented reliability; and evidence of validity. Draft Evidence Table Key Question 2 outlines the evidence table documenting this information for each included publication (see appendix).

For intervention evaluations (Key Question 3), we will document the study identification details, year of publication, country, study design, sample size, participant details, context or setting; intervention type, intervention description, and intervention components, comparator type and comparator description, definition and measures of stigma and discrimination, and findings for the outcome measures. In order to document the effect of the intervention concisely, we will abstract data for the main effectiveness signal and adverse events or unintended consequences. In addition, we will record any information provided by the study authors on the appropriateness of the utilized measures, intervention or comparator. The draft evidence table is shown in the appendix (Draft Evidence Table Key Question 3).

For peer review only

Critical appraisal and analysis plan

For frameworks, we will assess the source (e.g., published by an individual author group or endorsement by a professional organization) and processes used to develop the framework, including stakeholder involvement and formal consensus finding methods.[206 207]

For measures, we will evaluate the demonstrated reliability (internal consistency, test-retest stability, rater agreement) as well as evidence of the validity (e.g., content, criterion, or construct validity) applying COSMIN (COnsensus-based Standards for the selection of health Measurement Instruments) criteria.[208]

Intervention evaluations will be evaluated for potential selection, detection, performance, attrition, reporting, and study-specific sources of bias, adapting RoB 2 and ROBINS-I criteria.[209 210]

To determine the effects of the interventions, we will compute measure-independent effect estimates for all included studies, i.e., standardized mean differences (SMD) for continuous outcomes and relative risks (RR) for categorical outcomes, to facilitate comparisons across studies. Interpretation of the effect sizes will take the statistical significance of the difference compared to the control or pre-intervention status into account as well as the statistical power of the study to detect an effect. Where possible, results of studies across interventions will be summarized in random effects meta-analyses applying Hartung-Knapp corrections for small samples.[211]

To explore which interventions are successful and to determine what characterizes these interventions, we will first broadly categorize the type of intervention. The categorization will be undertaken based on the abstracted intervention description and will be blind to the findings of the study. The categories will be used to identify subgroups of more homogeneous intervention types. In a second step, we will derive a set of common intervention components drawn from the identified literature and informed by existing intervention frameworks and taxonomies. The presence or absence of components will be documented for each successful and each unsuccessful intervention, together with context information such as the country of evaluation. The draft component tables for the successful and unsuccessful interventions are shown in the appendix (Draft Component Table Key Question 4a, Draft Component Table Key Question 4b).

Categorizing the evaluations as successful or unsuccessful will follow a transparent algorithm and will be determined by two independent literature reviewers to reduce errors and bias. For all studies, the reduction of stigma will be determined. Assessments will be based on the results in the intervention group relative to a control group. In the absence of a concurrent control group, the change compared to the pre-intervention status will be used to determine intervention success. Categorizing interventions as successful or unsuccessful will qualitatively stratify the identified research. We acknowledge that this step will lose the granularity of a continuous variable. However, we anticipate that a broad categorization is necessary given the diversity of the approaches. We will use transparent methods to categorize studies and discrepancies in the effect classification between reviewer ratings will be resolved through discussion in the review team.

Determining characteristics of successful interventions and success factors in often complex and multi-component interventions, as well as reasons for failure, will require a careful analysis of the components or ‘active ingredients’ of the interventions. We will apply principles of qualitative comparative analysis (QCA) and review the established component matrix across the successful interventions in seeking to determine which individual and figurations of factors appear to be associated with success.[212] We will explore differences in the component structure between interventions that were determined to be successful and those that were determined to be unsuccessful to identify components likely be associated with success. We will conduct meta-regressions across studies to confirm effects of components. Meta-regressions will add the component of interest to the meta-analytic model to determine whether its presence or absence affects the size of the intervention effect.

Summary of findings and body of evidence assessment

We will document transparent criteria to evaluate the certainty in the evidence across the included research. We will adapt the eight GRADE (Grading of Recommendations, Assessment, Development, and Evaluation) criteria *study limitation, inconsistency, imprecision, indirectness, reporting bias to upgrade and the criteria large effect, dose-response relationship, and confounding would mask an effect*, to downgrade the quality of evidence.[213] While the criterion *study limitation* is applicable to all key questions, *inconsistency* (e.g., of reliability estimates across studies) will be used to interpret the quality of evidence for Key Question 2, 3, and 4, and the criterion *imprecision* (e.g., range of reported

For peer review only

reliability estimates) will be applied to Key Question 2 and 3. All other criteria will primarily apply to Key Question 3 and the evidence statements that can be formulated to answer this key question.

The evaluation of the body of evidence will be used to arrive at internationally accepted certainty categories that communicate our confidence in the findings using the categories *high*, *moderate, low*, and *very low*. *High* indicates confidence that the true effect is similar to the estimated effect, *moderate* indicates the true effect is probably close to the estimated effect, *low* suggests the true effect might be markedly different from the estimated effect, and *very low* signals that the true effect is probably markedly different from the estimated effect. We will review the appropriateness of the starting point of low quality of evidence for non-randomized studies before upgrading or downgrading of the evidence to avoid floor effects and ensure meaningful differentiation.

Synthesis

The identified evidence will be documented in comprehensive tables and figures. The literature flow will be documented in a literature flow diagram and account for all identified research. Critical appraisal of frameworks and measures will be integrated into the evidence tables. The risk of bias across intervention studies will be documented in a risk of bias figure showing the distribution for each criterion. All included research will be documented in concise evidence tables providing details for each included framework, measure, and intervention evaluation (see drafts in the appendix).

With this review, we intend to establish a compendium of existing frameworks for HIV-related stigma and discrimination. Frameworks published under Creative Commons licenses, and where copyright agreements allow these for other frameworks, will be shown in full to allow a meaningful overview. Where copyright assertions cannot be obtained, the framework evidence table will include the link to the framework if available in the public domain.

The evidence table for measures aims to provide a resource for future HIV stigma and discrimination research. It will provide a comprehensive overview of the available measures to help select tools for future studies.

The evidence table and component tables for the identified interventions aim to document the existing knowledge base for interventions to address self-stigma, stigma and discrimination in healthcare, and stigma and discrimination in laws and policies. A detailed exploration of intervention components aims to support efforts in prevention, reducing and mitigating HIV-related stigma and discrimination.

In addition to a narrative synthesis, we will document the evidence across research in summary of findings tables with one table for each key question. The table summarizing Key Question 1 will document the number and type of the identified stigma and discrimination frameworks. The summary of findings table summarizing Key Question 2 will document the number and type of identified stigma and discrimination measures, listing the measure details and reliability (evidence that the tool is measuring accurately and consistently) and validity (evidence that the tool is measuring what it is supposed to measure). The third table will document the evidence for the interventions (Key Question 3), organized by context and modality (e.g., healthcare facility intervention, law or policy change), and within by outcome category (effectiveness, unintended consequences), together with the quality of evidence assessment. The fourth summary of findings table will document commonalities of successful interventions and failed interventions (Key Question 4).

Gap analysis

Part of the systematic review will be a formal gap analysis. We will use a structured approach to document research gaps. Gaps will be documented in a Study design, Participants, Interventions, Framework, Outcomes, Context / Country (SPIFOC) framework, with attention to other relevant categories that may emerge during the evidence review. The gap analysis will document research needs and provide concrete recommendations for future research.

Patient and public involvement statement

The draft protocol was peer-reviewed by international content experts and a representative of the community of persons living with HIV to ensure that the review asks the right questions. Stakeholders will be asked to review the draft review to ensure that all relevant frameworks, measures, and intervention evaluations have been captured and that the review contributes meaningfully to the knowledge base and to ensure that the evidence review is as impactful as possible.

For peer review only

ETHICS AND DISSEMINATION

The systematic review conduct will be transparent and is designed to advance research to support people living with or suspected to be living with HIV and people disproportionally affected by HIV. The review is considered exempt as it does not involve human subjects and does not require review by the human subject protection committee.

The review will be registered in PROSPERO, the results will be published in a journal manuscript and presented at research conferences. The data will be made available in the Systematic Review Data Repository.[12]

ACKNOWLEDGEMENTS

We thank Lucy Stackpool-Moore, Tessa Oraro-Lawrence, Kasoka Kasoka, Brent Allan, and members of the country team for helpful comments.

CONTRIBUTOR AND GUARANTOR INFORMATION

SH, LF, and SG developed the systematic review, all authors (SH, LF, MB, SY, NF, AM, SC) contributed to this protocol. SH serves as guarantor and accepts full responsibility for the work and the conduct of the study, had access to data, and controlled the decision to publish. The corresponding author attests that all listed authors meet authorship criteria and that no others meeting the criteria have been omitted.

COMPETING INTERESTS STATEMENT

None of the authors have any conflicts of interest.

FUNDING STATEMENT

This work (Global Systematic Evidence Review: Getting to the Heart of Stigma) is supported by the International AIDS Society (IAS). The funder had no role in the development of the protocol or decision to submit this manuscript.

REFERENCES

1. Goffman E. Stigma: Notes on the management of spoiled identity. Englewood Cliffs, New Jersey: Prentice-Hall 1963.

2. Hatzenbuehler ML, Phelan JC, Link BG. Stigma as a fundamental cause of population health inequalities. *Am J Public Health* 2013;103(5):813-21. doi: 10.2105/ajph.2012.301069 [published Online First: 2013/03/16]

3. Relf MV, W LH, Holt L, et al. A Review of the State of the Science of HIV and Stigma: Context, Conceptualization, Measurement, Interventions, Gaps, and Future Priorities. *J Assoc Nurses AIDS Care* 2021;32(3):392-407. doi: 10.1097/JNC.0000000000000237 [published Online First: 2021/03/04]

4. Parker R, Aggleton P. HIV and AIDS-related stigma and discrimination: a conceptual framework and implications for action. *Soc Sci Med* 2003;57(1):13-24. doi: 10.1016/s0277-9536(02)00304-0 [published Online First: 2003/05/20]

5. Joint United Nations Programme on HIV/AIDS. Reduction of HIV-related stigma and discrimination: Guidance note. Geneva, Switzerland: UNAIDS,, 2014.

6. UN General Assembly. Declaration of Human Rights 10 December 1948, 217 A (III) [Available from: https://www.refworld.org/docid/3ae6b3712c.html [accessed 29 August 2021 accessed August 29, 2021.

7. Pantelic M, Steinert JI, Park J, et al. 'Management of a spoiled identity': systematic review of interventions to address self-stigma among people living with and affected by HIV. *BMJ Glob Health* 2019;4(2):e001285. doi: 10.1136/bmjgh-2018-001285 [published Online First: 2019/04/19]

8. UNAIDS. Evidence for eliminating HIV-related stigma and discrimination: Joint United Nations Programme on HIV/AIDS (UNAIDS), 2020.

For peer review only - http://bmjopen.bmj.com/site/about/guidelines.xhtml

9. UNAIDS. Global AIDS Strategy 2021-2026 — End Inequalities. End AIDS. Geneva, Switzerland: UNAIDS, 2021.

10. United Nations General Assembly. Political Declaration on HIV and AIDS: Ending Inequalities and Getting on Track to End AIDS by 2030. A/RES/75/284. New York, USA: United Nations, 2021.

11. UNAIDS. Mapping of tools on HIV-related stigma and discrimination in health care settings: Final Report, 2015.

12. Agency for Healthcare Research and Quality. SRDR Systematic Review Data Reposity [cited Protocol ref. Available from: https://srdr.ahrq.gov/ accessed May 11 2021.

13. Amare T, Getinet W, Shumet S, et al. Prevalence and Associated Factors of Depression among PLHIV in Ethiopia: Systematic Review and Meta-Analysis, 2017. *AIDS Res Treat* 2018;2018:5462959. doi: 10.1155/2018/5462959 [published Online First: 2018/04/24]

14. Ayano G, Solomon M, Abraha M. A systematic review and meta-analysis of epidemiology of depression in people living with HIV in east Africa. *BMC Psychiatry* 2018;18(1):254. doi: 10.1186/s12888-018-1835-3 [published Online First: 2018/08/17]

15. Bayat AH, Mohammadi R, Moradi-Joo M, et al. HIV and drug related stigma and risk-taking behaviors among people who inject drugs: a systematic review and meta-analysis. *J Addict Dis* 2020;38(1):71-83. doi: 10.1080/10550887.2020.1718264 [published Online First: 2020/03/19]

16. Belayneh Z, Mekuriaw B, Mehare T, et al. Magnitude and predictors of common mental disorder among people with HIV/AIDS in Ethiopia: a systematic review and meta-analysis. *BMC Public Health* 2020;20(1):689. doi: 10.1186/s12889-020-08800-8 [published Online First: 2020/05/16]

17. Dessauvagie AS, Jörns-Presentati A, Napp AK, et al. The prevalence of mental health problems in sub-Saharan adolescents living with HIV: a systematic review. *Glob Ment Health (Camb)* 2020;7:e29. doi: 10.1017/gmh.2020.18 [published Online First: 2021/01/26]

18. Evangeli M, Ferris K, Kenney NM, et al. A systematic review of psychological correlates of HIV testing intention. *Aids Care-Psychol Socio-Med Asp Aids-Hiv* 2018;30(1):18-26. doi: 10.1080/09540121.2017.1344351

19. Gesesew HA, Tesfay Gebremedhin A, Demissie TD, et al. Significant association between perceived HIV related stigma and late presentation for HIV/AIDS care in low and middle-income countries: A systematic review and meta-analysis. *PLoS One* 2017;12(3):e0173928. doi: 10.1371/journal.pone.0173928 [published Online First: 2017/03/31]

20. Ghiasvand H, Higgs P, Noroozi M, et al. Social and demographical determinants of quality of life in people who live with HIV/AIDS infection: evidence from a meta-analysis. *Biodemography Soc Biol* 2020;65(1):57-72. doi: 10.1080/19485565.2019.1587287 [published Online First: 2019/03/19]

21. Govindasamy D, Seeley J, Olaru ID, et al. Informing the measurement of wellbeing among young people living with HIV in sub-Saharan Africa for policy evaluations: a mixed-methods systematic review. *Health Qual Life Outcomes* 2020;18(1):120. doi: 10.1186/s12955-020-01352-w [published Online First: 2020/05/07]

22. Griswold MK, Pagano-Therrien J. Women Living With HIV in High Income Countries and the Deeper Meaning of Breastfeeding Avoidance: A Metasynthesis. *J Hum Lact* 2020;36(1):44-52. doi: 10.1177/0890334419886565 [published Online First: 2020/01/03]

23. Haines C, Loades ME, Coetzee BJ, et al. Which HIV-infected youth are at risk of developing depression and what treatments help? A systematic review focusing on Southern Africa. *Int J Adolesc Med Health* 2019 doi: 10.1515/ijamh-2019-0037 [published Online First: 2019/08/09]

24. Ishola F. EFFECT OF HIV RELATED STIGMA ON UTILISATION OF SKILLED BIRTH ATTENDANTS BY HIV POSITIVE WOMEN IN NIGERIA; A SYSTEMATIC REVIEW. *Sex Transm Infect* 2017;93:A207-A07. doi: 10.1136/sextrans-2017-053264.538

For peer review only

25. Li H, Li X, Zhang L, et al. Effects of multiple types of stigma on the probability of HIV disclosure to sex partners: a systematic review. *Sex Health* 2016;13(6):516-29. doi: 10.1071/sh16089 [published Online First: 2016/08/06]

26. MacCarthy S, Poteat T, Xia Z, et al. Current research gaps: a global systematic review of HIV and sexually transmissible infections among transgender populations. *Sex Health* 2017;14(5):456-68. doi: 10.1071/sh17096 [published Online First: 2017/12/09]

27. MacLean JR, Wetherall K. The Association between HIV-Stigma and Depressive Symptoms among People Living with HIV/AIDS: A Systematic Review of Studies Conducted in South Africa. *J Affect Disord* 2021;287:125-37. doi: 10.1016/j.jad.2021.03.027 [published Online First: 2021/03/30]

28. Magno L, Silva L, Veras MA, et al. Stigma and discrimination related to gender identity and vulnerability to HIV/AIDS among transgender women: a systematic review. *Cad Saude Publica* 2019;35(4):e00112718. doi: 10.1590/0102-311x00112718 [published Online First: 2019/04/18]

29. Narasimhan M, Payne C, Caldas S, et al. Ageing and healthy sexuality among women living with HIV. *Reprod Health Matters* 2016;24(48):43-51. doi: 10.1016/j.rhm.2016.11.001 [published Online First: 2016/12/28]

30. Necho M, Belete A, Tsehay M. Depressive symptoms and their determinants in patients who are on antiretroviral therapy in the case of a low-income country, Ethiopia: a systematic review and meta-analysis. *Int J Ment Health Syst* 2021;15(1):3. doi: 10.1186/s13033-020-00430-2 [published Online First: 2021/01/08]

31. Necho M, Tsehay M, Zenebe Y. Suicidal ideation, attempt, and its associated factors among HIV/AIDS patients in Africa: a systematic review and meta-analysis study. *Int J Ment Health Syst* 2021;15(1):13. doi: 10.1186/s13033-021-00437-3 [published Online First: 2021/01/25]

32. Pantelic M, Shenderovich Y, Cluver L, et al. Predictors of internalised HIV-related stigma: a systematic review of studies in sub-Saharan Africa. *Health Psychol Rev* 2015;9(4):469-90. doi: 10.1080/17437199.2014.996243 [published Online First: 2015/01/07]

33. Rueda S, Mitra S, Chen S, et al. Examining the associations between HIV-related stigma and health outcomes in people living with HIV/AIDS: a series of meta-analyses. *BMJ Open* 2016;6(7):e011453. doi: 10.1136/bmjopen-2016-011453 [published Online First: 2016/07/15]

34. Rzeszutek M, Gruszczyńska E. Posttraumatic growth among people living with HIV: A systematic review. *J Psychosom Res* 2018;114:81-91. doi: 10.1016/j.jpsychores.2018.09.006 [published Online First: 2018/10/14]

35. Scofield D, Moseholm E. HIV-related stigma and health-related quality of life in women living with HIV in developed countries: a systematic review. *Aids Care-Psychol Socio-Med Asp Aids-Hiv* 2021:9. doi: 10.1080/09540121.2021.1891193

36. Scott W, Arkuter C, Kioskli K, et al. Psychosocial factors associated with persistent pain in people with HIV: a systematic review with meta-analysis. *Pain* 2018;159(12):2461-76. doi: 10.1097/j.pain.0000000000001369 [published Online First: 2018/08/22]

37. Yassin Z, Erasmus C, Frantz J. HIV-related stigma and the psychosocial well-being of children orphaned by AIDS: A systematic review. *Vulnerable Children and Youth Studies* 2018;13(3):247-58. doi: http://dx.doi.org/10.1080/17450128.2018.1457193

38. Yassin Z, Erasmus CJ. The impact of HIV-related stigma on the psychological well-being of children who have been orphaned by AIDS. *Vulnerable Children and Youth Studies* 2016;11(4):297-323. doi: http://dx.doi.org/10.1080/17450128.2016.1214769

39. Zgambo M, Kalembo FW, Mbakaya BC. Risky behaviours and their correlates among adolescents living with HIV in sub-Saharan Africa: a systematic review. *Reprod Health* 2018;15(1):180. doi: 10.1186/s12978-018-0614-4 [published Online First: 2018/10/26]

For peer review only - http://bmjopen.bmj.com/site/about/guidelines.xhtml

40. Zhao Y, Luo T, Tucker JD, et al. Risk Factors of HIV and Other Sexually Transmitted Infections in China: A Systematic Review of Reviews. *PLoS One* 2015;10(10):e0140426. doi: 10.1371/journal.pone.0140426 [published Online First: 2015/10/16]

41. Aggarwal NK, Consavage KE, Dhanuka I, et al. Health and Health Care Access Barriers Among Transgender Women Engaged in Sex Work: A Synthesis of US-Based Studies Published 2005-2019. *Lgbt Health* 2021;8(1):11-25. doi: 10.1089/lgbt.2019.0243

42. Ahmed S, Autrey J, Katz IT, et al. Why do people living with HIV not initiate treatment? A systematic review of qualitative evidence from low- and middle-income countries. *Soc Sci Med* 2018;213:72-84. doi: 10.1016/j.socscimed.2018.05.048 [published Online First: 2018/07/31]

43. Bass SB, D'Avanzo P, Alhajji M, et al. Exploring the Engagement of Racial and Ethnic Minorities in HIV Treatment and Vaccine Clinical Trials: A Scoping Review of Literature and Implications for Future Research. *Aids Patient Care and Stds* 2020;34(9):399-416. doi: 10.1089/apc.2020.0008

44. Brookfield S, Dean J, Forrest C, et al. Barriers to Accessing Sexual Health Services for Transgender and Male Sex Workers: A Systematic Qualitative Meta-summary. *AIDS Behav* 2020;24(3):682-96. doi: 10.1007/s10461-019-02453-4 [published Online First: 2019/03/15]

45. Ferguson L, Grant AD, Watson-Jones D, et al. Linking women who test HIV-positive in pregnancy-related services to long-term HIV care and treatment services: a systematic review. *Trop Med Int Health* 2012;17(5):564-80. doi: 10.1111/j.1365-3156.2012.02958.x [published Online First: 2012/03/08]

46. Ganle JK, Baatiema L, Quansah R, et al. Barriers facing persons with disability in accessing sexual and reproductive health services in sub-Saharan Africa: A systematic review. *Plos One* 2020;15(10):19. doi: 10.1371/journal.pone.0238585

47. Gari S, Doig-Acuña C, Smail T, et al. Access to HIV/AIDS care: a systematic review of socio-cultural determinants in low and high income countries. *BMC Health Serv Res* 2013;13:198. doi: 10.1186/1472-6963-13-198 [published Online First: 2013/05/30]

48. Gonçalves TR, Costa AHC, Sales MS, et al. [Combined HIV prevention? Systematic review of interventions with women from low- and middle-income countries]. *Cien Saude Colet* 2020;25(5):1897-912. doi: 10.1590/1413-81232020255.15832018 [published Online First: 2020/05/14]

49. Gourlay A, Birdthistle I, Mburu G, et al. Barriers and facilitating factors to the uptake of antiretroviral drugs for prevention of mother-to-child transmission of HIV in sub-Saharan Africa: a systematic review. *J Int AIDS Soc* 2013;16(1):18588. doi: 10.7448/ias.16.1.18588

18588 [published Online First: 2013/07/23]

50. Govindasamy D, Ford N, Kranzer K. Risk factors, barriers and facilitators for linkage to antiretroviral therapy care: a systematic review. *Aids* 2012;26(16):2059-67. doi: 10.1097/QAD.0b013e3283578b9b [published Online First: 2012/07/12]

51. Hodgson I, Plummer ML, Konopka SN, et al. A systematic review of individual and contextual factors affecting ART initiation, adherence, and retention for HIV-infected pregnant and postpartum women. *PLoS One* 2014;9(11):e111421. doi: 10.1371/journal.pone.0111421 [published Online First: 2014/11/06]

52. Jones C, Ritchwood TD, Taggart T. Barriers and Facilitators to the Successful Transition of Adolescents Living with HIV from Pediatric to Adult Care in Low and Middle-Income Countries: A Systematic Review and Policy Analysis. *AIDS Behav* 2019;23(9):2498-513. doi: 10.1007/s10461-019-02621-6 [published Online First: 2019/08/05]

53. Kendrick HM. Are religion and spirituality barriers or facilitators to treatment for HIV: a systematic review of the literature. *Aids Care-Psychol Socio-Med Asp Aids-Hiv* 2017;29(1):1-13. doi: 10.1080/09540121.2016.1201196

For peer review only

For peer review only - http://bmjopen.bmj.com/site/about/guidelines.xhtml

54. Lancaster KE, Cernigliaro D, Zulliger R, et al. HIV care and treatment experiences among female sex workers living with HIV in sub-Saharan Africa: A systematic review. *Afr J AIDS Res* 2016;15(4):377-86. doi: 10.2989/16085906.2016.1255652 [published Online First: 2016/12/16]

55. Matacotta JJ, Rosales-Perez FJ, Carrillo CM. HIV Preexposure Prophylaxis and Treatment as Prevention - Beliefs and Access Barriers in Men Who Have Sex With Men (MSM) and Transgender Women: A Systematic Review. *J Patient Cent Res Rev* 2020;7(3):265-74. doi: 10.17294/2330-0698.1737 [published Online First: 2020/08/08]

56. Mathias A, Alves Dos Santos L, Grangeiro A, et al. Thematic synthesis HIV prevention qualitative studies in men who have sex with men (MSM). *Colomb Med (Cali)* 2019;50(3):201-14. doi: 10.25100/cm.v50i3.4078 [published Online First: 2020/04/15]

57. Mwaturura C, Traeger M, Lemoh C, et al. Barriers and facilitators to pre-exposure prophylaxis among African migrants in high income countries: a systematic review. *Sexual Health* 2021:10. doi: 10.1071/sh20175

58. O'Brien N, Hong QN, Law S, et al. Health System Features That Enhance Access to Comprehensive Primary Care for Women Living with HIV in High-Income Settings: A Systematic Mixed Studies Review. *AIDS Patient Care STDS* 2018;32(4):129-48. doi: 10.1089/apc.2017.0305 [published Online First: 2018/04/10]

59. Pellowski JA. Barriers to care for rural people living with HIV: a review of domestic research and health care models. *J Assoc Nurses AIDS Care* 2013;24(5):422-37. doi: 10.1016/j.jana.2012.08.007 [published Online First: 2013/01/29]

60. Peng P, Su S, Fairley CK, et al. A Global Estimate of the Acceptability of Pre-exposure Prophylaxis for HIV Among Men Who have Sex with Men: A Systematic Review and Meta-analysis. *AIDS Behav* 2018;22(4):1063-74. doi: 10.1007/s10461-017-1675-z [published Online First: 2017/02/09]

61. Rade DA, Crawford G, Lobo R, et al. Sexual Health Help-Seeking Behavior among Migrants from Sub-Saharan Africa and South East Asia living in High Income Countries: A Systematic Review. *Int J Environ Res Public Health* 2018;15(7) doi: 10.3390/ijerph15071311 [published Online First: 2018/06/23]

62. Russ S, Zhang C, Liu Y. Pre-Exposure Prophylaxis Care Continuum, Barriers, and Facilitators among Black Men Who Have Sex with Men in the United States: A Systematic Review and Meta-Analysis. *Aids and Behavior* 2020:11. doi: 10.1007/s10461-020-03156-x

63. Scholl E. Improving outpatient implementation of preexposure prophylaxis in men who have sex with men. *Journal of the American Association of Nurse Practitioners* 2016;28(8):446-52. doi: http://dx.doi.org/10.1002/2327-6924.12344

64. Wang Z, Yuan T, Fan S, et al. HIV Nonoccupational Postexposure Prophylaxis Among Men Who Have Sex with Men: A Systematic Review and Meta-Analysis of Global Data. *AIDS Patient Care STDS* 2020;34(5):193-204. doi: 10.1089/apc.2019.0313 [published Online First: 2020/05/13]

65. Wao H, Aluoch M, Odondi GO, et al. MSM's Versus Health Care Providers' Perceptions of Barriers to Uptake of HIV/AIDS-Related Interventions: Systematic Review and Meta-Synthesis of Qualitative and Quantitative Evidence. *Int J Sex Health* 2016;28(2):151-62. doi: 10.1080/19317611.2016.1153560

66. Young T, Busgeeth K. Home-based care for reducing morbidity and mortality in people infected with HIV/AIDS. *Cochrane Database Syst Rev* 2010(1):Cd005417. doi: 10.1002/14651858.CD005417.pub2 [published Online First: 2010/01/22]

67. Alvarez-del Arco D, Monge S, Azcoaga A, et al. HIV testing and counselling for migrant populations living in high-income countries: a systematic review. *Eur J Public Health* 2013;23(6):1039-45. doi: 10.1093/eurpub/cks130 [published Online First: 2012/09/25]

For peer review only

68. Blackstone SR, Nwaozuru U, Iwelunmor J. Antenatal HIV Testing in Sub-Saharan Africa During the Implementation of the Millennium Development Goals: A Systematic Review Using the PEN-3 Cultural Model. *Int Q Community Health Educ* 2018;38(2):115-28. doi: 10.1177/0272684x17749576 [published Online First: 2017/12/23]

69. Bolsewicz K, Vallely A, Debattista J, et al. Factors impacting HIV testing: A review—Perspectives from Australia, Canada, and the UK. *AIDS Care* 2015;27(5):570-80. doi: http://dx.doi.org/10.1080/09540121.2014.986050

70. Campbell CK, Lippman SA, Moss N, et al. Strategies to Increase HIV Testing Among MSM: A Synthesis of the Literature. *AIDS Behav* 2018;22(8):2387-412. doi: 10.1007/s10461-018-2083-8 [published Online First: 2018/03/20]

71. Chamla D, Luo C, Adjorlolo-Johnson G, et al. Integration of HIV infant testing into immunization programmes: a systematic review. *Paediatr Int Child Health* 2015;35(4):298-304. doi: 10.1080/20469047.2015.1109233 [published Online First: 2016/01/09]

72. Duffy M, Sharer M, Berhan A, et al. HIV risk screening for high-yield community testing services for orphans and vulnerable children: A literature review. *Vulnerable Children and Youth Studies* 2018;13(2):95-115. doi: http://dx.doi.org/10.1080/17450128.2017.1332399

73. Evangeli M, Pady K, Wroe AL. Which Psychological Factors are Related to HIV Testing? A Quantitative Systematic Review of Global Studies. *AIDS Behav* 2016;20(4):880-918. doi: 10.1007/s10461-015-1246-0 [published Online First: 2015/11/15]

74. Feyissa GT, Lockwood C, Munn Z. The effectiveness of home-based HIV counseling and testing on reducing stigma and risky sexual behavior among adults and adolescents: A systematic review and meta-analyses. *JBI Database System Rev Implement Rep* 2015;13(6):318-72. doi: 10.11124/jbisrir-2015-2235 [published Online First: 2015/10/13]

75. Hamilton A, Shin S, Taggart T, et al. HIV testing barriers and intervention strategies among men, transgender women, female sex workers and incarcerated persons in the Caribbean: a systematic review. *Sex Transm Infect* 2020;96(3):189-96. doi: 10.1136/sextrans-2018-053932 [published Online First: 2019/09/12]

76. Hamilton A, Thompson N, Choko AT, et al. HIV Self-Testing Uptake and Intervention Strategies Among Men in Sub-Saharan Africa: A Systematic Review. *Front Public Health* 2021;9:594298. doi: 10.3389/fpubh.2021.594298 [published Online First: 2021/03/09]

77. Krause J, Subklew-Sehume F, Kenyon C, et al. Acceptability of HIV self-testing: a systematic literature review. *BMC Public Health* 2013;13:735. doi: 10.1186/1471-2458-13-735 [published Online First: 2013/08/09]

78. Laprise C, Bolster-Foucault C. Understanding barriers and facilitators to HIV testing in Canada from 2009-2019: A systematic mixed studies review. *Can Commun Dis Rep* 2021;47(2):105-25. doi: 10.14745/ccdr.v47i02a03 [published Online First: 2021/03/23]

79. Leidel S, Wilson S, McConigley R, et al. Health-care providers' experiences with opt-out HIV testing: a systematic review. *AIDS Care* 2015;27(12):1455-67. doi: 10.1080/09540121.2015.1058895 [published Online First: 2015/08/15]

80. Lorenc T, Marrero-Guillamón I, Llewellyn A, et al. HIV testing among men who have sex with men (MSM): systematic review of qualitative evidence. *Health Educ Res* 2011;26(5):834-46. doi: 10.1093/her/cyr064 [published Online First: 2011/08/30]

81. Musheke M, Ntalasha H, Gari S, et al. A systematic review of qualitative findings on factors enabling and deterring uptake of HIV testing in Sub-Saharan Africa. *BMC Public Health* 2013;13:220. doi: 10.1186/1471-2458-13-220 [published Online First: 2013/03/19]

For peer review only

82. Nnko S, Kuringe E, Nyato D, et al. Determinants of access to HIV testing and counselling services among female sex workers in sub-Saharan Africa: a systematic review. *BMC Public Health* 2019;19(1):15. doi: 10.1186/s12889-018-6362-0 [published Online First: 2019/01/07]

83. Pant Pai N, Sharma J, Shivkumar S, et al. Supervised and unsupervised self-testing for HIV in high- and low-risk populations: a systematic review. *PLoS Med* 2013;10(4):e1001414. doi: 10.1371/journal.pmed.1001414 [published Online First: 2013/04/09]

84. Pottie K, Medu O, Welch V, et al. Effect of rapid HIV testing on HIV incidence and services in populations at high risk for HIV exposure: an equity-focused systematic review. *BMJ Open* 2014;4(12):e006859. doi: 10.1136/bmjopen-2014-006859 [published Online First: 2014/12/17]

85. Qiao S, Zhou GY, Li XM. Disclosure of Same-Sex Behaviors to Health-care Providers and Uptake of HIV Testing for Men Who Have Sex With Men: A Systematic Review. *Am J Mens Health* 2018;12(5):1197-214. doi: 10.1177/1557988318784149

86. Qin YL, Han L, Babbitt A, et al. Experiences using and organizing HIV self-testing. *Aids* 2018;32(3):371-81. doi: 10.1097/qad.0000000000001705

87. Stannah J, Dale E, Elmes J, et al. HIV testing and engagement with the HIV treatment cascade among men who have sex with men in Africa: a systematic review and meta-analysis. *Lancet HIV* 2019;6(11):e769-e87. doi: 10.1016/s2352-3018(19)30239-5 [published Online First: 2019/10/12]

88. Stojanovski K, Naja-Riese G, King EJ, et al. A Systematic Review of the Social Network Strategy to Optimize HIV Testing in Key Populations to End the Epidemic in the United States. *AIDS Behav* 2021:1-19. doi: 10.1007/s10461-021-03259-z [published Online First: 2021/04/20]

89. Tan K, Black BP. A Systematic Review of Health Care Provider-Perceived Barriers and Facilitators to Routine HIV Testing in Primary Care Settings in the Southeastern United States. *J Assoc Nurses Aids Care* 2018;29(3):357-70. doi: 10.1016/j.jana.2017.12.006

90. Tokar A, Broerse JEW, Blanchard J, et al. HIV Testing and Counseling Among Female Sex Workers: A Systematic Literature Review. *AIDS Behav* 2018;22(8):2435-57. doi: 10.1007/s10461-018-2043-3 [published Online First: 2018/02/22]

91. Witzel TC, Lora W, Lees S, et al. Uptake contexts and perceived impacts of HIV testing and counselling among adults in East and Southern Africa: A meta-ethnographic review. *PLoS One* 2017;12(2):e0170588. doi: 10.1371/journal.pone.0170588 [published Online First: 2017/02/17]

92. Adeoye-Agboola DI, Evans H, Hewson D, et al. Factors influencing HIV disclosure among people living with HIV/AIDS in Nigeria: a systematic review using narrative synthesis and meta-analysis. *Public Health* 2016;136:13-28. doi: 10.1016/j.puhe.2016.02.021 [published Online First: 2016/04/10]

93. Azétsop J, Diop BA. Access to antiretroviral treatment, issues of well-being and public health governance in Chad: What justifies the limited success of the universal access policy? *Philosophy, Ethics, and Humanities in Medicine* 2013;8:14. doi: http://dx.doi.org/10.1186/1747-5341-8-8

94. Belay GM, Endalamaw A, Ayele AD. Late presentation of HIV positive adults and its predictors to HIV/AIDS care in Ethiopia: a systematic review and meta-analysis. *BMC Infect Dis* 2019;19(1):534. doi: 10.1186/s12879-019-4156-3 [published Online First: 2019/06/19]

95. Bharat S. A systematic review of HIV/AIDS-related stigma and discrimination in India: current understanding and future needs. *Sahara j* 2011;8(3):138-49. doi: 10.1080/17290376.2011.9724996 [published Online First: 2011/09/01]

96. Bradley E, Forsberg K, Betts JE, et al. Factors Affecting Pre-Exposure Prophylaxis Implementation for Women in the United States: A Systematic Review. *J Womens Health (Larchmt)* 2019;28(9):1272-85. doi: 10.1089/jwh.2018.7353 [published Online First: 2019/06/11]

For peer review only

97. Darlington CK, Hutson SP. Understanding HIV-related stigma among women in the Southern United States: A literature review. *AIDS and Behavior* 2017;21(1):12-26. doi: http://dx.doi.org/10.1007/s10461-016-1504-9

98. Das S, Leibowitz GS. Mental health needs of people living with HIV/AIDS in India: a literature review. *AIDS Care* 2011;23(4):417-25. doi: 10.1080/09540121.2010.507752 [published Online First: 2010/12/15]

99. Kerr ZY, Miller KR, Galos D, et al. Challenges, coping strategies, and recommendations related to the HIV services field in the HAART era: a systematic literature review of qualitative studies from the United States and Canada. *AIDS Patient Care STDS* 2013;27(2):85-95. doi: 10.1089/apc.2012.0356 [published Online First: 2013/01/23]

100. Loutfy M, Tharao W, Logie C, et al. Systematic review of stigma reducing interventions for African/Black diasporic women. *J Int AIDS Soc* 2015;18(1):19835. doi: 10.7448/ias.18.1.19835

19835 [published Online First: 2015/04/12]

101. Mey A, Plummer D, Dukie S, et al. Motivations and Barriers to Treatment Uptake and Adherence Among People Living with HIV in Australia: A Mixed-Methods Systematic Review. *AIDS Behav* 2017;21(2):352-85. doi: 10.1007/s10461-016-1598-0 [published Online First: 2016/11/09]

102. Monteiro SS, Brigeiro M, Vilella WV, et al. Challenges facing HIV treatment as prevention in Brazil: an analysis drawing on literature on testing. *Cienc Saude Coletiva* 2019;24(5):1792-806. doi: 10.1590/1413-81232018245.16512017

103. Sangaramoorthy T, Jamison A, Dyer T. Older African Americans and the HIV Care Continuum: A Systematic Review of the Literature, 2003-2018. *AIDS Behav* 2019;23(4):973-83. doi: 10.1007/s10461-018-2354-4 [published Online First: 2018/12/07]

104. Vaitses Fontanari AM, Zanella GI, Feijó M, et al. HIV-related care for transgender people: A systematic review of studies from around the world. *Soc Sci Med* 2019;230:280-94. doi: 10.1016/j.socscimed.2019.03.016 [published Online First: 2019/04/30]

105. Weihs M, Meyer-Weitz A. Barriers to workplace HIV testing in South Africa: a systematic review of the literature. *AIDS Care* 2016;28(4):495-9. doi: 10.1080/09540121.2015.1109586 [published Online First: 2015/11/13]

106. Weldesenbet AB, Kebede SA, Tusa BS. The Effect of Poor Social Support on Depression among HIV/AIDS Patients in Ethiopia: A Systematic Review and Meta-Analysis. *Depress Res Treat* 2020;2020:6633686. doi: 10.1155/2020/6633686 [published Online First: 2021/01/26]

107. Whembolua GL, Conserve DF, Thomas K, et al. HIV serostatus disclosure in the Democratic Republic of the Congo: a systematic review. *AIDS Care* 2019;31(4):489-93. doi: 10.1080/09540121.2018.1510103 [published Online First: 2018/08/17]

108. Wirtz AL, Kirey A, Peryskina A, et al. Uncovering the epidemic of HIV among men who have sex with men in Central Asia. *Drug and Alcohol Dependence* 2013;132(Suppl 1):S17-S24. doi: http://dx.doi.org/10.1016/j.drugalcdep.2013.06.031

109. Young C. Understanding HIV-related posttraumatic stress disorder in South Africa: A review and conceptual framework. *African Journal of AIDS Research* 2011;10(2):139-48. doi: http://dx.doi.org/10.2989/16085906.2011.593376

110. Ammon N, Mason S, Corkery JM. Factors impacting antiretroviral therapy adherence among human immunodeficiency virus-positive adolescents in Sub-Saharan Africa: a systematic review. *Public Health* 2018;157:20-31. doi: 10.1016/j.puhe.2017.12.010 [published Online First: 2018/03/05]

111. Campbell L, Masquillier C, Thunnissen E, et al. Social and Structural Determinants of Household Support for ART Adherence in Low- and Middle-Income Countries: A Systematic Review *Int J*

For peer review only

Environ Res Public Health 2020;17(11) doi: 10.3390/ijerph17113808 [published Online First: 2020/05/31]

112. Edeza A, Karina Santamaria E, Valente PK, et al. Experienced barriers to adherence to pre-exposure prophylaxis for HIV prevention among MSM: a systematic review and meta-ethnography of qualitative studies. *AIDS Care* 2020:1-9. doi: 10.1080/09540121.2020.1778628 [published Online First: 2020/06/13]

113. Geter A, Sutton MY, Hubbard McCree D. Social and structural determinants of HIV treatment and care among black women living with HIV infection: a systematic review: 2005-2016. *AIDS Care* 2018;30(4):409-16. doi: 10.1080/09540121.2018.1426827 [published Online First: 2018/01/30]

114. Heestermans T, Browne JL, Aitken SC, et al. Determinants of adherence to antiretroviral therapy among HIV-positive adults in sub-Saharan Africa: a systematic review. *BMJ Glob Health* 2016;1(4):e000125. doi: 10.1136/bmjgh-2016-000125 [published Online First: 2017/06/08]

115. Katz IT, Ryu AE, Onuegbu AG, et al. Impact of HIV-related stigma on treatment adherence: systematic review and meta-synthesis. *J Int AIDS Soc* 2013;16(3 Suppl 2):18640. doi: 10.7448/ias.16.3.18640

18640 [published Online First: 2013/11/28]

116. Knettel BA, Cichowitz C, Ngocho JS, et al. Retention in HIV Care During Pregnancy and the Postpartum Period in the Option B+ Era: Systematic Review and Meta-Analysis of Studies in Africa. *J Acquir Immune Defic Syndr* 2018;77(5):427-38. doi: 10.1097/qai.0000000000001616 [published Online First: 2017/12/30]

117. Lambert CC, Mugavero MJ, Najjar YS, et al. The State of Adherence to HIV Care in Black Women. *J Assoc Nurses AIDS Care* 2018;29(4):487-503. doi: 10.1016/j.jana.2018.02.008 [published Online First: 2018/05/17]

118. Makanjuola T, Taddese HB, Booth A. Factors associated with adherence to treatment with isoniazid for the prevention of tuberculosis amongst people living with HIV/AIDS: a systematic review of qualitative data. *PLoS One* 2014;9(2):e87166. doi: 10.1371/journal.pone.0087166 [published Online First: 2014/02/06]

119. Maxwell S, Gafos M, Shahmanesh M. Pre-exposure Prophylaxis Use and Medication Adherence Among Men Who Have Sex With Men: A Systematic Review of the Literature. *J Assoc Nurses AIDS Care* 2019;30(4):e38-e61. doi: 10.1097/jnc.0000000000000105 [published Online First: 2019/06/27]

120. Omonaiye O, Kusljic S, Nicholson P, et al. Medication adherence in pregnant women with human immunodeficiency virus receiving antiretroviral therapy in sub-Saharan Africa: a systematic review. *BMC Public Health* 2018;18(1):805. doi: 10.1186/s12889-018-5651-y [published Online First: 2018/06/28]

121. Paramesha AE, Chacko LK. Predictors of adherence to antiretroviral therapy among PLHIV. *Indian J Public Health* 2019;63(4):367-+. doi: 10.4103/ijph.IJPH_376_18

122. Shubber Z, Ford N. Adherence to HIV post-exposure prophylaxis for children/adolescents who have been sexually assaulted: A systematic review of barriers, enablers, and interventions. *Child Abuse Negl* 2019:104143. doi: 10.1016/j.chiabu.2019.104143 [published Online First: 2019/09/17]

123. Shubber Z, Mills EJ, Nachega JB, et al. Patient-Reported Barriers to Adherence to Antiretroviral Therapy: A Systematic Review and Meta-Analysis. *PLoS Med* 2016;13(11):e1002183. doi: 10.1371/journal.pmed.1002183 [published Online First: 2016/11/30]

124. Sidebottom D, Ekström AM, Strömdahl S. A systematic review of adherence to oral pre-exposure prophylaxis for HIV - how can we improve uptake and adherence? *BMC Infect Dis* 2018;18(1):581. doi: 10.1186/s12879-018-3463-4 [published Online First: 2018/11/18]

For peer review only

125. Sweeney SM, Vanable PA. The Association of HIV-Related Stigma to HIV Medication Adherence: A Systematic Review and Synthesis of the Literature. *AIDS Behav* 2016;20(1):29-50. doi: 10.1007/s10461-015-1164-1 [published Online First: 2015/08/26]

126. Wasti SP, van Teijlingen E, Simkhada P, et al. Factors influencing adherence to antiretroviral treatment in Asian developing countries: a systematic review. *Trop Med Int Health* 2012;17(1):71-81. doi: 10.1111/j.1365-3156.2011.02888.x [published Online First: 2011/10/05]

127. Zuniga JA, Wright C, Fordyce J, et al. Self-Management of HIV and Diabetes in African American Women: A Systematic Review of Qualitative Literature. *Diabetes Educ* 2018;44(5):419-34. doi: 10.1177/0145721718794879 [published Online First: 2018/08/28]

128. Arias-Colmenero T, Pérez-Morente M, Ramos-Morcillo AJ, et al. Experiences and Attitudes of People with HIV/AIDS: A Systematic Review of Qualitative Studies. *Int J Environ Res Public Health* 2020;17(2) doi: 10.3390/ijerph17020639 [published Online First: 2020/01/23]

129. Brömdal A, Mullens AB, Phillips TM, et al. Experiences of transgender prisoners and their knowledge, attitudes, and practices regarding sexual behaviors and HIV/STIs: A systematic review. *Int J Transgend* 2019;20(1):4-20. doi: 10.1080/15532739.2018.1538838 [published Online First: 2018/11/22]

130. Chi PL, Li XM. Impact of Parental HIV/AIDS on Children's Psychological Well-Being: A Systematic Review of Global Literature. *Aids and Behavior* 2013;17(7):2554-74. doi: 10.1007/s10461-012-0290-2

131. Eshun-Wilson I, Rohwer A, Hendricks L, et al. Being HIV positive and staying on antiretroviral therapy in Africa: A qualitative systematic review and theoretical model. *PLoS One* 2019;14(1):e0210408. doi: 10.1371/journal.pone.0210408 [published Online First: 2019/01/11]

132. Gannon BN, Stacciarini J-MR. Review of the literature: A rural–urban comparison of social networks of older adults living with HIV. *JANAC: Journal of the Association of Nurses in AIDS Care* 2016;27(4):419-29. doi: http://dx.doi.org/10.1016/j.jana.2016.02.004

133. Goldberg RE, Short SE. What do we know about children living with HIV-infected or AIDS-ill adults in Sub-Saharan Africa? A systematic review of the literature. *AIDS Care* 2016;28 Suppl 2(sup2):130-41. doi: 10.1080/09540121.2016.1176684 [published Online First: 2016/07/09]

134. Hainsworth EG, Shahmanesh M, Stevenson F. Exploring the views and experiences of HIV positive patients treated for cancer: a systematic review of the literature. *AIDS Care* 2018;30(5):535-43. doi: 10.1080/09540121.2017.1381332 [published Online First: 2017/10/05]

135. Kimera E, Vindevogel S, De Maeyer J, et al. Challenges and support for quality of life of youths living with HIV/AIDS in schools and larger community in East Africa: a systematic review. *Syst Rev* 2019;8(1):64. doi: 10.1186/s13643-019-0980-1 [published Online First: 2019/02/28]

136. Lowther K, Selman L, Harding R, et al. Experience of persistent psychological symptoms and perceived stigma among people with HIV on antiretroviral therapy (ART): a systematic review. *Int J Nurs Stud* 2014;51(8):1171-89. doi: 10.1016/j.ijnurstu.2014.01.015 [published Online First: 2014/03/08]

137. McInerney P, Brysiewicz P. A systematic review of the experiences of caregivers in providing home-based care to persons with HIV/AIDS in Africa. *JBI Libr Syst Rev* 2009;7(4):130-53. doi: 10.11124/01938924-200907040-00001 [published Online First: 2009/01/01]

138. Nattabi B, Li J, Thompson SC, et al. A systematic review of factors influencing fertility desires and intentions among people living with HIV/AIDS: implications for policy and service delivery. *AIDS Behav* 2009;13(5):949-68. doi: 10.1007/s10461-009-9537-y [published Online First: 2009/03/31]

For peer review only

139. Roger KS, Mignone J, Kirkland S. Social aspects of HIV/AIDS and aging: a thematic review. *Can J Aging* 2013;32(3):298-306. doi: 10.1017/s0714980813000330 [published Online First: 2013/08/15]

140. Rubtsova AA, Kempf MC, Taylor TN, et al. Healthy Aging in Older Women Living with HIV Infection: a Systematic Review of Psychosocial Factors. *Curr HIV/AIDS Rep* 2017;14(1):17-30. doi: 10.1007/s11904-017-0347-y [published Online First: 2017/02/15]

141. Zeglin RJ, Hergenrather KC, Garcia JH. A Systematic Review of the Common Experiences of Caregivers of HIV plus Men Who Have Sex with Men: Implications for Counselors. *J Rehabil* 2018;84(1):22-30.

142. Baral S, Holland CE, Shannon K, et al. Enhancing benefits or increasing harms: community responses for HIV among men who have sex with men, transgender women, female sex workers, and people who inject drugs. *J Acquir Immune Defic Syndr* 2014;66 Suppl 3:S319-28. doi: 10.1097/qai.0000000000000233 [published Online First: 2014/07/10]

143. Beres LK, Narasimhan M, Robinson J, et al. Non-specialist psychosocial support interventions for women living with HIV: A systematic review. *AIDS Care* 2017;29(9):1079-87. doi: 10.1080/09540121.2017.1317324 [published Online First: 2017/04/26]

144. Campbell C, Skovdal M, Gibbs A. Creating social spaces to tackle AIDS-related stigma: reviewing the role of church groups in Sub-Saharan Africa. *AIDS Behav* 2011;15(6):1204-19. doi: 10.1007/s10461-010-9766-0 [published Online First: 2010/07/30]

145. Han HR, Kim K, Murphy J, et al. Community health worker interventions to promote psychosocial outcomes among people living with HIV-A systematic review. *PLoS One* 2018;13(4):e0194928. doi: 10.1371/journal.pone.0194928 [published Online First: 2018/04/25]

146. Kaufman ZA, Spencer TS, Ross DA. Effectiveness of sport-based HIV prevention interventions: a systematic review of the evidence. *AIDS Behav* 2013;17(3):987-1001. doi: 10.1007/s10461-012-0348-1 [published Online First: 2012/10/26]

147. Kerrigan D, Kennedy CE, Morgan-Thomas R, et al. A community empowerment approach to the HIV response among sex workers: effectiveness, challenges, and considerations for implementation and scale-up. *Lancet* 2015;385(9963):172-85. doi: 10.1016/s0140-6736(14)60973-9 [published Online First: 2014/07/26]

148. Mak WWS, Mo PKH, Ma GYK, et al. Meta-analysis and systematic review of studies on the effectiveness of HIV stigma reduction programs. *Soc Sci Med* 2017;188:30-40. doi: 10.1016/j.socscimed.2017.06.045 [published Online First: 2017/07/14]

149. Rao D, Elshafei A, Nguyen M, et al. A systematic review of multi-level stigma interventions: state of the science and future directions. *BMC Med* 2019;17(1):41. doi: 10.1186/s12916-018-1244-y [published Online First: 2019/02/17]

150. Sengupta S, Banks B, Jonas D, et al. HIV interventions to reduce HIV/AIDS stigma: a systematic review. *AIDS Behav* 2011;15(6):1075-87. doi: 10.1007/s10461-010-9847-0 [published Online First: 2010/11/23]

151. Skevington SM, Sovetkina EC, Gillison FB. A systematic review to quantitatively evaluate 'Stepping Stones': a participatory community-based HIV/AIDS prevention intervention. *AIDS Behav* 2013;17(3):1025-39. doi: 10.1007/s10461-012-0327-6 [published Online First: 2012/11/07]

152. Stangl AL, Lloyd JK, Brady LM, et al. A systematic review of interventions to reduce HIV-related stigma and discrimination from 2002 to 2013: how far have we come? *J Int AIDS Soc* 2013;16(3 Suppl 2):18734. doi: 10.7448/ias.16.3.18734

18734 [published Online First: 2013/11/28]

For peer review only

153. Williams MV, Palar K, Derose KP. Congregation-based programs to address HIV/AIDS: elements of successful implementation. *J Urban Health* 2011;88(3):517-32. doi: 10.1007/s11524-010-9526-5 [published Online First: 2011/02/19]

154. Aderomilehin O, Hanciles-Amu A, Ozoya OO. Perspectives and Practice of HIV Disclosure to Children and Adolescents by Health-Care Providers and Caregivers in sub-Saharan Africa: A Systematic Review. *Front Public Health* 2016;4:166. doi: 10.3389/fpubh.2016.00166 [published Online First: 2016/08/30]

155. Cao WN, Wong HM, Chang C, et al. Behavioral interventions promoting HIV serostatus disclosure to sex partners among HIV-positive men who have sex with men: a systematic review. *Int J Public Health* 2019;64(7):985-98. doi: 10.1007/s00038-019-01275-4

156. Kennedy CE, Haberlen S, Amin A, et al. Safer disclosure of HIV serostatus for women living with HIV who experience or fear violence: a systematic review. *J Int AIDS Soc* 2015;18(Suppl 5):20292. doi: 10.7448/ias.18.6.20292

20292 [published Online First: 2015/12/09]

157. Krauss BJ, Letteney S, Okoro CN. Why Tell Children: A Synthesis of the Global Literature on Reasons for Disclosing or Not Disclosing an HIV Diagnosis to Children 12 and under. *Front Public Health* 2016;4:13. doi: 10.3389/fpubh.2016.00181

158. Qiao S, Li XM, Stanton B. Disclosure of Parental HIV Infection to Children: A Systematic Review of Global Literature. *Aids and Behavior* 2013;17(1):369-89. doi: 10.1007/s10461-011-0069-x

159. Tam M, Amzel A, Phelps BR. Disclosure of HIV serostatus among pregnant and postpartum women in sub-Saharan Africa: a systematic review. *AIDS Care* 2015;27(4):436-50. doi: 10.1080/09540121.2014.997662 [published Online First: 2015/01/31]

160. Vreeman RC, Gramelspacher AM, Gisore PO, et al. Disclosure of HIV status to children in resource-limited settings: a systematic review. *J Int AIDS Soc* 2013;16(1):18466. doi: 10.7448/ias.16.1.18466

18466 [published Online First: 2013/05/30]

161. Whembolua GL, Conserve DF, Thomas K, et al. A Systematic Review of HIV Serostatus Disclosure Among African Immigrants in Europe. *J Immigr Minor Health* 2017;19(4):947-58. doi: 10.1007/s10903-016-0456-5 [published Online First: 2016/07/09]

162. Wilkinson B, Arora KS. A Systematic Review on Confidentiality, Disclosure, and Stigma in the United States: Lessons for HIV Care in Pregnancy From Reproductive Genetics. *New Bioeth* 2015;21(2):142-54. doi: 10.1179/2050287715z.00000000067 [published Online First: 2015/01/01]

163. Cheema E, Abbas A, Al-Hamid A. Healthcare-related factors affecting the management of HIV infected patients: a systematic review of qualitative evidence. *Int J STD AIDS* 2019;30(14):1350-61. doi: 10.1177/0956462419875357 [published Online First: 2019/11/20]

164. Feyissa GT, Lockwood C, Woldie M, et al. Reducing HIV-related stigma and discrimination in healthcare settings: a systematic review of guidelines, tools, standards of practice, best practices, consensus statements and systematic reviews. *J Multidiscip Healthc* 2018;11:405-16. doi: 10.2147/jmdh.S170720 [published Online First: 2018/09/15]

165. Feyissa GT, Lockwood C, Woldie M, et al. Reducing HIV-related stigma and discrimination in healthcare settings: A systematic review of quantitative evidence. *PLoS One* 2019;14(1):e0211298. doi: 10.1371/journal.pone.0211298 [published Online First: 2019/01/27]

166. Geter A, Herron AR, Sutton MY. HIV-Related Stigma by Healthcare Providers in the United States: A Systematic Review. *AIDS Patient Care STDS* 2018;32(10):418-24. doi: 10.1089/apc.2018.0114 [published Online First: 2018/10/03]

For peer review only

167. Kaladharan S, Daken K, Mullens AB, et al. Tools to measure HIV knowledge, attitudes & practices (KAPs) in healthcare providers: a systematic review. *AIDS Care* 2020:1-7. doi: 10.1080/09540121.2020.1822502 [published Online First: 2020/09/24]

168. Nyblade L, Stockton MA, Giger K, et al. Stigma in health facilities: why it matters and how we can change it. *BMC Med* 2019;17(1):25. doi: 10.1186/s12916-019-1256-2 [published Online First: 2019/02/16]

169. Phillips JC, Caine V, Dewart G, et al. Teaching HIV-specific content for pre-licensure nursing and health professions students: a review and synthesis. *Aids Care-Psychol Socio-Med Asp Aids-Hiv* 2018;30(12):1614-21. doi: 10.1080/09540121.2018.1510108

170. Pleuhs B, Quinn KG, Walsh JL, et al. Health Care Provider Barriers to HIV Pre-Exposure Prophylaxis in the United States: A Systematic Review. *AIDS Patient Care STDS* 2020;34(3):111-23. doi: 10.1089/apc.2019.0189 [published Online First: 2020/02/29]

171. Anderson JE, Kanters S. Lack of Sexual Minorities' Rights as a Barrier to HIV Prevention Among Men Who Have Sex with Men and Transgender Women in Asia: A Systematic Review. *Lgbt Health* 2015;2(1):16-26. doi: 10.1089/lgbt.2014.0024

172. Juliastuti D, Dean J, Fitzgerald L. Sexual and reproductive health of women living with HIV in Muslim-majority countries: a systematic mixed studies review. *BMC Int Health Hum Rights* 2020;20(1):5. doi: 10.1186/s12914-020-00225-z [published Online First: 2020/03/13]

173. Jürgens R, Csete J, Amon JJ, et al. People who use drugs, HIV, and human rights. *The Lancet* 2010;376(9739):475-85. doi: http://dx.doi.org/10.1016/S0140-6736(10)60830-6

174. Malta M, Ralil da Costa M, Bastos FI. The paradigm of universal access to HIV-treatment and human rights violation: how do we treat HIV-positive people who use drugs? *Curr HIV/AIDS Rep* 2014;11(1):52-62. doi: 10.1007/s11904-013-0196-2 [published Online First: 2013/12/27]

175. Oldenburg CE, Perez-Brumer AG, Reisner SL, et al. Human rights protections and HIV prevalence among MSM who sell sex: Cross-country comparisons from a systematic review and meta-analysis. *Global Public Health* 2018;13(4):414-25. doi: 10.1080/17441692.2016.1149598

176. Stangl AL, Singh D, Windle M, et al. A systematic review of selected human rights programs to improve HIV-related outcomes from 2003 to 2015: what do we know? *BMC Infect Dis* 2019;19(1):209. doi: 10.1186/s12879-019-3692-1 [published Online First: 2019/03/06]

177. Wagener MN, Roelofs PDDM, Miedema HS, et al. The development of a multidisciplinary, evidence-based guideline for “HIV and employment”. *AIDS Care* 2015;27(2):133-41. doi: http://dx.doi.org/10.1080/09540121.2014.952612

178. Chambers LA, Wilson MG, Rueda S, et al. Evidence informing the intersection of HIV, aging and health: A scoping review. *AIDS and Behavior* 2014;18(4):661-75. doi: http://dx.doi.org/10.1007/s10461-013-0627-5

179. Ingram L, Stafford C, Deming ME, et al. A Systematic Mixed Studies Review of the Intersections of Social-Ecological Factors and HIV Stigma in People Living With HIV in the U.S. South. *J Assoc Nurses AIDS Care* 2019;30(3):330-43. doi: 10.1097/jnc.0000000000000076 [published Online First: 2019/04/26]

180. Jackson-Best F, Edwards N. Stigma and intersectionality: a systematic review of systematic reviews across HIV/AIDS, mental illness, and physical disability. *BMC Public Health* 2018;18(1):919. doi: 10.1186/s12889-018-5861-3 [published Online First: 2018/07/28]

181. Monteiro SS, Villela WV, Soares PS. The interaction between axes of inequality in studies on discrimination, stigma and HIV/AIDS: contributions to the recent international literature. *Glob Public Health* 2013;8(5):519-33. doi: 10.1080/17441692.2013.779738 [published Online First: 2013/04/05]

For peer review only

182. Smith SC. The intersection of race, military status, and disability: A systematic review of marginalization and self-esteem being a mediator against negative life outcomes for HIV positive African American male veterans [Psy.D.]. Azusa Pacific University, 2019.

183. Wilson PA, Valera P, Martos AJ, et al. Contributions of Qualitative Research in Informing HIV/AIDS Interventions Targeting Black MSM in the United States. *J Sex Res* 2016;53(6):642-54. doi: 10.1080/00224499.2015.1016139 [published Online First: 2015/08/05]

184. Alexandra Marshall S, Brewington KM, Kathryn Allison M, et al. Measuring HIV-related stigma among healthcare providers: a systematic review. *AIDS Care* 2017;29(11):1337-45. doi: 10.1080/09540121.2017.1338654 [published Online First: 2017/06/11]

185. Earnshaw VA, Chaudoir SR. From conceptualizing to measuring HIV stigma: a review of HIV stigma mechanism measures. *AIDS Behav* 2009;13(6):1160-77. doi: 10.1007/s10461-009-9593-3 [published Online First: 2009/07/29]

186. Fitzgerald-Husek A, Van Wert MJ, Ewing WF, et al. Measuring stigma affecting sex workers (SW) and men who have sex with men (MSM): A systematic review. *PLoS One* 2017;12(11):e0188393. doi: 10.1371/journal.pone.0188393 [published Online First: 2017/12/01]

187. Kipp AM, Haley C, Mukolo A, et al. GAPS IN MEASURING AND UNDERSTANDING THE EFFECT OF HIV STIGMA ON HEALTH OUTCOMES: INSIGHT FROM A SYSTEMATIC REVIEW OF HIV STIGMA AND ART ADHERENCE. *Ann Behav Med* 2012;43:S59-S59.

188. Mahajan AP, Sayles JN, Patel VA, et al. Stigma in the HIV/AIDS epidemic: A review of the literature and recommendations for the way forward. *AIDS* 2008;22(Suppl2):S67-S79. doi: http://dx.doi.org/10.1097/01.aids.0000327438.13291.62

189. McAteer CI, Truong NT, Aluoch J, et al. A systematic review of measures of HIV/AIDS stigma in paediatric HIV-infected and HIV-affected populations. *J Int AIDS Soc* 2016;19(1):21204. doi: 10.7448/ias.19.1.21204

21204 [published Online First: 2016/10/09]

190. Dunbar W, Labat A, Raccurt C, et al. A realist systematic review of stigma reduction interventions for HIV prevention and care continuum outcomes among men who have sex with men. *Int J STD AIDS* 2020;31(8):712-23. doi: 10.1177/0956462420924984 [published Online First: 2020/07/08]

191. Glick JL, Russo R, Jivapong B, et al. The PrEP Care Continuum Among Cisgender Women Who Sell Sex and/or Use Drugs Globally: A Systematic Review. *AIDS Behav* 2020;24(5):1312-33. doi: 10.1007/s10461-019-02733-z [published Online First: 2019/12/07]

192. Liu X, Erasmus V, Wu Q, et al. Behavioral and psychosocial interventions for HIV prevention in floating populations in China over the past decade: a systematic literature review and meta-analysis. *PLoS One* 2014;9(6):e101006. doi: 10.1371/journal.pone.0101006 [published Online First: 2014/06/26]

193. Poteat T, Wirtz AL, Radix A, et al. HIV risk and preventive interventions in transgender women sex workers. *The Lancet* 2015;385(9964):274-86. doi: http://dx.doi.org/10.1016/S0140-6736(14)60833-3

194. Chen D, Luo G, Meng X, et al. Efficacy of HIV interventions among factory workers in low- and middle-income countries: a systematic review. *BMC Public Health* 2020;20(1):1310. doi: 10.1186/s12889-020-09333-w [published Online First: 2020/08/30]

195. Hartog K, Hubbard CD, Krouwer AF, et al. Stigma reduction interventions for children and adolescents in low- and middle-income countries: Systematic review of intervention strategies. *Soc Sci Med* 2020;246:112749. doi: 10.1016/j.socscimed.2019.112749 [published Online First: 2020/01/25]

For peer review only

196. Kemp CG, Jarrett BA, Kwon CS, et al. Implementation science and stigma reduction interventions in low- and middle-income countries: a systematic review. *BMC Med* 2019;17(1):6. doi: 10.1186/s12916-018-1237-x [published Online First: 2019/02/16]

197. Lofgren SM, Nakasujja N, Boulware DR. Systematic Review of Interventions for Depression for People Living with HIV in Africa. *Aids and Behavior* 2018;22(1):1-8. doi: 10.1007/s10461-017-1906-3

198. Ma PHX, Chan ZCY, Loke AY. Self-Stigma Reduction Interventions for People Living with HIV/AIDS and Their Families: A Systematic Review. *AIDS Behav* 2019;23(3):707-41. doi: 10.1007/s10461-018-2304-1 [published Online First: 2018/10/10]

199. Pantelic M, Sprague L, Stangl AL. It's not "all in your head": critical knowledge gaps on internalized HIV stigma and a call for integrating social and structural conceptualizations. *BMC Infect Dis* 2019;19(1):210. doi: 10.1186/s12879-019-3704-1 [published Online First: 2019/03/06]

200. Joseph Davey D, West S, Umutoni V, et al. A Systematic Review of the Current Status of Safer Conception Strategies for HIV Affected Heterosexual Couples in Sub-Saharan Africa. *AIDS Behav* 2018;22(9):2916-46. doi: 10.1007/s10461-018-2170-x [published Online First: 2018/06/06]

201. Taggart T, Grewe ME, Conserve DF, et al. Social Media and HIV: A Systematic Review of Uses of Social Media in HIV Communication. *J Med Internet Res* 2015;17(11):e248. doi: 10.2196/jmir.4387 [published Online First: 2015/11/04]

202. International Center for Research on Women (ICRW), STRIVE. A global HIV stigma reduction framework adapted and implemented in five settings in India: Summary report, 2013.

203. Stangl A, Brady L, Fritz K. STRIVE Technical Brief: Measuring HIV stigma and discrimination. Washington, D.C.: International Center for Research on Women, 2012 (Updated in 2018):1-7.

204. UNAIDS. Key Populations [cited Protocol refs. Available from: https://www.unaids.org/en/topic/key-populations accessed January 12, 2021.

205. Global Network Of People Living with HIV (GNP+). The People Living with HIV Stigma Index [Available from: https://www.stigmaindex.org/ accessed February 22, 2021.

206. Schneberk T, Bolshakova M, Sloan K, et al. Quality Indicators for High Need Patients: Systematic Review Protocol. PROSPERO registration: CRD42020215917. November 29, 2020

207. De Roo ML, Leemans K, Claessen SJ, et al. Quality indicators for palliative care: update of a systematic review. *J Pain Symptom Manage* 2013;46(4):556-72. doi: 10.1016/j.jpainsymman.2012.09.013 [published Online First: 2013/07/03]

208. Mokkink LB, Terwee CB, Patrick DL, et al. The COSMIN checklist for assessing the methodological quality of studies on measurement properties of health status measurement instruments: an international Delphi study. *Qual Life Res* 2010;19(4):539-49. doi: 10.1007/s11136-010-9606-8 [published Online First: 2010/02/20]

209. Sterne JA, Hernan MA, Reeves BC, et al. ROBINS-I: a tool for assessing risk of bias in non-randomised studies of interventions. *BMJ* 2016;355:i4919. doi: 10.1136/bmj.i4919 [published Online First: 2016/10/14]

210. Cochrane Methods Bias. RoB 2: A revised Cochrane risk-of-bias tool for randomized trials [cited Protocol refs. Available from: https://methods.cochrane.org/bias/resources/rob-2-revised-cochrane-risk-bias-tool-randomized-trials accessed January 12, 2020.

211. Rover C, Knapp G, Friede T. Hartung-Knapp-Sidik-Jonkman approach and its modification for random-effects meta-analysis with few studies. *BMC Med Res Methodol* 2015;15:99. doi: 10.1186/s12874-015-0091-1 [published Online First: 2015/11/18]

212. Thomas J, O'Mara-Eves A, Brunton G. Using qualitative comparative analysis (QCA) in systematic reviews of complex interventions: a worked example. *Syst Rev* 2014;3:67. doi: 10.1186/2046-4053-3-67 [published Online First: 2014/06/22]

213. GRADE working group. GRADE [cited Protocol refs. Available from: https://www.gradeworkinggroup.org/ accessed January 12, 2020.

COPYRIGHT/LICENSE FOR PUBLICATIION

The Corresponding Author has the right to grant on behalf of all authors and does grant on behalf of all authors, a worldwide licence to the Publishers and its licensees in perpetuity, in all forms, formats and media (whether known now or created in the future), to i) publish, reproduce, distribute, display and store the Contribution, ii) translate the Contribution into other languages, create adaptations, reprints, include within collections and create summaries, extracts and/or, abstracts of the Contribution, iii) create any other derivative work(s) based on the Contribution, iv) to exploit all subsidiary rights in the Contribution, v) the inclusion of electronic links from the Contribution to third party material where-ever it may be located; and, vi) licence any third party to do any or all of the above."

For peer review only

Figure 1: Analytic framework

For peer review only

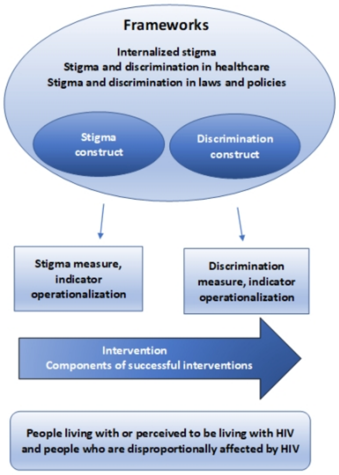

Analytic framework

855x481mm (38 x 38 DPI)

Appendix

For peer review only

Draft search strategies

PubMed
(frameworks)
Date: 1/11/2021
Yield: 112
Terms
("framework"[All Fields] OR "framework s"[All Fields] OR "frameworks"[All Fields] OR "logic model"[All Fields] OR "analytic model"[All Fields]) AND ("stigma"[Title] OR "discrimination"[Title]) AND ("hiv"[MeSH Terms] OR "hiv"[All Fields] OR ("acquired immunodeficiency syndrome"[MeSH Terms] OR ("acquired"[All Fields] AND "immunodeficiency"[All Fields] AND "syndrome"[All Fields]) OR "acquired immunodeficiency syndrome"[All Fields] OR "aids"[All Fields]))
OR
(measures)
Date: 1/11/2021
Yield: 112
Filter: 2008-date
Terms
("measure*"[Title] OR "test"[Title] OR ("scale s"[All Fields] OR "scaled"[All Fields] OR "scaling"[All Fields] OR "scalings"[All Fields] OR "weights and measures"[MeSH Terms] OR ("weights"[All Fields] AND "measures"[All Fields]) OR "weights and measures"[All Fields] OR "scale"[All Fields] OR "scales"[All Fields]) OR "tool"[All Fields] OR "assess*"[Title] OR ("psychometrical"[All Fields] OR "psychometrically"[All Fields] OR "psychometrics"[MeSH Terms] OR "psychometrics"[All Fields] OR "psychometric"[All Fields])) AND ("stigma"[Title] OR "discrimination"[Title]) AND ("hiv"[MeSH Terms] OR "hiv"[All Fields] OR ("acquired immunodeficiency syndrome"[MeSH Terms] OR ("acquired"[All Fields] AND "immunodeficiency"[All Fields] AND "syndrome"[All Fields]) OR "acquired immunodeficiency syndrome"[All Fields] OR "aids"[All Fields]))
OR
(interventions SR)
Date: 1/11/2021
Filter: Systematic reviews
Yield: 225
Terms
("social stigma"[MeSH Terms] OR ("social"[All Fields] AND "stigma"[All Fields]) OR "social stigma"[All Fields] OR "stigma"[All Fields] OR "stigmas"[All Fields] OR "stigma s"[All Fields] OR ("discriminabilities"[All Fields] OR "discriminability"[All Fields] OR "discriminable"[All Fields] OR "discriminably"[All Fields] OR "discriminance"[All Fields] OR "discriminant"[All Fields] OR "discriminants"[All Fields] OR "discriminate"[All Fields] OR "discriminated"[All Fields] OR "discriminates"[All Fields] OR "discriminating"[All Fields] OR "discrimination, psychological"[MeSH Terms] OR ("discrimination"[All Fields] AND "psychological"[All Fields]) OR "psychological discrimination"[All Fields] OR "discrimination"[All Fields] OR "discriminations"[All Fields] OR "discriminative"[All Fields] OR "discriminatively"[All Fields] OR "discriminator"[All Fields] OR "discriminators"[All Fields])) AND ("hiv"[MeSH Terms] OR "hiv"[All Fields] OR ("acquired immunodeficiency syndrome"[MeSH Terms] OR

For peer review only - http://bmjopen.bmj.com/site/about/guidelines.xhtml

("acquired"[All Fields] AND "immunodeficiency"[All Fields] AND "syndrome"[All Fields]) OR "acquired immunodeficiency syndrome"[All Fields] OR "aids"[All Fields]))
OR
(interventions RCTs)
Date: 2/5/2021
Yield: 199 results
Filters: Randomized Controlled Trial, English language, 2008 - 2021
Terms
("social stigma"[MeSH Terms] OR ("social"[All Fields] AND "stigma"[All Fields]) OR "social stigma"[All Fields] OR "stigma"[All Fields] OR "stigmas"[All Fields] OR "stigma s"[All Fields] OR ("discriminabilities"[All Fields] OR "discriminability"[All Fields] OR "discriminable"[All Fields] OR "discriminably"[All Fields] OR "discriminance"[All Fields] OR "discriminant"[All Fields] OR "discriminants"[All Fields] OR "discriminate"[All Fields] OR "discriminated"[All Fields] OR "discriminates"[All Fields] OR "discriminating"[All Fields] OR "discrimination, psychological"[MeSH Terms] OR ("discrimination"[All Fields] AND "psychological"[All Fields]) OR "psychological discrimination"[All Fields] OR "discrimination"[All Fields] OR "discriminations"[All Fields] OR "discriminative"[All Fields] OR "discriminatively"[All Fields] OR "discriminator"[All Fields] OR "discriminators"[All Fields]))
AND
("hiv"[MeSH Terms] OR "hiv"[All Fields] OR ("acquired immunodeficiency syndrome"[MeSH Terms] OR ("acquired"[All Fields] AND "immunodeficiency"[All Fields] AND "syndrome"[All Fields]) OR "acquired immunodeficiency syndrome"[All Fields] OR "aids"[All Fields]))
OR
(systematic reviews)
Date: 5/6/2021
Yield: 235
Publication Date Range: 2008-05/06/2021
Filter: Systematic Review
Terms
Social stigma[MESH terms] OR stigma OR discrimination OR discrimination, psychological[MESH terms]
AND
HIV[Mesh terms] OR hiv OR acquired immunodeficiency syndrome[MESH terms] OR "acquired immunodeficiency syndrome" OR "AIDS"[Title/Abstract]

PsycINFO
(Frameworks; limited ‘framework’ terms to title field, 2008-2021 publication date range)
Date: 3/26/2021
Yield: 41
Terms
(ti(stigma OR discrimin*) OR ab(stigma OR discrimin*) OR su(stigma OR discrimin*)) AND (ti(HIV OR "acquired immunodeficiency syndrome") OR ab(HIV OR "acquired immunodeficiency syndrome") OR su(HIV OR "acquired immunodeficiency syndrome")) AND ti(framework OR frameworks OR "logic model" OR "analytic model") AND la.exact("ENG")
OR
(Measures; limited ‘measures’ terms to title field)

For peer review only - http://bmjopen.bmj.com/site/about/guidelines.xhtml

Date: 3/26/2021
Yield: 116
Terms
(ti(stigma OR discrimin*) OR ab(stigma OR discrimin*) OR su(stigma OR discrimin*)) AND (ti(HIV OR "acquired immunodeficiency syndrome") OR ab(HIV OR "acquired immunodeficiency syndrome") OR su(HIV OR "acquired immunodeficiency syndrome")) AND ti(measure OR measures OR scale OR scales OR tool OR tools) AND (la.exact("ENG") AND pd(20080101-20211231))
OR
Date: 3/19/2021
Yield: 58
Terms
((ti(stigma OR discrimin*) OR ab(stigma OR discrimin*) OR su(stigma OR discrimin*)) AND (ti(HIV OR "acquired immunodeficiency syndrome") OR ab(HIV OR "acquired immunodeficiency syndrome") OR su(HIV OR "acquired immunodeficiency syndrome")))
AND
(la.exact("ENG") AND me.exact("Prospective Study" OR "Clinical Trial")
AND
pd(20080101-20211231))
OR
Date: 5/6/2021
Yield: 100
Publication Date Range: After January 1, 2008
Methodology: Systematic Review
Terms
noft(stigma OR discriminat*) AND noft("hiv" OR "acquired immunodeficiency syndrome" OR "AIDS")

Web of Science
(Framework; omitted conference proceedings, added Research Areas, limited ‘framework’ terms to title field, 2008-2021 publication date range)
Yield: 14
Terms
(TS=(stigma OR discrim*) AND TS=(HIV OR "acquired immunodeficiency syndrome") AND TI=(framework OR frameworks OR "logic model" OR "logic models" OR "analytic model" OR "analytic models")) AND LANGUAGE: (English)
Refined by: RESEARCH AREAS: (HEALTH CARE SCIENCES SERVICES OR PSYCHOLOGY)
Indexes=SCI-EXPANDED, SSCI, BKCI-S, BKCI-SSH, ESCI Timespan=1900-2021
OR
(Measures; omitted conference proceedings, added Research Areas, limited ‘measures’ terms to title field)
Yield: 75
TS=(stigma OR discrim*) AND TS=(HIV OR "acquired immunodeficiency syndrome") AND TI=(measure OR measures OR scale OR scales OR tool OR tools)) AND LANGUAGE: (English)

For peer review only - http://bmjopen.bmj.com/site/about/guidelines.xhtml

Refined by: RESEARCH AREAS: (PSYCHOLOGY OR HEALTH CARE SCIENCES SERVICES)
Indexes=SCI-EXPANDED, SSCI, BKCI-S, BKCI-SSH, ESCI Timespan=2008-2021
OR
Interventions (omitted conference proceedings, added Research Areas, limited 'RCT' terms to topic field)
Yield: 76
(TS=(stigma OR discrim*) AND TS=(HIV OR "acquired immunodeficiency syndrome") AND TS=("randomized controlled trial" OR "randomized controlled trials" OR RCT OR RCTs OR "intervention study" OR "intervention studies")) AND LANGUAGE: (English)
Refined by: RESEARCH AREAS: (PSYCHOLOGY OR HEALTH CARE SCIENCES SERVICES)
Indexes=SCI-EXPANDED, SSCI, BKCI-S, BKCI-SSH, ESCI Timespan=2008-2021
OR
Date: 5/6/2021
Yield: 263
Publication Date Range: 2008-2021
Terms
Indexes=SCI-EXPANDED, SSCI, A&HCI, CPCI-S, CPCI-SSH, BKCI-S, BKCI-SSH, ESCI, CCR-EXPANDED
Search Strategy
TOPIC: (stigma OR discriminat*) AND TOPIC: ("hiv" OR "acquired immunodeficiency syndrome" OR "AIDS") AND TOPIC: ("systematic review")

Campbell Collaboration
Date: 1/19/2021
Yield: 81
Campbell Systematic Reviews
Terms
stigma* OR discriminat*" and "HIV OR AIDS OR "acquired immunodeficiency"
Research evidence by topic
(stigma OR discrimination)
(HIV OR AIDS)

Cochrane Database of Systematic Reviews
Date: 1/29/2021
Yield: 6
Terms
(stigma OR discrimination):ti,ab,kw (Word variations have been searched)
AND
MeSH descriptor: [HIV] explode all trees OR MeSH descriptor: [Acquired Immunodeficiency Syndrome] 5 tree(s) exploded OR HIV OR "acquired immunodeficiency syndrome" OR "acquired immunodeficiency syndrome"):ti,ab,kw (Word variations have been searched)

PROSPERO
Date: 1/19/2021

For peer review only - http://bmjopen.bmj.com/site/about/guidelines.xhtml
For peer review only

Yield: 173
Terms
(stigma or discrimin*)
AND
MeSH DESCRIPTOR HIV EXPLODE ALL TREES OR MeSH DESCRIPTOR Acquired Immunodeficiency Syndrome EXPLODE ALL OR "acquired immunodeficiency syndrome" OR "acquired immunodeficiency" OR HIV

Open Science Framework
Date: 1/27/2021
Yield: 4 relevant projects
Terms: (stigma OR discrimination) AND (HIV OR AIDS)

Universal Human Rights Index
Date: 1/27/2021
Platform: https://www.ohchr.org/EN/Issues/HIV/Pages/Documents.aspx
Yield: 2 relevant documents
Terms: “Persons living with HIV/AIDS” AND (stigma OR discrimination)

PAIS
Date: 4/9/2021
Yield: 9
Terms: NOFT(hive and stigma and law and intervention)

International AIDS Society (IAS)
Date: 1/27/2021
Yield: 5 relevant documents
Terms: (stigma OR discrimination) AND (HIV OR AIDS)

Joint United Nations Programme on HIV/AIDS (UNAIDS)
Date: 1/27/2021
Yield: 15 relevant documents
Terms: (stigma OR discrimination) AND (HIV OR AIDS)

United Nations Development Programme (UNDP)
Date: 4/13/2021
Yield: 6 relevant documents
Terms: (stigma OR discrimination) AND (HIV OR AIDS)

STRIVE:
Date: 1/27/2021
Yield: 2 relevant documents
Terms: (stigma OR discrimination) AND (HIV OR AIDS)

Health Policy Plus
Date: 1/27/2021

For peer review only

For peer review only - http://bmjopen.bmj.com/site/about/guidelines.xhtml

Yield: 5 relevant documents
Terms: (stigma OR discrimination) AND (HIV OR AIDS)

The Center for HIV Law and Policy
https://www.hivlawandpolicy.org/
Date: 4/19/2021
Yield: 15 relevant documents
Terms: topic: “stigma”

The Global Fund
https://www.theglobalfund.org/en/funding-model/throughout-the-cycle/community-rights-gender/
Date: 4/13/2021
Yield: 20 relevant documents
Terms: “Human rights”

HIV Legal Network
http://www.hivlegalnetwork.ca/site/?lang=en
Date: 4/16/2021
Publications/Find Publications/ keyword search:
Yield: 11 relevant documents
Terms: stigma

Sage: A resource-sharing community for Canadian HIV and hepatitis C service providers
http://sagecollection.ca/en
Limited publication type: Reports
Date: 4/16/2021
Yield: 3 potentially relevant documents
Terms: STIGMA AND (LAW OR LEGAL)

HeinOnline
Date: 3/19/2021
Yield: 272
Terms
(stigma OR discrmin*) AND (HIV OR "acquired immunodeficiency syndrome") AND (framework OR frameworks OR "logic model" OR "logic models" OR "analytic model" OR "analytic models") Topic: AIDS AND Timespan: 2008-2021
OR (stigma OR discrmin*) AND (HIV OR "acquired immunodeficiency syndrome") AND (measure OR measures OR scale OR scales OR asses* OR tool OR tools) AND Topic: AIDS AND Timespan: 2008-2021
OR
(stigma OR discrmin*) AND (HIV OR "acquired immunodeficiency syndrome") AND ("randomized controlled trial" OR "randomized controlled trials" OR "RCT" OR "RCTs") Topic: AIDS AND Timespan: 2008-2021
OR

For peer review only - http://bmjopen.bmj.com/site/about/guidelines.xhtml

(stigma OR discrmin*) AND (HIV OR "acquired immunodeficiency syndrome") AND ("systematic review" OR "systematic reviews") Topic: AIDS AND Timespan: 2008-2021

LegalTrac
Date: 5/12/2021
Yield: 78
Terms
keyword search - ("acquired immunodeficiency syndrome" OR HIV) AND (stigma OR discriminat*)
AND
Publication Date Range: 2008 - present
AND
Document Type: Article

For peer review only

For peer review only - http://bmjopen.bmj.com/site/about/guidelines.xhtml

Draft Evidence Table KQ1 Stigma and Discrimination Framework Descriptions

**Framework title**<br>**Author group / publisher**	**Scope and Aim/Purpose**<br>**Target**	**Stigma subtype, definition**<br>**Discrimination subtype, definition**	**Components**	**Summary**
Author, year<br>**Framework name:**<br>**Framework addressing:**<br>**Subdomain:**<br>**Specific to HIV:**	**Scope:**<br>**Aim/Purpose:**<br>**Target:**<br>**Framework HIV target:**	**Subtype:**<br>**Definitions:**		
Author, year<br>**Framework name:**<br>**Framework addressing:**<br>**Subdomain:**<br>**Specific to HIV:**	**Scope:**<br>**Aim/Purpose:**<br>**Target:**<br>**Framework HIV target:**	**Subtype:**<br>**Definitions:**		

Draft Evidence Table KQ2 Stigma and Discrimination Measure Descriptions

**Author group, publication year**<br>**Measure name**	**Stigma / discrimination subtype**<br>**Definition**<br>**Framework**	**Target population**<br>**Surveyed participants**	**Scale structure**<br>**Number of items**<br>**Answer mode**	**Reliability**<br>**Validity**
Author, year<br>**Author group/publisher:**<br>**Measure name:**<br>**Subdomain:**<br>**Specific to HIV:**	**Subtype**<br>**Definition:**<br>**Framework:**	**Target population:**<br>**Surveyed participants:**	**Scale structure:**<br>**Number of items:**<br>**Answer mode:**	**Reliability:**<br>**Validity**:
Author, year<br>**Author group/publisher:**<br>**Measure name:**<br>**Subdomain:**<br>**Specific to HIV:**	**Subtype**<br>**Definition:**<br>**Framework:**	**Target population:**<br>**Surveyed participants:**	**Scale structure:**<br>**Number of items:**<br>**Answer mode:**	**Reliability:**<br>**Validity**:

For peer review only - http://bmjopen.bmj.com/site/about/guidelines.xhtml

Draft Evidence Table KQ3 Stigma and Discrimination Intervention Evaluations

Study ID, PY<br>Country<br>Study design<br>Sample size	Participant details<br>Context / setting	Intervention type<br>Intervention description	Measure and effectiveness summary<br>Measure and unintended consequences
Author, year<br>**Country:**<br>**Study design:**<br>**Sample size:**<br>**Subdomain:**<br>**Specific to HIV:**	**Participant details:**<br>**Context / setting:**	**Intervention type:**<br>**Intervention description:**	**Measure and effectiveness summary:**<br>**Measure and unintended consequences:**

For peer review only

For peer review only - http://bmjopen.bmj.com/site/about/guidelines.xhtml

Draft Component Table KQ4a Stigma and Discrimination Intervention 'Critical Factors for Success'

Type of Intervention	Context	Component 1	Component 2	Component 3	Component 4	Component 5	Component 6	Component 7	Component 8
Intervention 1				√			√	√	√
Intervention 2			√				√		
Intervention 3		√				√	√		

Draft Component Table KQ4b Stigma and Discrimination Intervention 'Critical Factors for Failure'

Type of Intervention	Context	Component 1	Component 2	Component 3	Component 4	Component 5	Component 6	Component 7	Component 8
Intervention 1				√			√	√	√
Intervention 2			√				√		
Intervention 3		√				√	√		

For peer review only - http://bmjopen.bmj.com/site/about/guidelines.xhtml

For peer review only

PRISMA-P (Preferred Reporting Items for Systematic review and Meta-Analysis Protocols) 2015 checklist: recommended items to address in a systematic review protocol*

Section and topic	Page number	Item No	Checklist item
**ADMINISTRATIVE INFORMATION**			
Title:	1		
Identification	1	1a	Identify the report as a protocol of a systematic review
Update	N/A	1b	If the protocol is for an update of a previous systematic review, identify as such
Registration	2	2	If registered, provide the name of the registry (such as PROSPERO) and registration number
Authors:	1		
Contact	1	3a	Provide name, institutional affiliation, e-mail address of all protocol authors; provide physical mailing address of corresponding author
Contributions	20	3b	Describe contributions of protocol authors and identify the guarantor of the review
Amendments	N/A	4	If the protocol represents an amendment of a previously completed or published protocol, identify as such and list changes; otherwise, state plan for documenting important protocol amendments
Support:	20		
Sources	20	5a	Indicate sources of financial or other support for the review
Sponsor	20	5b	Provide name for the review funder and/or sponsor
Role of sponsor or funder	20	5c	Describe roles of funder(s), sponsor(s), and/or institution(s), if any, in developing the protocol
**INTRODUCTION**			
Rationale	3	6	Describe the rationale for the review in the context of what is already known
Objectives	3	7	Provide an explicit statement of the question(s) the review will address with reference to participants, interventions, comparators, and outcomes (PICO)
**METHODS**			
Eligibility criteria	5	8	Specify the study characteristics (such as PICO, study design, setting, time frame) and report characteristics (such as years considered, language, publication status) to be used as criteria for eligibility for the review
Information sources	5	9	Describe all intended information sources (such as electronic databases, contact with study authors, trial registers or other grey literature sources) with planned dates of coverage
Search strategy	Appendix	10	Present draft of search strategy to be used for at least one electronic database, including planned limits, such that it could be repeated
Study records:			

For peer review only

Data management	5	11a	Describe the mechanism(s) that will be used to manage records and data throughout the review
Selection process	5	11b	State the process that will be used for selecting studies (such as two independent reviewers) through each phase of the review (that is, screening, eligibility and inclusion in meta-analysis)
Data collection process	6	11c	Describe planned method of extracting data from reports (such as piloting forms, done independently, in duplicate), any processes for obtaining and confirming data from investigators
Data items	6	12	List and define all variables for which data will be sought (such as PICO items, funding sources), any pre-planned data assumptions and simplifications
Outcomes and prioritization	6	13	List and define all outcomes for which data will be sought, including prioritization of main and additional outcomes, with rationale
Risk of bias in individual studies	6-7	14	Describe anticipated methods for assessing risk of bias of individual studies, including whether this will be done at the outcome or study level, or both; state how this information will be used in data synthesis
Data synthesis	7-8	15a	Describe criteria under which study data will be quantitatively synthesised
	8	15b	If data are appropriate for quantitative synthesis, describe planned summary measures, methods of handling data and methods of combining data from studies, including any planned exploration of consistency (such as I^2, Kendall's τ)
	8	15c	Describe any proposed additional analyses (such as sensitivity or subgroup analyses, meta-regression)
	8	15d	If quantitative synthesis is not appropriate, describe the type of summary planned
Meta-bias(es)	7	16	Specify any planned assessment of meta-bias(es) (such as publication bias across studies, selective reporting within studies)
Confidence in cumulative evidence	7	17	Describe how the strength of the body of evidence will be assessed (such as GRADE)

*** It is strongly recommended that this checklist be read in conjunction with the PRISMA-P Explanation and Elaboration (cite when available) for important clarification on the items. Amendments to a review protocol should be tracked and dated. The copyright for PRISMA-P (including checklist) is held by the PRISMA-P Group and is distributed under a Creative Commons Attribution Licence 4.0.**

From: Shamseer L, Moher D, Clarke M, Ghersi D, Liberati A, Petticrew M, Shekelle P, Stewart L, PRISMA-P Group. Preferred reporting items for systematic review and meta-analysis protocols (PRISMA-P) 2015: elaboration and explanation. BMJ. 2015 Jan 2;349(jan02 1):g7647.

For peer review only
